# Combining Valence-to-Core X-ray Emission and Cu K-edge X-ray Absorption Spectroscopies to Experimentally Assess Oxidation State in Organometallic Cu(I)/(II)/(III) Complexes

**DOI:** 10.1021/jacs.1c09505

**Published:** 2022-01-20

**Authors:** Blaise L. Geoghegan, Yang Liu, Sergey Peredkov, Sebastian Dechert, Franc Meyer, Serena DeBeer, George E. Cutsail

**Affiliations:** † Max Planck Institute for Chemical Energy Conversion, Stiftstrasse 34-36, 45470 Mülheim an der Ruhr, Germany; ‡ Institute of Inorganic Chemistry, University of Duisburg-Essen, Universitätsstrasse 5-7, 45117 Essen, Germany; § Institute of Inorganic Chemistry, University of Göttingen, Tammannstrasse 4, 37077 Göttingen, Germany

## Abstract

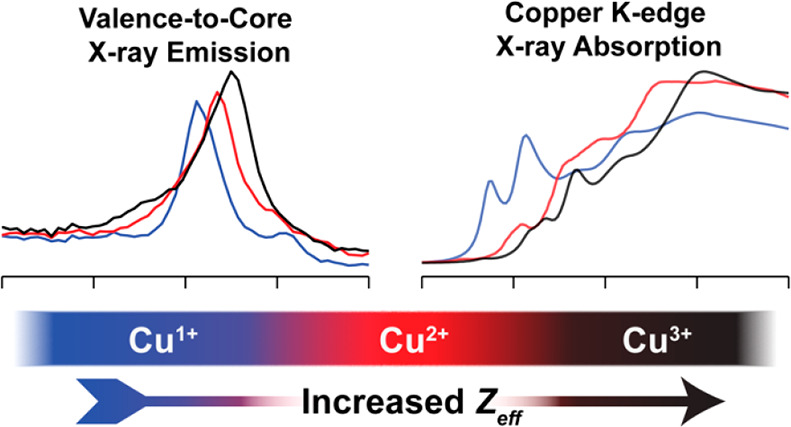

A series of organometallic copper complexes in formal oxidation states ranging from +1 to +3 have been characterized by a combination of Cu K-edge X-ray absorption (XAS) and Cu Kβ valence-to-core X-ray emission spectroscopies (VtC XES). Each formal oxidation state exhibits distinctly different XAS and VtC XES transition energies due to the differences in the Cu Z_eff_, concomitant with changes in physical oxidation state from +1 to +2 to +3. Herein, we demonstrate the sensitivity of XAS and VtC XES to the physical oxidation states of a series of N-heterocyclic carbene (NHC) ligated organocopper complexes. We then extend these methods to the study of the [Cu(CF_3_)_4_]^−^ ion. Complemented by computational methods, the observed spectral transitions are correlated with the electronic structure of the complexes and the Cu Z_eff_. These calculations demonstrate that a contraction of the Cu 1s orbitals to deeper binding energy upon oxidation of the Cu center manifests spectroscopically as a stepped increase in the energy of both XAS and Kβ_2,5_ emission features with increasing formal oxidation state within the [Cu*n*+(NHC_2_)]^*n+*^ series. The newly synthesized Cu(III) cation [Cu^III^(NHC_4_)]^3+^ exhibits spectroscopic features and an electronic structure remarkably similar to [Cu(CF_3_)_4_]^−^, supporting a physical oxidation state assignment of low-spin d^8^ Cu(III) for [Cu(CF_3_)_4_]^−^. Combining XAS and VtC XES further demonstrates the necessity of combining multiple spectroscopies when investigating the electronic structures of highly covalent copper complexes, providing a template for future investigations into both synthetic and biological metal centers.

## Introduction

Copper’s ability to facilitate a wide-range of catalytic processes has made it a highly popular metal for homogeneous catalysis. Copper has been successfully employed as a catalyst in C–C bond formation, C–H bond activation and is especially prevalent in biological oxidation and oxygenation processes.^1^ Many of the proposed pathways for homogeneous copper-mediated reactions invoke redox couples including not only Cu^I^ and Cu^II^ but also the Cu^III^ ion;^2−6^ therefore, understanding the spectroscopic markers that distinguish these three physical oxidation states of Cu from one another is important in order to determine reaction mechanisms in both synthetic and biological settings. Within the literature, the assignment of a Cu(III) oxidation state to catalytic intermediates^7^ has been met with a degree of controversy due to the decreasing calculated metal 3d admixture in the frontier molecular orbitals (MOs) of high-valent late transition metals.^8−10^ For instance, the physical oxidation state and electronic structure of [Cu(CF_3_)_4_]^−^ anion has been debated for decades.^10−14^ The diamagnetic and colorless complex has been structurally and spectroscopically characterized as a closed-shell *S* = 0 species with distorted *D*_2*d*_ symmetry. One may describe this complex as it was originally done: a formal Cu^III^ center ligated by four monodentate, monoanionic CF_3_^–^ ligands.^15^ Alternatively, a d^10^ (Cu^I^) electronic configuration can be assigned, where the ligands assume either a heterogeneous 3(CF_3_^–^) + CF_3_^+^ or homogeneous 4(CF_3_^0.5–^) oxidation state.^11,16^ To assign a formal Cu(I) oxidation state, a select definition of an “inverted ligand field” is used, where the MOs with >50% Cu 3d admixture sink to energies below those with mostly ligand admixture. The resultant lowest unoccupied molecular orbital (LUMO) in this picture is therefore predominately of ligand parentage and a supposed 3d^10^ electronic configuration for [Cu(CF_3_)_4_]^−^ is thus invoked.^11^ The electronic structure of copper centers like the [Cu(CF_3_)_4_]^−^ anion have led to unique^14^ and differing^17^ spectroscopic interpretations.

As an atom-specific probe by the means of core electron excitation, X-ray absorption spectroscopy (XAS) is frequently employed to determine oxidation states.^18−20^ The edge energy shifts in both K (1s) and L (2s,p) edge XAS or X-ray photoemission spectroscopy (XPS) often correlate well to the core orbital ionization energy.^9,21−30^ For copper, XAS has proven itself a reliable probe of copper’s oxidation state^19,20,31,32^ and has been used to show large shifts in spectra for Cu(I), Cu(II), and Cu(III) species.^18,20^ For formally assigned Cu(III) species, consistent shifts to higher energies have been observed in both K and L-edge XAS, indicating an increased effective nuclear charge (Z_eff_) at the Cu upon oxidation. However, decreased L-edge intensity of formal Cu(III) centers has been reinterpreted to support a Cu(I) physical oxidation state assignment where an inverted field is present.^10^ This is in contrast to the previously established interpretation of increased metal–ligand covalency for the high oxidation-state, yielding decreased copper 3d character in the frontier orbitals.^28^

**Scheme 1:**
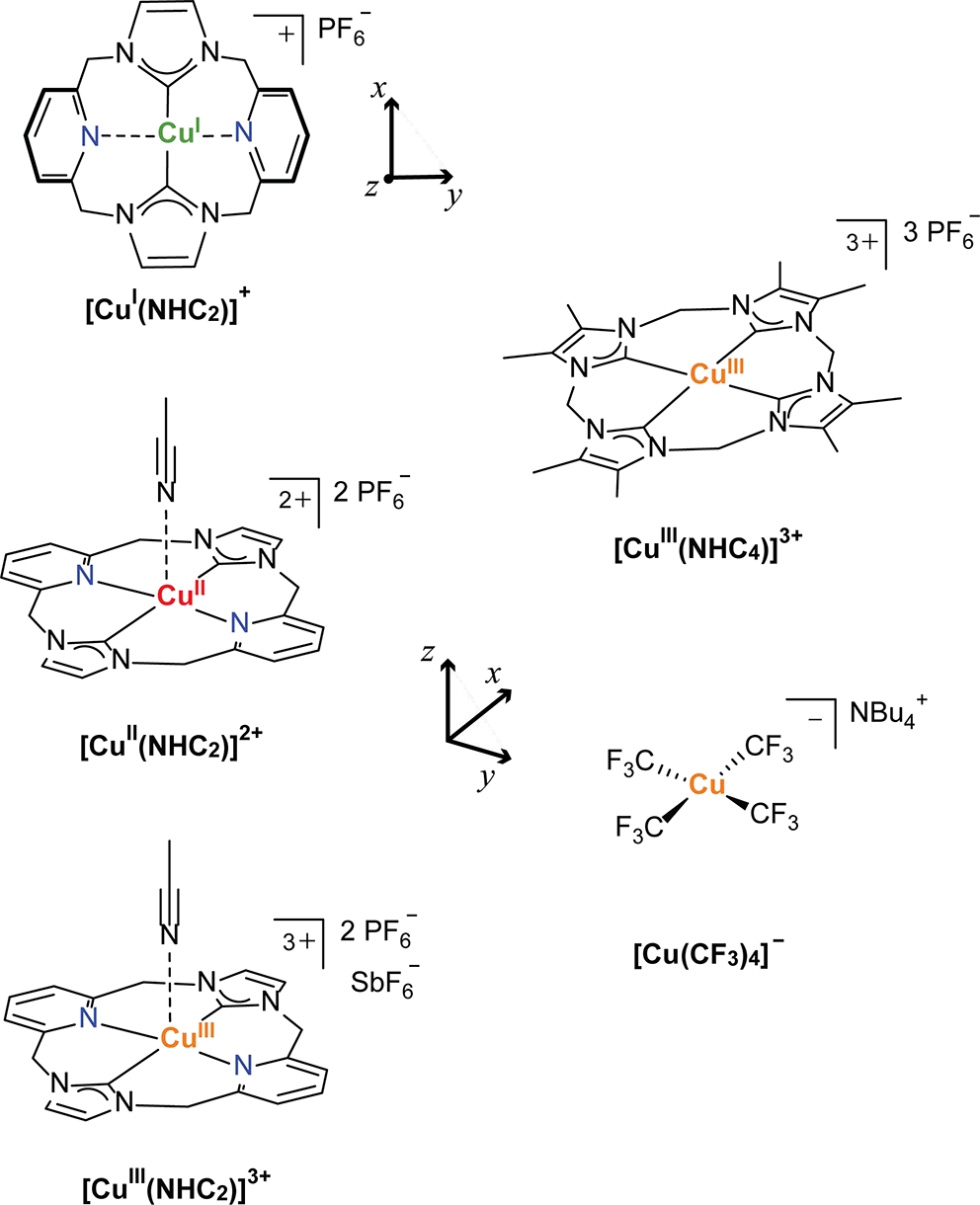
Organometallic Copper Complexes Investigated in the Current Work

We previously reported the high-resolution Cu K-edge XAS of a series of copper +1, +2, and +3 complexes ligated by a neutral, tetradentate 16-atom macrocyclic ligand with *trans* N-heterocyclic carbene (NHC) donors and *trans* pyridine donors (Scheme 1).^32^ Here, the flexibility of the pyridine donors of the macrocycle allows the Cu(I) species to adopt an approximately linear two-coordinate environment, whereas in the Cu(II) and Cu(III) centers both the pyridyl and NHC groups coordinate to the Cu center with a roughly square-planar CNCN first coordination sphere, accompanied by a distant (weakly bound) apical solvent (MeCN) ligation. The strong NHC σ donors of the ligand and their high covalency with the copper center are speculated to aid in the stabilization of the high-valent Cu(III) ion. Supported by X-ray crystallography, UV–vis spectroscopy, electrochemical characterization, and theoretical approaches, the physical oxidation states of copper in this redox series were clearly assigned via shifts in the energy of the pre-edges and edge features of their Cu K-edge XAS.^32^

**Figure 1:**
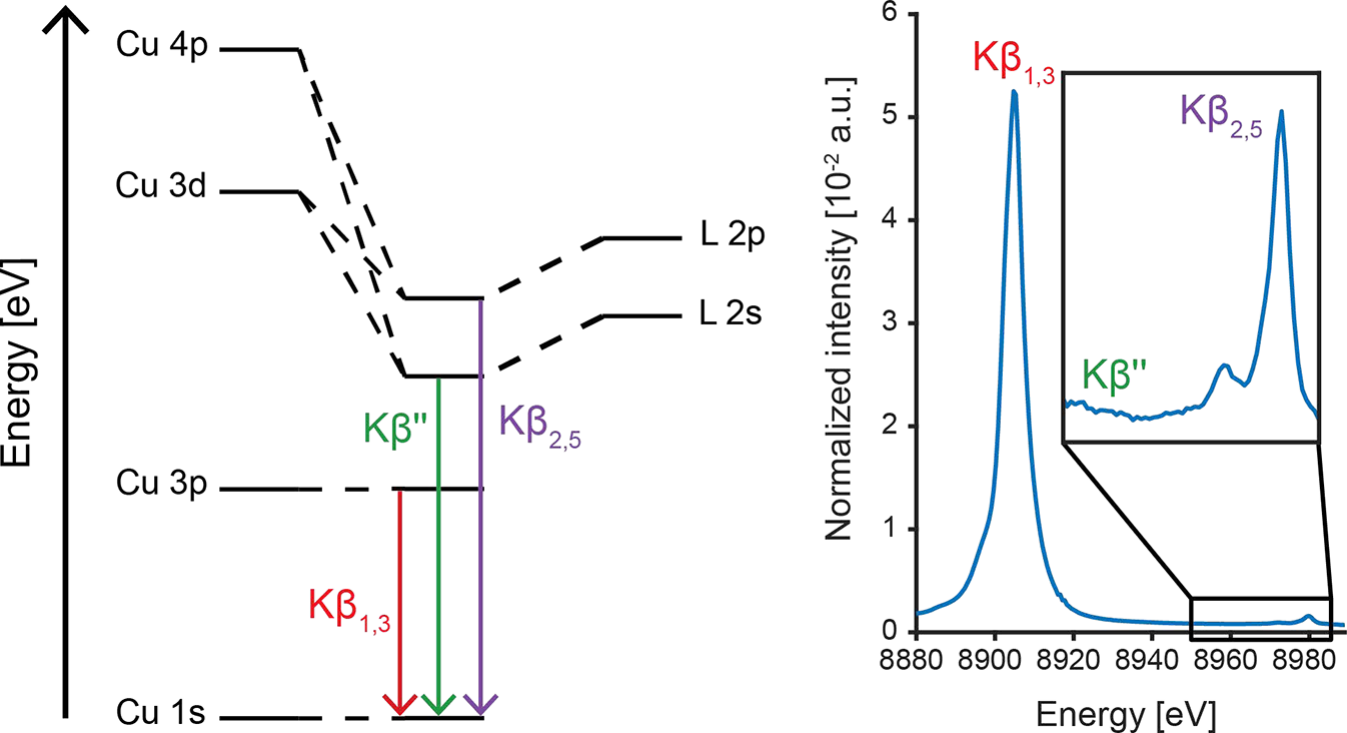
Simplified MO diagram schematically depicting the origin of the transitions in the Cu Kβ XES spectrum (left) and the experimental Cu Kβ XES spectrum of [Cu^III^(NHC_4_)]^3+^ with VtC region enlarged approximately 100-fold (right).

The paramagnetic nature of Cu(II) complexes allows for their extensive characterization through EPR and/or nuclear resonance spectroscopy, which provides a rich picture of both the electronic and geometric structures. However, for the EPR silent Cu(I) and Cu(III) species, complementary techniques to XAS that offer additional insight into the electronic structure of the Cu centers are limited. X-ray emission spectroscopy (XES) results from photon emission due to the relaxation of electrons from orbitals with principle quantum numbers *n* > 1 into the 1s core hole produced by a high-energy incident X-ray beam (Figure 1).^33^ The Cu Kβ XES spectrum is comprised of two main regions of interest. At lower energy, the mainline region (∼8905 eV) originates from the refilling of the core 1s hole by Cu 3p electrons.^25,34^ It is well demonstrated for other 3d (and 4d) transition metals that the XES mainlines are excellent reporters of 3d (or 4d) electronic structure due to the role of p-d electron spin exchange interactions on the spectrum.^34−36^ The mainlines of both small molecule and protein-bound Cu(I) centers have been more recently reported.^37^ Resonant inelastic X-ray scattering (RIXS) measurements have enabled more detailed analysis of less featured Cu mainlines, leading to distinct assignments of charge-transfer and multielectron transitions achieved at higher resonant excitation energies, corresponding to previously proposed metal–ligand charge transfer (MLCT) features of Cu(I) centers.^38^ Classically, nonresonant Kβ mainline spectra have been used for the fingerprinting of transition metal spin states;^39,40^ however, covalency has been shown to complicate this simple picture.^35,41^ We note, however, that Cu Kβ mainlines have yet to be thoroughly explored as reporters of oxidation and/or spin state, although the copper mainline is reasoned to follow the same qualitative trends as other d^*n*^ > 5 transition metals.

At higher energy, but with lowest transition probability and oscillator strength, the valence-to-core (VtC) region (∼8975 eV) is subdivided into the Kβ″ and Kβ_2,5_ features, originating from the refilling of the core 1s hole by MOs of predominantly ligand *n*s and *n*p character, respectively.^33,42−44^ Hence, VtC XES directly probes the energy of the valence MOs with respect to the metal 1s orbital and has been shown to be highly sensitive to ligand type,^38,45−47^ bond activation,^45,48,49^ protonation state,^50,51^ and ligand orientation.^37,38,49^ Despite the fact that VtC spectroscopy has been shown to be sensitive to the oxidation state in other first-row transition metals such as Mn^39^ and Fe,^52^ it has not yet been employed to aid in the determination of oxidation state in Cu centers.

For the electronic structure of organocopper complexes, we hypothesized that VtC XES will offer new spectroscopic insight for understanding of copper’s electronic structure. It has been demonstrated that strong metal–ligand covalency can complicate oxidation state assignments based on Cu K-edge XAS alone.^18^ Therefore, employing multiple core X-ray spectroscopies to interrogate the electronic structure of such high-valent Cu systems may provide observable trends that are related to physical oxidation states. These spectroscopic observables form the foundations for such analysis of highly covalent systems, for which computational methods may give an ambiguous picture.

Given the highly covalent nature of both NHC and CF_3_^–^ ligands, we present here the series of NHC/pyridine macrocycle complexes as a calibrant for the use of VtC XES as an additional experimental technique in conjunction with Cu K-edge XAS in the determination of physical oxidation state and for understanding the nature and origins of the observed transitions. Additionally, for a systematic extension of the NHC-bearing macrocyclic systems toward the homoleptic C_4_ first coordination sphere, more analogous to [Cu(CF_3_)_4_]^−^, a new tetra-NHC Cu(III) macrocyclic complex [Cu^III^(NHC_4_)]^3+^ has been prepared and included in this study. [Cu^III^(NHC_4_)]^3+^ is similar to the tetra-NHC Cu(III) complex recently reported by the Kühn group,^53^ but has backside methyl substituents at the four imidazol-2-yliden groups (Scheme 1). The relationships and trends extracted from the investigation of the four NHC-bearing complexes are then applied to both the VtC XES and Cu K-edge XAS of the [Cu(CF_3_)_4_]^−^ complex in order to determine its physical oxidation state and electronic structure. The combination of the complementary XAS and VtC XES techniques is shown to be crucial for assessing the complicated electronic structures of highly covalent Cu complexes, overcoming the limitations of analyzing results from either technique in isolation. Throughout this report, we will emphasize the importance of correlating observables across multiple measurement techniques and how this forms a firm foundation for interpretation of complicated electronic structures, especially in highly covalent systems.

## Results

**Scheme 2:**
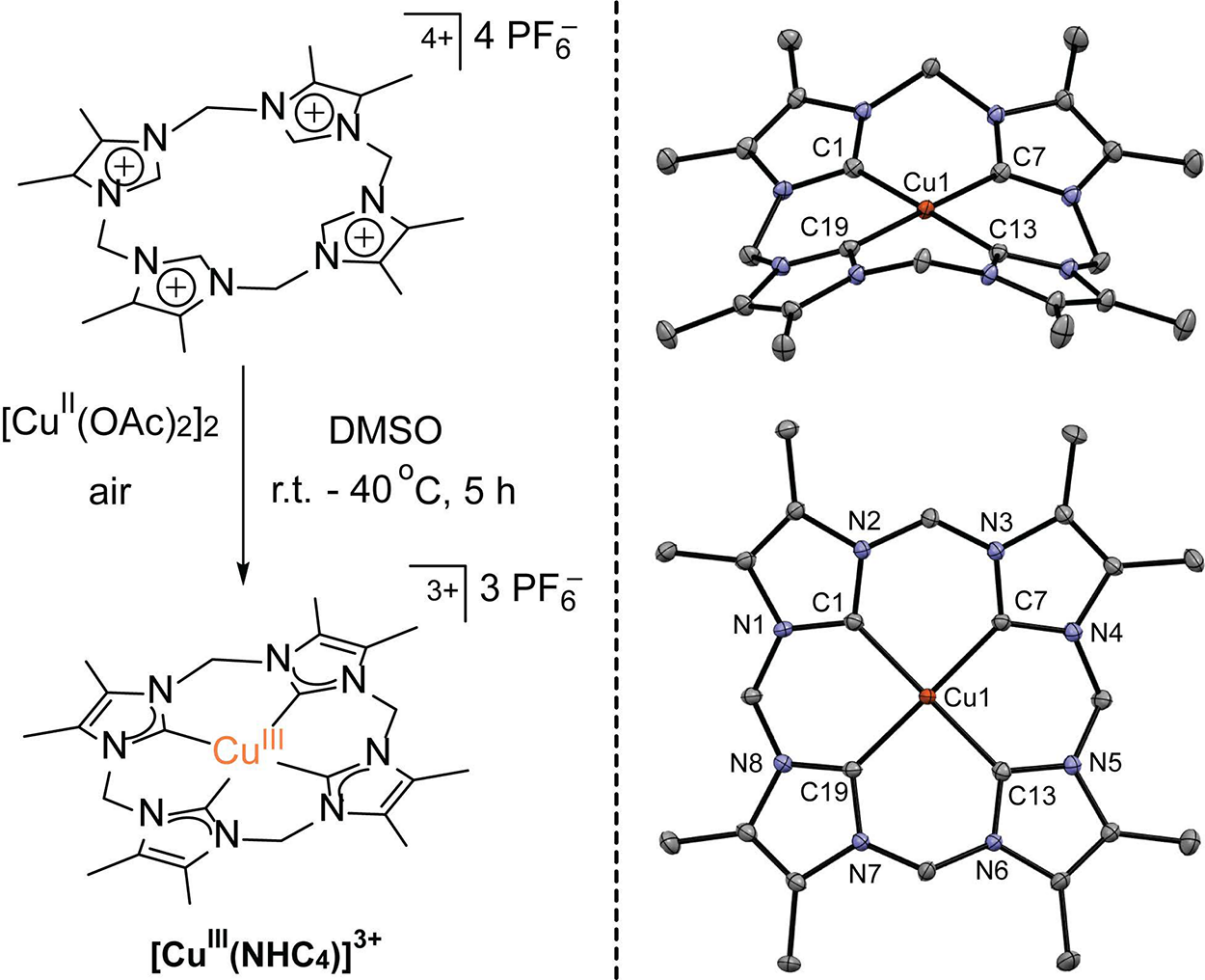
Synthesis of the New Tetra-NHC Ligated Cu(III) Complex (left) and the Molecular Structure of Its Cation [Cu^III^(NHC_4_)]^3+^ in Solid State (Right; Side View and Top View)

The complexes of general formula [Cu^*n*+^(NHC_2_)]^*n*+^ form a Cu redox series of a macrocyclic ligand bearing *trans* carbene and *trans* pyridyl donor groups with a central Cu(I)/(II)/(III) ion.^32^ It was previously shown that the redox state of the ligand remains fixed throughout the series, but in [Cu^II^(NHC_2_)]^2+^ and [Cu^III^(NHC_2_)]^3+^ an acetonitrile molecule is weakly bound in an apical site.^32^ The new complex [Cu^III^(NHC_4_)]^3+^ is closely related to [Cu^III^(NHC_2_)]^3+^, but the two pyridyl donors are substituted for NHC groups and the backbones of the imidazole-2-ylidenes are methylated. [Cu^III^(NHC_4_)]^3+^ was synthesized according to the method that was originally described by Ghavami et al.^53^ for the nonmethylated parent analogue, with slight modifications (Scheme 2, left; see Supporting Information for details of the synthetic protocols). The PF_6_^–^ salt of [Cu^III^(NHC_4_)]^3+^ was fully characterized by various spectroscopic methods, CHN analysis as well as by single crystal X-ray diffraction (see SI for detailed information and spectroscopic data; crystallographic parameters and refinement details are given in Table S2, and selected bond lengths and angles for the [Cu^III^(NHC_4_)]^3+^ cation are given in Table S3). The molecular structure of [Cu^III^(NHC_4_)]^3+^ in the solid state (Scheme 2 and Figure S16) reveals no binding of acetonitrile in the apical position, although two acetonitrile solvent molecules are present in the asymmetric unit of the crystal lattice. The primary C_4_ coordination sphere in [Cu^III^(NHC_4_)]^3+^ is almost perfectly square planar (sum of angles around Cu is 360.0°), and the Cu–C^NHC^ bond distances are in the range 1.877(3)–1.889(3) Å (average 1.88 Å) and thus very similar to those of [Cu^III^(NHC_2_)]^3+^ (average 1.88 Å). The overall macrocycle conformation is nonplanar and saddle-shaped, but variable-temperature NMR (VT-NMR) spectra show facile conformational inversion of the macrocyclic ligand in MeCN-*d*_3_ solution (with activation parameters *ΔH*^‡^ = 17.8 ± 0.2 kcal mol^–1^, *ΔS*^‡^ = 19.0 ± 0.8 cal mol^–1^ K^–1^ derived from an Eyring plot; see SI for details) with apparent *D*_4*h*_ symmetry on the NMR time scale at room temperature. NMR data (Figures S2–S11) and SQUID magnetometry (Figure S15) confirm that [Cu^III^(NHC_4_)](PF_6_)_3_ is diamagnetic over the entire temperature range studied.

### Cu K-edge XAS of NHC Complexes

**Figure 2:**
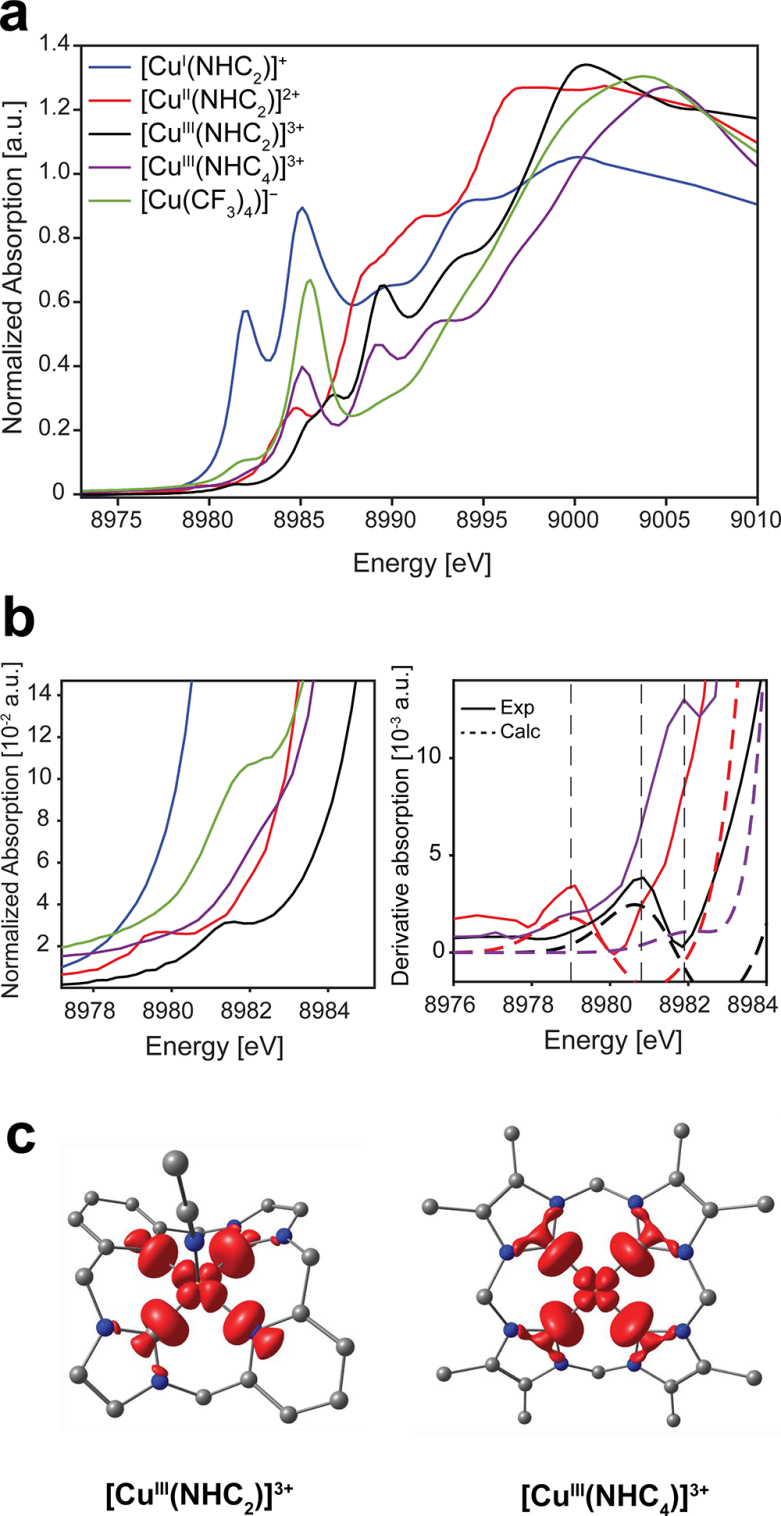
(a) Cu K-edge XAS (transmission mode) spectra for all compounds (solid lines). (b) Expansion of pre-edge region (left) and first derivatives of experimental and TDDFT calculated (B3LYP/ZORA-def2-TZVP/ZORA) pre-edge region for the [Cu^II^(NHC_2_)]^2+^, [Cu^III^(NHC_2_)]^3+^, [Cu^III^(NHC_4_)]^3+^ species. Vertical dashed lines are included as visual guides. (c) Transition difference density plots for the pre-edge transition in [Cu^III^(NHC_2_)]^3+^ and [Cu^III^(NHC_4_)]^3+^ plotted at an isosurface value of 0.003 au. Legend: C, gray; Cu, orange; N, blue. Hydrogen atoms omitted for clarity.

The Cu K-edge XAS is presented initially as a metric for both physical oxidation state of the copper center, as well as the metal–ligand covalency. The Cu K-edge Kβ high-energy resolution fluorescence detected (HERFD) XAS spectra of the [Cu^*n*+^(NHC_2_)]^*n*+^ series has been discussed in detail previously.^32^ In parallel to those measurements, the more typical transmission mode XAS data were also collected and are now displayed in Figure 2a and Figure S17. Transmission mode Cu K-edge XAS was obtained for [Cu^III^(NHC_4_)]^3+^ and [Cu(CF_3_)_4_]^−^ at the SAMBA beamline at the SOLEIL synchrotron (see SI for sample and collection details), and the results are compared to the [Cu^*n*+^(NHC_2_)]^*nn*^ series (Figure 2a). The spectrum of the [Cu(CF_3_)_4_]^−^ species is a very good match to that previously reported.^14^

The [Cu^I^(NHC_2_)]^+^ complex exhibits an absorption profile unlike the Cu(II) and Cu(III) species due to its d^10^ electronic configuration, and thus lack of a classic 1s → 3d pre-edge transition. The lowest energy feature in the XAS of [Cu^I^(NHC_2_)]^+^ is an electric dipole allowed 1s → 4p_*z*_ transition at 8982.0 eV and is followed by an even more intense feature at 8985.1 eV, which is likely attributed to a 1s → 4p_*y*_ excitation based on molecular symmetry (Scheme 1).^32^ To higher energy is a poorly resolved feature at 8989.5 eV, and the white line maximum is observed at ∼8994 eV.

[Cu^II^(NHC_2_)]^2+^ and [Cu^III^(NHC_2_)]^3+^ both exhibit formally electric dipole forbidden 1s → 3d pre-edge transitions at ∼8979.5 and 8981.3 eV, respectively. While [Cu^III^(NHC_4_)]^3+^ lacks an isolated pre-edge feature of similar energy to its [Cu^III^(NHC_2_)]^3+^ analogue, it does exhibit a more intense shoulder at ∼8982.3 eV, approximately 1 eV higher than [Cu^III^(NHC_2_)]^3+^, that is most clearly observed in the first derivative spectrum shown in Figure 2b, which can be putatively assigned as a 1s to 3d pre-edge transition. This is slightly higher than the typical 8981 ± 0.5 eV range observed for other Cu(III) systems^20^ and suggests that the exchange of pyridyl donors for strong σ donating carbenes in the macrocyclic tetra-NHC ligand significantly influences the 1s-LUMO gap (antibonding interaction with the Cu 3d_*x*_^_2_^_–y_^_2_^) in the resulting Cu(III) complex (*vide infra*). The [Cu^III^(NHC_4_)]^3+^ spectrum is highly featured and exhibits both similarities and differences to that of the bis-NHC analogue [Cu^III^(NHC_2_)]^3+^. [Cu^III^(NHC_4_)]^3+^ exhibits three main, intense features in the rising edge at approximately 8985.0, 8989.2, and 8993.0 eV, with a shoulder at ∼8997.0 eV before the white line is observed at ∼9005 eV. Both [Cu^III^(NHC_2_)]^3+^ and [Cu^III^(NHC_4_)]^3+^ exhibit similar edge positions, which are shifted ∼5 eV higher than [Cu^II^(NHC_2_)]^2+^, supporting increased oxidation state assignments as the effective 1s binding energy increases for the [Cu^III^(NHC_2_)]^3+^ and [Cu^III^(NHC_4_)]^3+^ relative to the Cu(II) complex. Relative to that of the Cu(I) species, the Cu(II) and Cu(III) NHC complexes exhibit white lines of both higher energy and higher intensity, in line with the increased oxidation state: [Cu^II^(NHC_2_)]^2+^ 8996.5 eV; [Cu^III^(NHC_2_)]^3+^ 9000.0 eV; [Cu^III^(NHC_4_)]^3+^ 9005.0 eV. However, differences between the ligands (i.e., pyridine vs carbene) may also play a role here, as [Cu^III^(NHC_2_)]^3+^ exhibits a slightly sharper lower energy white line than its [Cu^III^(NHC_4_)]^3+^ analogue. Apart from the higher energy pre-edge transition in [Cu^III^(NHC_4_)]^3+^ compared to [Cu^III^(NHC_2_)]^3+^, the main difference between the two spectra is that the [Cu^III^(NHC_4_)]^3+^ exhibits an intense and well-resolved feature at ∼8985.0 eV, isoenergetic with the most intense rising edge feature in the Cu(I) species [Cu^I^(NHC_2_)]^+^, whereas [Cu^III^(NHC_2_)]^3+^ does not. However, the intensity of the 8985 eV feature of the [Cu^III^(NHC_4_)]^3+^ spectrum is significantly less than the Cu(I) feature and is comparable to the previously assigned 1s → 4p features of [Cu^III^(NHC_2_)]^3+^.^32^ The energy of both the pre-edge and white line features of the [Cu^III^(NHC_4_)]^3+^ complex are distinctly different from that of the [Cu^I/II^(NHC_2_)]^+/2+^ species, but with sufficient similarities to [Cu^III^(NHC_2_)]^3+^ in order to make a Cu(III) assignment.

As previously demonstrated for [Cu^II^(NHC_2_)]^2+^ and [Cu^III^(NHC_2_)]^3+^, TDDFT calculations of the Cu K-edge spectrum exhibit a single pre-edge transition into an MO that is clearly of copper 3d_*x*_^_2_^_–y_^_2_^ parentage and their energy differences are consistent with the deeper binding energy of the Cu 1s electrons in the higher valent systems.^32^ Extending these calculations to [Cu^III^(NHC_4_)]^3+^ predicts that the pre-edge transition in tetra-NHC complex [Cu^III^(NHC_4_)]^3+^ is shifted into the rising edge by ∼0.9 eV compared to the equivalent transition in [Cu^III^(NHC_2_)]^3+^, lending support to the notion that modulation of the LUMO energy is achieved through increased covalency. In fact, [Cu^III^(NHC_2_)]^3+^ and [Cu^III^(NHC_4_)]^3+^ exhibit nearly identical transition state difference densities, as shown in Figure 2c. It is clear from the inspection of the XAS alongside the TDDFT that the classic 1s → 3d_*x*^2^–y^2^_ transition for [Cu^III^(NHC_4_)]^3+^ is quite high in energy, even approaching the edge structure. The observed trend of increased pre-edge energies with increased metal–ligand covalency is in line with other organocopper complexes, and the substitution of C-donor atoms into the first coordination sphere has been demonstrated to aid in the stabilization of the Cu(III) ion.^54^ TDDFT calculations also predict with good accuracy the energy separation of the pre-edge feature and the Cu 1s → 4p_*z*_ and Cu 1s → 4p_x,y_ transitions in [Cu^III^(NHC_4_)]^3+^ as shown in Figure S19.

### Cu K-edge XAS of [Cu(CF_3_)_4_]^−^

Overall, the absorption profile of the [Cu(CF_3_)_4_]^−^ complex has some similarities with those of the Cu(III) macrocyclic NHC complexes, especially the [Cu^III^(NHC_4_)]^3+^ complex, despite fundamentally different ligand environments. The pre-edge transition in [Cu(CF_3_)_4_]^−^ occurs at ∼8982 eV (Figure 2 and Figure S19), similar to that observed in [Cu^III^(NHC_4_)]^3+^ and slightly higher in energy than in [Cu^III^(NHC_2_)]^3+^. This suggests a correlation between higher metal–ligand covalency and increased energy of the pre-edge transition, implying that the four CF_3_^–^ ligands form a more covalent interaction with the Cu center than only two NHCs and two pyridines, but perhaps not as covalent as the four NHCs of the [Cu^III^(NHC_4_)]^3+^ complex.

To higher energy, the 8985.6 eV feature exhibited by [Cu(CF_3_)_4_]^−^ has been ascribed to Cu 1s → 4p_*z*_ excitations,^14^ and is more intense than the *ca*. 8985 eV transition of the [Cu^III^(NHC_4_)]^3+^ species, which has a similar 1s → 4p_*z*_ transition (Figures S19–20 and Table S4). The less pronounced features in the rising edge of [Cu(CF_3_)_4_]^−^ at ∼8989.5 and 8994.0 eV generally have nearly isoenergetic counterparts in both the [Cu^III^(NHC_2_)]^3+^ and [Cu^III^(NHC_4_)]^3+^ species ∼0.3 eV lower in energy. Additionally, the white line in the [Cu(CF_3_)_4_]^−^ complex is intermediate to that of [Cu^III^(NHC_2_)]^3+^ and [Cu^III^(NHC_4_)]^3+^, mirroring the trend in the pre-edge energies and thus the potential covalency trend. Despite the similarities in the spectra of the [Cu(CF_3_)_4_]^−^ and [Cu^III^(NHC_4_)]^3+^ complexes, there are key differences when compared to the [Cu^III^(NHC_2_)]^3+^ complex: chiefly, the much more intense 1s → 4p_*z*_ transition at ∼8985 eV and the much lower intensity feature at 8989.5 eV. This is unsurprising due to the differences in ligand types and molecular geometries (Scheme 1). Noteworthily, the pre-edge feature and 1s → 4p_*z*_ feature of [Cu(CF_3_)_4_]^−^ are isoenergetic with the lowest energy and the most intense absorption features of the Cu(I) species [Cu^I^(NHC_2_)]^+^, respectively. However, the low intensity of the pre-edge feature and higher energy rising edge and white line of the [Cu(CF_3_)_4_]^−^ species relative to that of the Cu(I) species suggest significant d-hole character as well as a significantly increased Cu 1s ionization energy, further supporting a Cu(III) assignment based on the experimental data.^18^

TDDFT calculations reproduce the Cu K-edge XAS for [Cu(CF_3_)_4_]^−^, showing that the pre-edge transition is due to an excitation of a core 1s electron into what is clearly a d_*x*_^_2_^_–*y*_^_2_^ localized Cu orbital with σ bonding to the CF_3_^–^ ligands (Figure S19). The calculated pre-edge transition difference density plots for [Cu(CF_3_)_4_]^−^ and [Cu^III^(NHC_4_)]^3+^ exhibit remarkably similar characteristics, suggesting a similar LUMO electronic structure of the two complexes. Additionally, the LUMO in [Cu(CF_3_)_4_]^−^ transforms as b_2_ in approximate *D*_2*d*_ symmetry, meaning that mixing of the Cu 4p_*z*_ orbital into the d_*x*_^_2_^_–*y*_^_2_^ is allowed by symmetry, thus increasing the intensity of the pre-edge feature through reinstating partial electric dipole allowed character to the transition.

The clear assignment of the Cu(I), (II) and (III) oxidation states in the [Cu^*n*+^(NHC_2_)]^*n*+^ series via their Cu K-edge XAS can be used here as a guide for evaluation of the oxidation state of the Cu ions in both the [Cu^III^(NHC_4_)]^3+^ and [Cu(CF_3_)_4_)]^−^ complexes. In combination with the crystallographic characterization, the Cu K-edge XAS strongly supports the high valent +3 oxidation state of Cu in the [Cu^III^(NHC_4_)]^3+^ species. Additionally, the similarity of many features in the spectra of the macrocyclic NHC-based Cu(III) complexes and the [Cu(CF_3_)_4_)]^−^ species is consistent with a high valent Cu(III) center in the latter. To corroborate this hypothesis, the Cu Kβ VtC spectra of these compounds were obtained and are discussed below.

### Cu Kβ XES Mainlines

**Figure 3:**
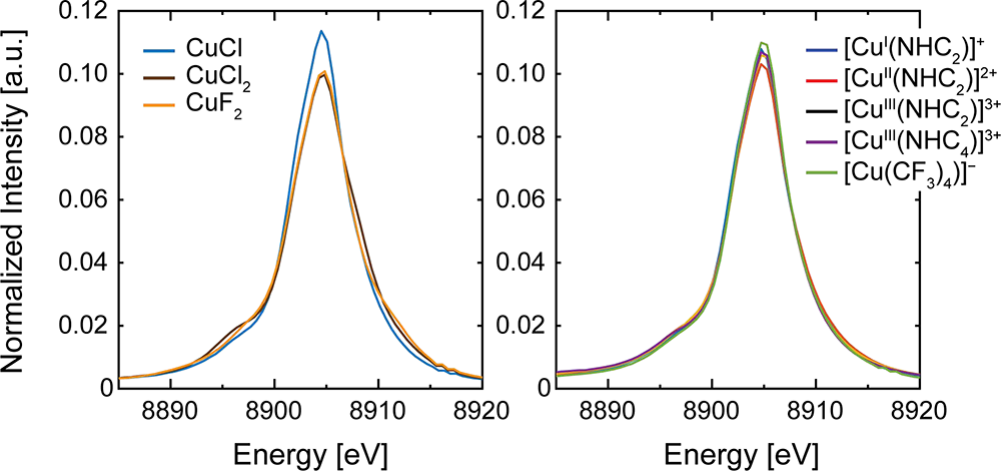
Cu Kβ XES mainlines. Kβ XES spectra have been normalized by setting the integral of the mainline region to a value of 1.0 au.

As discussed in the introduction, X-ray emission spectroscopies, particularly, Kβ mainlines, have been shown to be diagnostic of the metal oxidation state and spin state in other 3d transition metals. Variations in the Kβ mainline features arise from metal 3p–3d exchange in the final states.^39^ However, the utility of Kβ mainline emission spectroscopy as a probe for the oxidation state of copper is lacking. To initially explore the utility of Kβ mainlines for the assignment of copper oxidation and spin states, the Kβ XES of various copper halides were collected (Figure 3). The Kβ mainlines of anhydrous CuCl, CuCl_2_, and CuF_2_ all exhibit very similar Kβ_1,3_ energies (8904.6–8904.7 eV), with no significant shifts between the various copper oxidation states. A slightly more intense Kβ′ shoulder at 8894.7 eV is observed for the CuCl_2_ sample compared to CuCl, as expected due to the additional 3d hole for the Cu^II^ center. The energy splitting of the Kβ_1,3_ and Kβ′ features (Δ*E*) is a marker of metal–ligand covalency,^35^ as is also the intensity of the Kβ′ feature. For CuF_2_, the energy of the Kβ′ feature increases while the intensity decreases, yielding a diminished mainline splitting of Δ*E* ≈ 8.2 eV, approximately 1.7 eV less than the splitting for CuCl_2_ (Table S5). The variation of the splitting here demonstrates that the covalency of the copper center has a potentially significant influence on the appearance of the Kβ mainline for samples of the same formal oxidation state. However, as previously shown in Fe Kβ mainline studies, these covalency interactions can also counteract the formal oxidation and spin state effects, often making the interpretation of Kβ mainlines difficult.^35^

The Kβ mainline XES spectra for [Cu^I/II/III^(NHC_2_)]^1/2/3+^, [Cu^III^(NHC_4_)]^3+^, and [Cu(CF_3_)_4_]^−^ are also shown in Figure 3. Despite the clear variation in Cu oxidation state observed by XAS, all [Cu^*n*+^(NHC_2_)]^*n*+^ complexes exhibit almost superimposable Kβ mainlines and no shifts in the mainline Kβ_1,3_ peak’s energy (∼8904.9 eV), suggesting that no such analysis can be conducted in this instance. As opposed to the maximum splitting mainline differences observed for the copper halides, the [Cu^*n+*^(NHC_*x*_)]^*n*+^ series and [Cu(CF_3_)_4_]^−^ complex all exhibit similar Δ*E* values (Table S5) and a much smaller splitting range (<0.6 eV). While it has often been shown that Kβ mainlines are excellent reporters of electronic configuration in ionic metal salts with lower d^*n*^ electron counts, the lack of differences for the reported Cu complexes is unsurprising given their more complex electronic configuration and the generally featureless Kβ mainline of more “ideal” copper halide salts.^35^ The remarkable similarities of these disparate formal Cu oxidation states is reminiscent of the similar Kβ emission spectra for covalent iron centers.^41,55^ This suggests that the mainlines of these highly covalent molecular systems are poor indicators of the Cu oxidation (and spin) state. In the next section, the feasibility and sensitivity of Cu VtC XES as a probe of copper oxidation state are investigated.

### Cu Kβ VtC of Macrocyclic NHC Complexes

**Figure 4:**
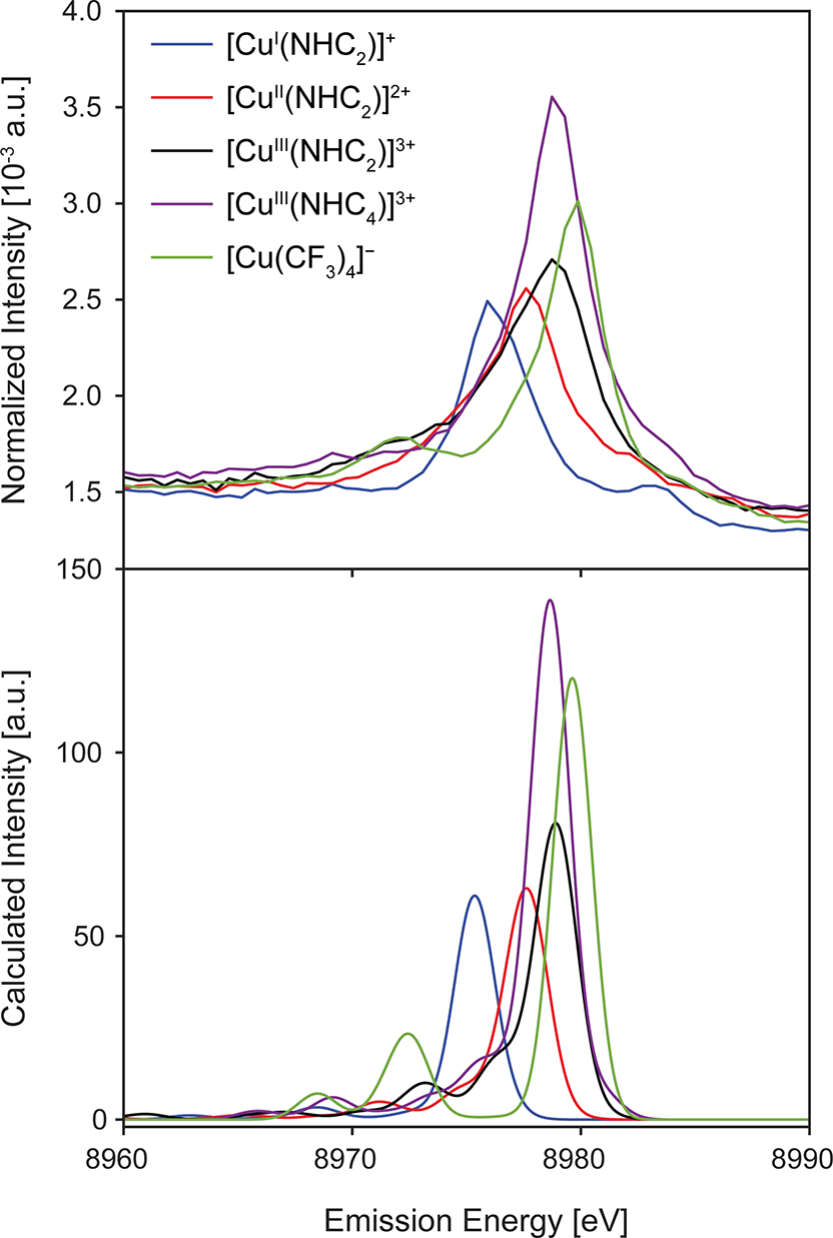
Experimental (top) and calculated (bottom) VtC XES spectra for the [Cu^*n*+^(NHC_2_)]^*n*+^ series, [Cu^III^(NHC_4_)]^3+^, and [Cu(CF_3_)_4_]^−^. Calculated spectra were shifted −5.1 eV and a 2.0 eV (full-width half-maximum) Gaussian broadening was applied.

The VtC spectrum of the Cu(I) species [Cu^I^(NHC_2_)]^+^ is dominated by a single, intense feature at 8975.9 eV, which increases in energy to 8977.6 eV and finally to 8978.7 eV for [Cu^II^(NHC_2_)]^2+^ and [Cu^III^(NHC_2_)]^3+^, respectively (Figure 4). This suggests that the transition energies in the VtC XES spectra are sensitive to the oxidation state of the copper atom in this series where the ligand environment remains consistent. The intensities of features in the Kβ_2,5_ region of the emission spectrum have been previously correlated to metal–ligand covalency.^33,43,47^ The fitted VtC spectra show that the intensity of the Kβ_2,5_ region predominantly arises from one high intensity emission band (Figure S20); however, the intensity of this individual emission band does not increase from Cu(I) to Cu(II) and then Cu(III) to mirror the total emission intensity, which is likely due to differences in molecular structure concomitant with oxidation of the Cu center (*vide infra*).

In the Cu(II) and Cu(III) species, a shoulder grows into the VtC spectrum on the low energy side of the main Kβ_2,5_ feature (Figure S20) which is not observed in the Cu(I) species—a likely result of pyridine coordination and axial acetonitrile ligands in the high valent species (*vide infra*).^32^ To higher energy of the main VtC features, between ∼8982–8983 eV, there are emission features with noticeable intensity that are not assigned as part of the Kβ_2,5_ feature. Overlays of the VtC XES with the XAS of the [Cu^*n*+^(NHC_2_)]^*n*+^ series (Figure S21) show that these high energy VtC XES transitions overlap with the weak pre-edge transitions of the XAS and are, thus, greater than the Fermi energy. VtC XES and XAS in the single electron transition picture probe the occupied and unoccupied molecular orbitals, respectively. Hence in this simple picture, no transition overlap is expected between the two spectroscopies. However, features above the Fermi energy have also been observed in Zn VtC XES and resonant VtC XES studies of Cu(I) suggesting that these higher energy VtC XES peaks likely arise from multielectron transitions.^38,56^

[Cu^III^(NHC_4_)]^3+^ exhibits an intense VtC peak at approximately the same energy as the analogous [Cu^III^(NHC_2_)]^3+^ complex, albeit with much greater intensity (Figure 4). As in the [Cu^*n*+^(NHC_2_)]^*n*+^ series, the spectrum of the [Cu^III^(NHC_4_)]^3+^ species is dominated by a single intense transition feature that is isoenergetic (8979 eV) with the dominant feature of the [Cu^III^(NHC_2_)]^3+^ analogue (Figure S20 and Table S6). The dramatic increase in intensity from [Cu^III^(NHC_2_)]^3+^ to [Cu^III^(NHC_4_)]^3+^ is initially attributed to the increased number of carbene donors present: two vs four. NHC groups are significantly stronger sigma donors^57−59^ than pyridyl nitrogens and thus result in more covalent metal–ligand interactions and greater mixing of Cu *n*p character into the valence MOs.

In the [Cu^*n*+^(NHC_2_)]^*n*+^ series, the Cu–C distances shorten from Cu(I):1.94 Å to Cu(II):1.91 Å to Cu(III):1.88 Å, as expected.^32^ In the Cu(I) species, the pyridines are essentially noncoordinating, with long Cu–N distances of ∼2.6 Å; however, in the Cu(II) and Cu(III) species the two pyridines are coordinated, each with Cu–N distances of ∼2.16 and 1.97 Å, respectively (Figure S22). The coordination of the weaker interacting pyridines is anticipated to bring about similarly weak, although significant, contributions to the VtC emission intensity in comparison to the carbene donors (*vide infra*). Furthermore, inspection of the C–Cu–C angles in these complexes shows that the angle in the Cu(I) species (173.9°) and the Cu(III) species (172.7°) are closer to linear than that in the Cu(II) species (169.0°). The more linear C–Cu–C σ bonds in the Cu(I) and Cu(III) species will have better overlap with the Cu *n*p_*x*_ orbitals, thus resulting in larger intensity being imparted into the transition. This shows that the decreasing Cu–C bond lengths in the Cu(II) species relative to the Cu(I) species are counteracted by the geometry changes resulting from the oxidation of the Cu center. Such an angular dependence on the VtC spectrum of hydroxo and oxo-bridged Fe dimers, as well as Ni-NO_*x*_ and Cu-NO_*x*_ (*x* = 1,2) complexes, has been demonstrated previously.^49,60^ Hence, the increase in the emission intensity observed in Cu(II) and Cu(III) may be due to a combination of shorter Cu–C(carbene) bond lengths, coordination of pyridine groups resulting in significant overlap with the Cu *n*p_*y*_ orbitals, and increased linearity in the C^NHC^–Cu–C^NHC^ bond vector facilitating further Cu *n*p_*x*_ mixing into the valence orbitals.^60^ It is clear from the experimental data of the [Cu^*n*+^(NHC_2_)]^*n*+^ redox series that the VtC XES is sensitive to the oxidation state of the Cu centers, manifesting as a stepwise increase in energy of the dominant emission feature in the Kβ_2,5_ region.

To elucidate the origin of the changes in emission intensities, DFT calculations were used to determine the contributions of the ligand donor groups to the VtC spectra. Ground-state DFT methods have been demonstrated to accurately reproduce experimental XES spectra by calculating the valence orbital → 1s energy separations.^33,39,45,47,51^Figure 4 shows the experimental and calculated VtC spectra for all five complexes. For [Cu^III^(NHC_4_)]^3+^ and [Cu(CF_3_)_4_]^−^, the singlet state was calculated to be significantly lower in energy than the triplet state as expected for high valent copper, in agreement with the experimental findings for [Cu^III^(NHC_4_)]^3+^ (*vide supra*) and similar to the singlet spin state determined for [Cu^III^(NHC_2_)]^3+^ (Table S7).^14,17,32^

The calculated VtC spectra for the [Cu^*n*^^+^(NHC_2_)]^*n+*^ series exhibit Kβ_2,5_ features with maxima at 8975.9, 8977.6, and 8978.7 eV for the Cu(I), Cu(II) and Cu(III) species, respectively. The corresponding experimental maxima occur at 8975.9 eV, 8977.2 and 8978.5 eV, showing good agreement between experiment and theory (Figure S23). The DFT XES calculations reproduce the increase in intensity of the Kβ_2,5_ feature as the oxidation state of the Cu center increases—in-line with the experimental data shown in Figure S20. Furthermore, DFT predicts the growth of a shoulder on the low energy side of the main Kβ_2,5_ feature with increased oxidation state, which is also observed experimentally as a broadening to the lower energy side of the most intense VtC feature (Figure S20). Inspection of the DFT-calculated Cu 1s orbital energies shows that there is a stepwise contraction of the core orbital energies with increased physical oxidation state, decreasing by 2.7 eV from Cu(I) to Cu(II) and a further 2.1 eV from Cu(II) to Cu(III). This change in energy explains the stepwise increase in the VtC Kβ_2,5_ feature, as the core orbitals of the Cu center are more strongly influenced by the increased Z_eff_ of the high valent ion compared to the valence orbitals. Hence, the energy gap between the MOs giving rise to the dominant VtC transitions and the Cu 1s systematically increases with physical oxidation state.

**Figure 5:**
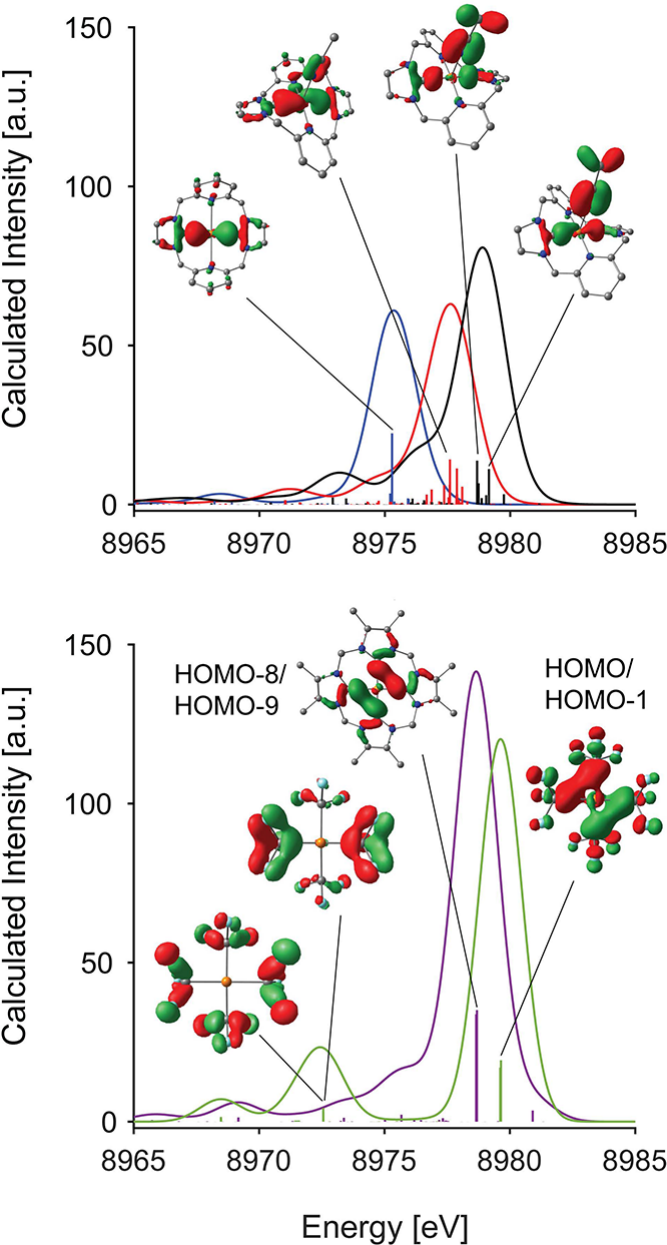
Calculated VtC spectra and selected transition donor MOs for [Cu^I^(NHC_2_)]^+^ (blue), [Cu^II^(NHC_2_)]^2+^ (red), [Cu^III^(NHC_2_)]^3+^ (black), [Cu^III^(NHC_4_)]^3+^ (purple), and [Cu(CF_3_)_4_]^−^ (light green). MOs are plotted at an isosurface value of 0.05 au.

The single electron transition nature of the DFT VtC XES calculations allows for individual transitions to be assigned to the corresponding donor molecular orbitals.^33,39^Figure 5 shows the calculated VtC spectra for the [Cu^*n*+^(NHC_2_)]^*n*+^ series annotated with the donor MOs for the most intense transitions of interest (additional MO assignments are shown in Figure S24). The Cu(I) species is best described as a linear two-coordinate molecule with local *D*_2*h*_ symmetry, meaning that the metal–ligand bonding interactions are comparatively simplified—the intense Kβ_2,5_ VtC transitions arise from donor MOs that are predominantly C^NHC^ p_*x*_ with optimal overlap to the acceptor Cu *n*p_*x*_ (Cu-L σ). The bonding MO of the carbene presents predominant Cu-L σ overlap with the Cu d_*xz*_ and a minor π p_*z*_ contribution (Figure S25). The large amount of Cu *n*p_*x*_ orbital admixture yields the intense dipole allowed feature, whereas the Cu-L π interaction does not present favorable Cu p_*z*_ overlap to produce appreciable dipole intensity. The effect of this low-coordinate geometry for the Cu(I) species on spectral intensity is also observed in the XAS, exhibiting an intense pair of 1s → 4p transitions at 8981.8 and 8984.8 eV in the pre-edge (Figure 2a)—resulting from an energetic splitting of the Cu p_*x*_ and p_*y*_ pairs as there is effectively no ligation along the *y*-axis from the pyridyl moieties. [Cu^II^(NHC_2_)]^2+^ and [Cu^III^(NHC_2_)]^3+^ have coordinating pyridine ligands and distort slightly from the higher symmetry observed in the Cu(I) complex. Both the additional ligands and geometrical distortion yield many more transitions, which distributes the Cu *n*p character across multiple final states;^18^ however, all dominant transitions exhibit Cu–C^NHC^ σ-character (Figure 5 and S10), as identified in [Cu^I^(NHC_2_)]^+^.

This analysis is supported by the calculated polarized VtC spectra, which show that for the [Cu^*n*+^(NHC_2_)]^*n*+^ series, almost all the intensity arises along the C–Cu–C^NHC^*x* vector, with subtle contributions originating along the N^py^–Cu–N^py^*y* vector for the Cu(II) and Cu(III) species, concomitant with pyridine coordination (Figure S26), and in agreement with the assertions made from the analysis of the experimental VtC fits (Figure S20) and donor MOs calculated by DFT (Figure S27). Conversely, [Cu^III^(NHC_4_)]^3+^ exhibits nearly equivalent VtC intensity along both *x* and *y* C^NHC^–Cu–C^NHC^ vectors as expected.

Integration of the VtC spectra shows an ∼25% increase in intensity upon oxidation of [Cu^II^(NHC_2_)]^2+^ to [Cu^III^(NHC_2_)]^3+^, which is mostly due to the increase in intensity of the main Kβ_2,5_ feature as a result of better overlap between the C^NHC^ 2p_*x*_ and Cu *n*p_*x*_ from a more linear C^NHC^–Cu–C^NHC^ bond vector and shorter Cu–C^NHC^ distances, rather than broadening or additional features, as shown by the change in intensity of the dominant transition in the fits of the spectra (Table S6 and Figure S20). Notably, there is a significant increase in the amount of ligand *n*p admixture going from the Cu(II) (63.5%) to the Cu(III) (70.0%) species (Table S8). The increase in the ligand *n*p admixture in the valence MOs as the Cu(II) species is oxidized is expected to lead to improved ligand-mediated mixing of the Cu *n*p character into the valence MOs, imparting a larger amount of electric dipole allowed character and, in turn, leading to increased emission intensity. Furthermore, the geometric distortions caused by coordination of the pyridine groups in the Cu(II) and Cu(III) species clearly increases total metal–ligand interaction and enables additional overlap of the ligand 2p and out-of-plane Cu *n*p_*z*_ orbitals. Therefore, a mixture of shortening Cu–C bond distances, additional Cu-pyridine interaction, and ligand-based geometric distortions combine to explain the changes in intensity observed between oxidation states for complexes in the [Cu^*n*+^(NHC_2_)]^*n*+^ series.

It is evident from the polarized VtC spectra that substitution of the two pyridyl moieties in [Cu^III^(NHC_2_)]^3+^ with additional NHCs in [Cu^III^(NHC_4_)]^3+^ results in a more intense VtC spectrum. The tetra-NHC complex [Cu^III^(NHC_4_)]^3+^ exhibits almost ideal local *D*_4*h*_ symmetry in the CuC_4_ first coordination sphere (Table S3), with the principal axis perpendicular to the equatorial plane formed by the four carbene ligands (Scheme 2 and Figure S16). For this reason, almost all of the intensity in the Kβ_2,5_ region stems from two degenerate donor MOs formed by the C 2p_*x*_ and 2p_*y*_ like orbitals of adjacent NHC moieties (Figure 5). Here, the mixing of Cu *n*p character into the valence MOs is achieved predominantly through C^NHC^–C^NHC^ σ bonding MOs which form an *e* doublet with very good overlap with the Cu *n*p_*x*_ and *n*p_*y*_ orbitals (Figure 5).

The experimental VtC data shown here demonstrate that the energy of the VtC emission is clearly sensitive to the oxidation state at copper. Additionally, ground-state DFT methodologies demonstrate the intensity of the emission is the product of both the amount of metal–ligand covalency and the symmetry of the complex ion. This series of complexes of the neutral macrocyclic NHC-bearing ligands thus forms a convenient calibration series for analysis of similarly covalent Cu species such as [Cu(CF_3_)_4_]^−^.

### Cu Kβ VtC of [Cu(CF_3_)_4_]^−^

The Cu Kβ VtC spectrum of [Cu(CF_3_)_4_]^−^ exhibits a predominant single feature to significantly higher energy and intensity than both the [Cu^I^(NHC_2_)]^+^ and [Cu^II^(NHC_2_)]^2+^ species, and ∼1.2 eV higher in energy than both the Cu(III) NHC-based complexes (Figure 4). The [Cu(CF_3_)_4_]^−^ complex has an intensity that is between the limits of [Cu^III^(NHC_2_)]^3+^ and [Cu^III^(NHC_4_)]^3+^ and also has a well resolved feature to lower energy at ∼8972 eV that is not observed in the Cu(III) NHCs. The fact that the C–Cu–C angle (*x*-direction) in [Cu(CF_3_)_4_]^−^ is more acute (164.9°) than in [Cu^III^(NHC_2_)]^3+^ (172.7°), but exhibits more intense VtC emission, suggests that the 4CF_3_^–^ donor set is more covalent than the py_2_NHC_2_ set. However, it is clear that per ligand, the CF_3_^–^ ligand produces less intensity than the carbene ligand, after comparison to the VtC emission intensity of the [Cu^III^(NHC_4_)]^3+^ complex. Typically, the amount of metal *n*p character that mixes into the valence MOs increases with decreasing metal–ligand bond length. The average Cu–C bond lengths in [Cu(CF_3_)_4_]^−^ (1.96 Å) are significantly longer than in [Cu^III^(NHC_4_)]^3+^ (1.88 Å), further indicating that the CF_3_^–^ ligand is in fact a less covalent ligand and poorer σ-donor than the NHC. The increase in energy of the Kβ_2,5_ feature in [Cu(CF_3_)_4_]^−^ relative to the NHC complexes is likely due to the ionic nature of the CF_3_^–^ ligands, increasing the relative energies of the donor MOs to the Cu 1s orbital as a result of increased local negative charge. The differences between the NHC and CF_3_^–^ ligand types is also expected to explain the observation of a much more intense and well-resolved feature to the low energy side of the dominant Kβ_2,5_ feature in [Cu(CF_3_)_4_]^−^. Based on the experimental data, the VtC XES supports the observations in the Cu K-edge XAS and supports a high valent, highly covalent Cu(III) center in [Cu(CF_3_)_4_]^−^.

DFT calculations were performed in order to elucidate the nature of the VtC XES transitions and shed light on the origins of the similarities and differences between the emission profiles of the [Cu(CF_3_)_4_]^−^ complex and those of the NHC-based complexes. The higher symmetry and homoleptic C_4_ first coordination sphere of the [Cu^III^(NHC_4_)]^3+^ (*D*_4*h*_) and [Cu(CF_3_)_4_]^−^ (*D*_2*d*_) complexes yields a narrower distribution of final states for the donor MOs (Figure 5) than the [Cu^III^(NHC_2_)]^3+^ complex. For [Cu^III^(NHC_4_)]^3+^ and [Cu(CF_3_)_4_]^−^, two effectively isoenergetic transitions from degenerate orbitals of an *e* doublet (including both α and β spin manifolds) are responsible for almost all the intensity of the main Kβ_2,5_ feature (Figure 5). Despite the clear differences of the neutral NHC and anionic CF_3_^–^ ligands, both complexes exhibit remarkably similar valence MO structures. Analysis of the atomic contributions to the donor MOs producing the intense feature (Table S8) reveals that there is 9.2 and 10.1% Cu *n*p admixture in [Cu^III^(NHC_4_)]^3+^ and [Cu(CF_3_)_4_]^−^, respectively, further confirming the similarity in properties between the two complexes. Additionally, this is significantly larger than the ∼3.5–7.3% calculated in the [Cu^*n*+^(NHC_2_)]^*n+*^ series (Table S8).

The donor MOs in both the Cu(III) complexes with C_4_ ligand donor sets exhibit significant delocalization across the first coordination sphere in the *x*- and *y*-directions, allowing for a large degree of Cu *n*p_*x*__,__*y*_ mixing, as shown by the significant *x*- and *y*-polarized intensity in Figure S26. As stated above, the ionic nature of the CF_3_^–^ ligands increases the energies of the donor MOs relative to the Cu 1s orbital: the dominant VtC transitions in both the [Cu^III^(NHC_4_)]^3+^ and [Cu(CF_3_)_4_]^−^ complexes arise from HOMO–8/HOMO–9 in the former and HOMO/HOMO–1 in the latter (Figure 5 and Table S4). The fact that these highly similar donor MOs are frontier orbitals in the [Cu(CF_3_)_4_]^−^ complex, but slightly “buried” in the [Cu^III^(NHC_4_)]^3+^ complex explains the higher energy Kβ_2,5_ feature in the [Cu(CF_3_)_4_]^−^ complex relative to the NHC-bearing complexes.

In the experimental spectrum of [Cu(CF_3_)_4_]^−^, a unique and sharp lower energy feature at ∼8972 eV is well resolved (Figure 4). DFT calculations show that this feature arises almost exclusively from π-interactions between fluorine 2p_*x*__,__*y*_ and carbon 2p_*x*__,__*y*_ of the CF_3_^–^ ligands, which are ideally positioned to form σ-type interactions with the Cu atom along the *x*- and *y*-directions (Figure 5). These MOs exhibit significant overlap with the Cu *n*p_*x*__,__*y*_ orbitals, resulting in ∼3.1% Cu p character and ∼88.8% total ligand p character, which ultimately explains the increased intensity in this region compared to the NHC-based complexes, which exhibit closer to ∼1.0% Cu *n*p admixture into the donor MOs in this region.

The great similarities between the both the XAS and VtC XES of the [Cu^III^(NHC_4_)]^3+^ and [Cu(CF_3_)_4_]^−^ complexes, coupled with theory, suggest a similar electronic structure across both complexes. Furthermore, the [Cu^*n*+^(NHC_2_)]^*n+*^ series and [Cu^III^(NHC_4_)]^3+^ complexes exhibit clear sensitivity to the copper’s physical oxidation state, where the observed shifts in energy of the main Kβ_2,5_ features are a function of the deepening of the Cu 1s orbital through increased Z_eff_.

## Discussion

### Relationship between Cu 1s Ionization Energy and Oxidation State

**Figure 6:**
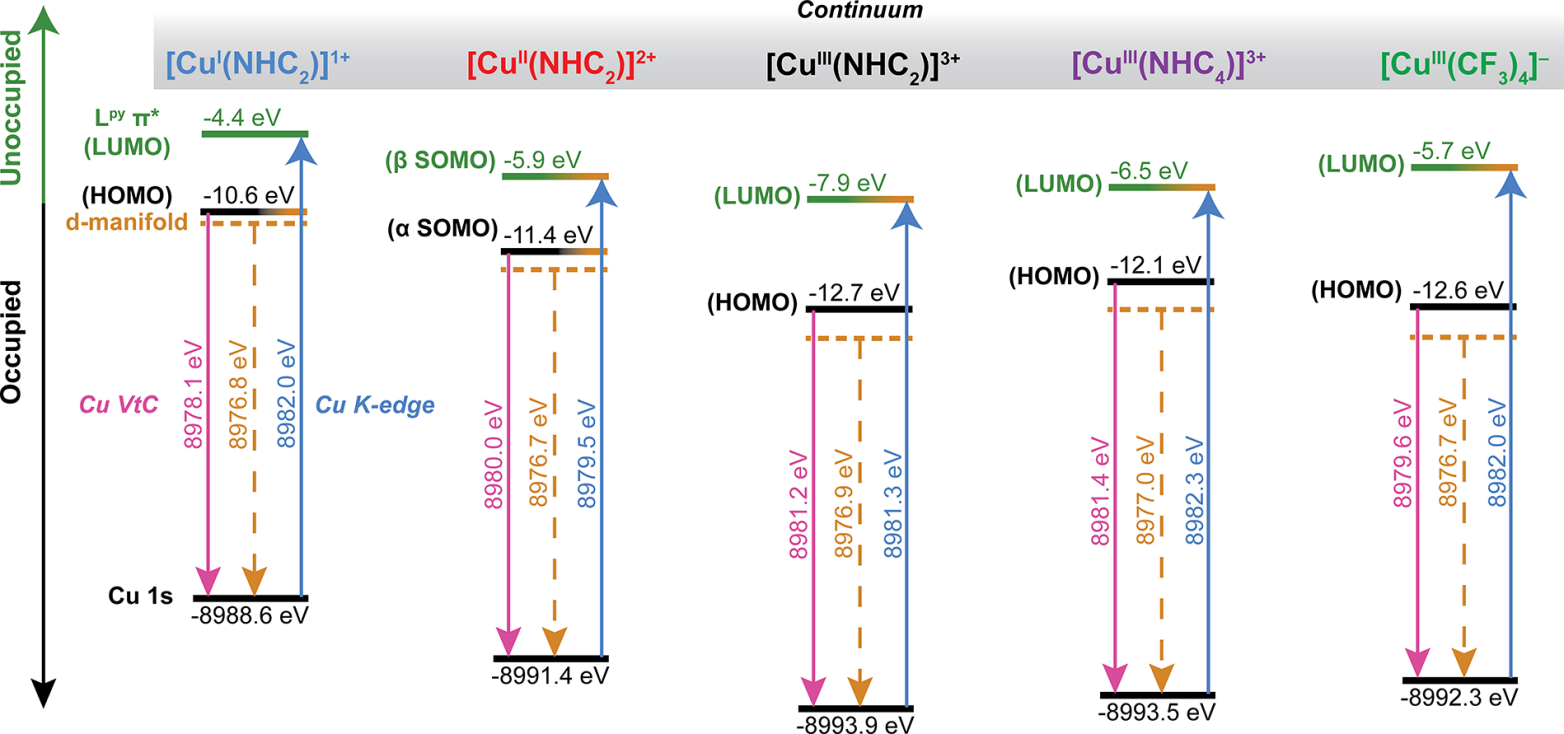
Qualitative representation of the electronic structures of the complexes discussed in the present analysis. Dashed orange lines correspond to the IWAE of the quadrupole-only VtC spectrum and represent the approximate energetic center of the Cu 3d manifold. Solid pink lines represent the HOMO energy determined from the DFT-calculated Cu Kβ VtC spectra. Solid blue lines represent the LUMO energy as determined experimentally from the Cu K-edge XAS. The relative energies of the Cu 1s and HOMO for each complex are shown in black and were determined from DFT calculations (B3LYP/ZORA-def2-TZVP/ZORA), whereas the relative energy of the LUMO shown in green was determined from TDDFT calculations (B3LYP/ZORA-def2-TZVP/ZORA).

For the [Cu^*n*+^(NHC_2_)]^*n+*^ series, the VtC data presented in the current study are in-line with the previously reported Cu K-edge XAS data, exhibiting clear spectroscopic energy shifts correlated to the formally assigned oxidation state.^32^ These trends are mirrored by the Cu VtC XES spectra of the [Cu^*n*+^(NHC_2_)]^*n*+^ series, where the dominant Kβ_2,5_ feature increases incrementally in energy with increasing physical oxidation state (Figure 4). Having demonstrated that the XAS pre/rising edge features and VtC XES data are accurately reproduced by (TD)DFT calculations, we can use the calculated electronic structures to better understand the origins of the energy shifts in the XAS and VtC spectra. This combined XAS/XES approach enables the formation of a more complete electronic structure diagram (Figure 6) and correlation of the spectroscopic trends to the relative energies of the core Cu 1s orbital and the valence MOs.

While [Cu^III^(NHC_2_)]^3+^ and [Cu^III^(NHC_4_)]^3+^ are both assigned as Cu(III) centers, their pre-edge energies differ by approximately 1 eV. Inspection of the relative orbital energies shows that the Cu 1s orbital of the [Cu^III^(NHC_4_)]^3+^ species is ∼0.5 eV higher in energy than in the [Cu^III^(NHC_2_)]^3+^ analogue, however, it is still nearly 2 eV lower than the Cu(II) 1s orbital energy of [Cu^II^(NHC_2_)]^2+^. The LUMO of [Cu^III^(NHC_4_)]^3+^ is shifted ∼1.5 eV higher in energy than the [Cu^III^(NHC_2_)]^3+^ species; the combined 1s and LUMO energy difference yield the ∼1 eV higher energy shift of the [Cu^III^(NHC_4_)]^3+^ pre-edge transition compared to [Cu^III^(NHC_2_)]^3+^. This demonstrates how the more covalent interactions of the NHC_4_ vs py_2_NHC_2_ first coordination spheres with the Cu center modulates the pre-edge transition energy as a function of the increased relative energy of the 3d_*x*_^_2_^_–*y*_^_2_^–2p_*x*__,__*y*_ σ* antibonding orbital (Figure S18), despite minimally raising the energy of the Cu 1s orbital.

The [Cu(CF_3_)_4_]^−^ complex exhibits a similar XAS spectrum to that of both [Cu^III^(NHC_2_)]^3+^ and [Cu^III^(NHC_4_)]^3+^ (Figure 2), with a Cu 1s orbital energy calculated to be significantly more negative than that of the established Cu(II) center in [Cu^II^(NHC_2_)]^2+^. The observed energy of the pre-edge feature in the XAS of [Cu(CF_3_)_4_]^−^ is intermediate to [Cu^III^(NHC_2_)]^3+^ and [Cu^III^(NHC_4_)]^3+^, which may be interpreted as increased metal–ligand covalency across the ligand series such that py_2_NHC_2_ < 4CF_3_^–^ < NHC_4_. The modulation of the observed transition energies is in-line with the anticipated increase in ligand field resulting from these strong σ donating ligands. This trend is also observed in the VtC emission intensities, which appear to increase with increased metal–ligand covalency (Figure 5).

As mentioned above, (TD)DFT methods show that the XAS and VtC XES spectroscopic trends in the [Cu^*n*+^(NHC_2_)]^*n+*^ series are due to the contraction of the Cu 1s orbital to deeper binding energy upon oxidation of the central Cu ion: the Cu 1s orbital energy decreases by 2.8 eV between the Cu(I) and Cu(II) species, and by a further 2.5 eV between the Cu(II) and Cu(III) species (Figure 6). The significant contraction of the core Cu 1s orbitals compared to the more subtle contraction of the frontier/valence MOs explains the increasing energy of the Cu K-edge pre-edges and VtC emission energies of the Kβ_2,5_ features for the [Cu^*n*+^(NHC_2_)]^*n*+^ series. Similarly, the Cu 1s binding energy in both [Cu^III^(NHC_4_)]^3+^ and [Cu(CF_3_)_4_]^−^ is calculated to be to 4.9 and 3.7 eV lower in energy than in [Cu^I^(NHC_2_)]^+^, respectively, supporting the notion that the spectroscopic features and their relative energies are due to the increase in Cu Z_eff_ and its powerful capacity to reflect the copper ion’s formal oxidation state. These results strongly support a physical Cu(III) oxidation state and low-spin 3d^8^ electronic configuration for the [Cu(CF_3_)_4_]^−^ complex, even with the strong charge donation from the anionic CF_3_^–^ ligands. The [Cu^III^(NHC_2_)]^3+^ and [Cu^III^(NHC_4_)]^3+^ complexes have Cu 1s orbitals that are 1.7 and 1.2 eV lower in energy than that of the [Cu(CF_3_)_4_]^−^ complex, respectively, which is likely due to the fact that the neutral macrocyclic bis-NHC and tetra-NHC ligands provide less charge donation to the high valent Cu centers than the CF_3_^–^ ligands.

This combined XAS/VtC XES approach allows for evaluation of the relative energies of the LUMO and valence MOs as a function of the Z_eff_ of the Cu, as demonstrated in Figure 6, which emphasizes the impact of the physical oxidation state on the core X-ray spectroscopies employed in this study. While the 3d orbitals do not contribute significant intensity to the VtC XES, the average energy of this manifold may be extracted from the quadrupole (3d → 1s) contribution of the calculated VtC XES spectra (Figure S28). For the Cu(I) species [Cu^I^(NHC_2_)]^+^, the 3d manifold is represented by the five highest occupied MOs (Figure 6 and Figure S24), which are easily identified. However, in the Cu(II) and Cu(III) species the increased metal–ligand covalency and coordination number causes the highest valence MOs to have significantly more ligand character, resulting in MOs that do not reflect the localized geometries of the spherical harmonics that are typically associated with “pure” 3d orbitals. However, analysis of the quadrupole contribution to the VtC XES effectively reports the distribution of the 3d orbital character without the need for MO analysis. The effective VtC transition energy of the 3d manifold is plotted in Figure 6, and when viewed on the absolute energy scale, it is clear that as oxidation state increases across the [Cu^*n*+^(NHC_2_)]^*n*+^ series, the 3d manifold and the 1s core orbital are found to exhibit deeper binding energy. Accompanying the decreasing 3d manifold energy with Cu 1s orbital contraction is a relative expansion of the 3d manifold, where the energy range of the observed quadrupole transitions increases, and is most obvious to higher energies (Figure S28). The dominant VtC XES transition observed for these organocopper complexes arises from MOs to the higher energy edge of the d-manifold. A consistent picture develops from the [Cu^*n*+^(NHC_2_)]^*n+*^ series, where the increased dispersion of d-orbital character across the valence MOs is brought about by an increased Cu physical oxidation state and concomitant changes in metal–ligand covalency. Considering the [Cu^III^(NHC_4_)]^3+^ complex, the quadrupole-only VtC spectrum is highly similar to the [Cu^III^(NHC_2_)]^3+^ analogue, and noticeably different from the Cu(I) and Cu(II) species. The [Cu^III^(NHC_2/4_)]^3+^ complexes exhibit quadrupolar intensity at higher energies than in the Cu(I) or Cu(II) species, and the dominant feature in the spectrum is less well-resolved—in-line with a distribution of the d-orbital character across a larger number of MOs. These observations support the trends in the experimental VtC XES and XAS, and by using this combined experimental approach, these complexes form a useful calibration series for the determination of both physical oxidation state and metal–ligand covalency in the [Cu^III^(NHC_4_)]^3+^ and [Cu(CF_3_)_4_]^−^ complexes. Given the aforementioned calibrations, we can now rationalize both the Cu K-edge XAS and VtC XES of the [Cu(CF_3_)_4_]^−^ complex in terms of its physical oxidation state and proposed electronic configurations.

### Physical Oxidation State of [Cu(CF_3_)_4_]^−^

The Cu K-edge XAS, VtC XES, and the accompanying DFT calculations provide significantly strong evidence for a low-spin d^8^ Cu(III) configuration on the Cu center of the [Cu(CF_3_)_4_]^−^ complex. This conclusion is in contradiction to recent studies of this complex, where it has been suggested that an “inverted ligand field” dictates that the MOs with majority Cu 3d character are fully populated, and a d^10^ electronic configuration is the most accurate description of the electronic structure (*vide infra*).^10^

The “inverted ligand field” phenomenon in Cu complexes was discussed in much further detail in a 2016 review by Hoffmann et al.,^17^ and in the pursuit of transparency, the authors stated that there remained no consensus as to what inverted ligand fields imply for the electronic structure in Cu complexes. We are of the opinion that increased covalency of the ligand and concomitant increase of ligand character in the valence MOs does not necessarily dictate the presence of an inverted ligand field. In classic molecular orbital theory, the energy difference between the d-orbitals and the ligand is used to describe the covalent character, where large separations are termed ionic and those approaching equal energy are highly covalent. However, the suggestion that the lowering of the d-orbital character in the LUMO below 50% yields an inverted ligand field is not a unique suggestion, only a renaming, and the covalency of the system would remain equal for d orbital/ligand orbital displacements equidistant from the 50% mark. For example, this exact case has been more simply described as stabilization (or destabilization) of the metal center through “inverted bonding”,^26^ as the ligand field of the metal center itself is not inverted.^1,61^ In metal complexes with such large amounts of metal–ligand covalency, referring to select few MOs with slightly larger Cu 3d admixtures as the Cu d-manifold is problematic, as the distribution of Cu character among the valence orbitals is vast and significantly lower than that which is seen in Cu(I) systems.

In 2016, Lancaster and co-workers employed a combination of Cu K-edge XAS and Cu 1s–2p resonant inelastic X-ray scattering (RIXS) in order to elucidate the electronic structure of the [Cu(CF_3_)_4_]^−^ complex.^14^ Significant attention was focused toward the 32% Cu 3d contribution to the LUMO (Cu 3d_*x*_^_2_^_–*y*_^_2_^–L σ*, b_2_ in *D*_2*d*_ symmetry), leading to the suggestion that the pre-edge transitions in the Cu K-edge XAS spectrum should be described as excitations of the Cu 1s core electrons to predominantly ligand MOs rather than to Cu 3d orbitals, implying a 3d^10^ electronic configuration and “inversion” of the ligand field on Cu.^14^ It would later be suggested that all Cu(III) systems exhibit inverted ligand fields and should be described physically as d^10^ systems, with the exception of the [CuF_6_]^3–^ species.^10^

In 2015, Tomson et al. investigated the Cu K-edges of a range of covalent β-diketiminate Cu complexes with co-ligands exhibiting various degrees of π acidity.^18^ In this work, pre-edge features with energies typical of Cu(III) species were found to be accompanied by uncharacteristically low energy rising edges, specifically Cu 1s → 4p excitations, something which was also subsequently observed in bipyridine coordinated Cu(I/II) complexes.^62^ Here the authors stress that an analysis based solely on the pre-edge energies of these complexes would have led to a Cu(III) assignment on many complexes that were shown via quantum chemical calculations to have ground states dominated by Cu(I)/Cu(II) configurations. Hence, this study emphasizes the importance of examining both the pre-edge and rising edge energy trends in the evaluation of the electronic structure of copper complexes. Unlike the low valent copper complexes studied by Tomson et al.,^18^ which exhibited higher energy pre-edges arising from Cu → π* transitions and low energy rising edge energies, the formally Cu(III) complexes investigated in the present study exhibit *both* higher energy pre-edge features (relative to the Cu(I) and Cu(II) species) and also higher energy rising edge features. Finally, this trend is extended to the VtC XES, where the Cu(III) systems exhibit higher energy Kβ_2,5_ features relative to the Cu(I)/(II) systems which supports a larger Z_eff_ on the three formally Cu(III) complexes. This reinforces the need to employ a more holistic approach to the investigation and evaluation of the electronic structures of highly covalent Cu complexes. It is here where the combination of the Cu K-edge XAS and Cu Kβ VtC XES can be combined to overcome limitations in assignment of oxidation state from XAS alone.

The stepwise increase in VtC emission energy with increased formal oxidation state in the [Cu^*n*+^(NHC_2_)]^*n+*^ series has displayed a clear sensitivity and utility of the VtC XES technique to the physical oxidation state of Cu. In combination with DFT calculations, the parent valence transitions of this series reflect the energy of the core 1s orbital and the effective nuclear charge (i.e., oxidation state). DFT calculations also identified the remarkably similar electronic structures of the [Cu(CF_3_)_4_]^−^ and [Cu^III^(NHC_4_)]^3+^ complexes: both complexes exhibit Cu 1s orbital ionization energies entirely consistent with that observed for [Cu^III^(NHC_2_)]^3+^, while also displaying strikingly similar valence MOs to one another, leading to their intense and high energy Kβ_2,5_ emission features. By simple analogy, one is led to conclude that [Cu(CF_3_)_4_]^−^ is in fact best described as a d^8^ Cu(III) center and that the d^10^ Cu(I) oxidation is not supported.

### Importance of Observables in Physical Oxidation State Assignment

The [CuCl_4_]^2–^ ion is often used as a reference point for ligand field inversion, due to the extensive amount of experimental data available. The spin population on Cu in the *D*_4*h*_ [CuCl_4_]^2–^ ion has been experimentally determined as 56–67% via a range of methods.^63^ Thus, it is accepted that the resulting ligand field is conventional, with the relative energies of the Cu frontier orbitals greater than those of the ligand(s). In order to computationally reproduce a >50% Cu d-orbital admixture into the HOMO of the [CuCl_4_]^2–^ complex via DFT methods, the amount of Hartree–Fock (HF) exchange that is employed by the selected functional must be calibrated.^63,64^ Analysis of the LUMOs (i.e., the α-spin lowest unoccupied MO in a spin-unrestricted description) of the Cu(III) systems investigated in the present study shows that there is diminished Cu *n*d admixture (Table S10), dropping below the 50% threshold frequently employed as the cutoff limit for this select definition of an “inverted ligand field”. However, this is also the case for the HOMO (i.e., the α-spin highest occupied MO in a spin-unrestricted description) of the Cu(II) center in [CuCl_4_]^2–^ analyzed by both Löwdin and Mulliken population analysis (B3LYP/def2-TZVP). In the [Cu^*n*+^(NHC_2_)]^*n+*^ redox series, the Cu(I) species has a HOMO with 58.3% Cu *n*d admixture (Löwdin), decreasing to 29.5% in the HOMO of the Cu(II) species, and then increasing again to 42.0% in the LUMO of the Cu(III) species. These MOs are effectively the analogous Cu 3d_*x*_^_2_^_–y_^_2_^–2p_x__,__y_ σ* antibonding orbitals across the redox series.

Despite the <50% Cu 3d admixture into the HOMO in [Cu^II^(NHC_2_)]^2+^, the Löwdin spin population on the Cu atom is ∼61.5%. Similar observations are made when observing the Löwdin population of the HOMO of *D*_4*h*_ [CuCl_4_]^2–^, which is calculated to have only 20.2% Cu *n*d admixture using the same B3LYP/def2-TZVP method. Comparatively, the calculated Löwdin population of the unrestricted natural orbitals (UNOs) gave a Cu *n*d admixture that was much closer to the calculated spin population (Table S10). This is also true for the Cu(II) complex [Cu^II^(NHC_2_)]^2+^, which is calculated to have a Cu Löwdin spin population of 61.5% and 61.0% Cu *n*d admixture in the HOMO from Löwdin population analysis of the UNOs. This is in stark contrast to the 29.5% Cu *n*d admixture in the HOMO according to the Löwdin population analysis of the canonical orbitals, which would qualify as an “inverted ligand field”, despite experimental EPR evidence for a significantly large Cu hyperfine and thus Cu admixture into the ground state wave function.^32^ In the case of the closed-shell Cu(I) and Cu(III) complexes, Cu *n*d admixtures in the HOMO and LUMO, respectively, according to the population analyses on the UNOs, are consistently much less than 10%, which is unrealistic in the case of the Cu(I) complex [Cu^I^(NHC_2_)]^+^ considering the HOMO has dominant Cu 3d_*x*_^_2_^_–*y*_^_2_^ character (Figure S24). Inspection of the quasi-restricted orbitals (QROs) shows that both in the Löwdin and Mulliken population analyses, the Cu *n*d admixture into the HOMO/LUMO for the closed shell Cu(I)/Cu(III) complexes closely match that of the canonical orbitals, whereas in the open shell Cu(II) complexes, the QROs are a much closer match to the UNOs than they are to the canonical orbitals. The wide variation in the degree of Cu admixtures suggests that population analyses of the MOs should be cautiously implemented when defining the electronic structures of Cu complexes, especially as there may be large disparities between the spectroscopically observable trends and the mathematically constructed MO populations.

The discrepancies between the values produced by various population analyses clearly highlight the crucial role of assessing the spectroscopic signatures from core X-ray spectroscopies when assigning oxidation states in high valent organocopper species. The Cu(III) assignment for [Cu(CF_3_)_4_]^−^ is entirely consistent among all of the spectroscopic observables for this complex to date. Here, it is demonstrated how the observed transitions for the Cu K-edge XAS and VtC XES of [Cu(CF_3_)_4_]^−^ are due to a large Z_eff_, consistent with a Cu(III) oxidation state.

The low intensity of the Cu L-edges in [Cu(CF_3_)_4_]^−^, derived from 1s–2p RIXS experiments, have been interpreted as a diminished d-hole character, leading to invocation of an inverted ligand field and a Cu(I) d^10^ configuration. However, the energies of the observed L-edge transitions in [Cu(CF_3_)_4_]^−^ are in agreement with numerous other formally assigned Cu(III) complexes.^9,10,28^ While the L-edges of [Cu(CF_3_)_4_]^−^ and other Cu(III) centers do indeed exhibit decreased intensities per d-hole, this is clearly correlated to the increased ligand covalency, as probed by ligand K-edge XAS.^28^ This interpretation of the low Cu d-character measured by the L-edge XAS is also consistent with *S* = 1/2 Cu(II) thiolate centers such as plastocyanin, which exhibits typical blue-copper EPR signatures and ∼41% Cu 3d admixture into the ground state wave function,^31^ being described as highly covalent copper-based paramagnetic centers with large amounts of ligand character contributing to the SOMO, as opposed to Cu(I) systems with ligand-based radical(s), as would be the required description by employing this interpretation of an inverted ligand field.

Furthermore, the lack of classic d–d transitions in the UV–vis spectrum of [Cu(CF_3_)_4_]^–^ is expected for the large ligand field expansion in the presence of the high valent copper ion, and its increased Z_eff_ (Figure 6). From the combination of VtC XES and XAS studies as well as computational investigations, it is demonstrated that the MOs responsible for the dominant VtC feature and pre-edge transition in [Cu(CF_3_)_4_]^−^ are the ligand-based HOMO/HOMO–1 and the Cu 3d_*x*_^_2_^_–__y_^_2_^ localized LUMO, respectively. Therefore, the combination of VtC XES and Cu K-edge XAS is a direct measure of the HOMO–LUMO gap for [Cu(CF_3_)_4_]^−^. Electronic excitations between the HOMO and LUMO are calculated by TDDFT (Figure S29) to have negligible oscillator strength and are thus not observed in the reported UV–vis spectrum.^14^ Due to the large ligand field, the lowest energy d–d transition with non-negligible oscillator strength is calculated at ∼36,600 cm^–1^ and is one with contributions from two valence MOs with significant Cu 3d_*z*^2^_ character, forming a d_*z*^2^_ → d_*x*_^_2_^_–*y*_^_2_^ (LUMO) transition (Figure S29). A pair of much more intense transitions occur within the same absorption band, stemming from a combination of excitations from different donor orbitals into the LUMO: the ligand based *e* doublet (C–C σ bonding, also responsible for the VtC XES intensity) and the 3d_*xz*/__yz_*e* doublet. To even higher energy is a yet more intense absorption band involving identical orbital sets. The involvement of the C–C σ-bonded ligand MOs in these electronic excitations provides significant charge transfer character to the transitions; thus, the intensity is much larger than other d–d transitions mentioned (Figure S29). A remarkably similar pair of intense absorption bands is observed in the [Cu^III^(NHC_4_)]^3+^ complex (Figure S30), as expected from the similarities in electronic structures. The highest energy absorption band shown in Figure S30 is isoenergetic between both the [Cu(CF_3_)_4_]^−^ and [Cu^III^(NHC_4_)]^3+^ complexes, and stems from highly similar donor MO sets into the largely 3d_*x*_^_2_^_–*y*_^_2_^ LUMO. This demonstrates how the high valent and highly covalent Cu(III) complexes with C_4_ donor sets exhibit atypical high energy and high intensity pseudo d–d transitions due to the increased charge transfer character that is imparted by the involvement of σ-type MOs formed by adjacent carbon atoms in the first coordination sphere.

## Conclusions

A combination of Cu K-edge XAS and Cu Kβ VtC XES has been used to spectroscopically probe the oxidation state in a suite of organometallic Cu complexes and has provided strong evidence for the presence of a low-spin d^8^ Cu(III) center in three separate ligand systems. The pre-edge, edge, and white lines of the complexes studied exhibit stepwise increases in energy as the physical oxidation state of the Cu center is increased, which is supported by TDDFT calculations. Similarly, both the energies and intensities of the dominant Kβ_2,5_ features in the VtC XES increase in a predictable fashion upon Cu oxidation and are well reproduced by DFT calculations. The most intense VtC features occur in the Cu(III) complexes, with the more symmetric pseudo-*D*_4*h*_ [Cu^III^(NHC_4_)]^3+^ and *D*_2*d*_ [Cu(CF_3_)_4_]^−^ species exhibiting the largest intensity—the result of increased Cu *n*p character being imparted into the valence MOs in these highly covalent C_4_ coordination environments. The Kβ_2,5_ intensity therefore clearly scales with the σ-donor strength of the ligands; however, there is also an angular dependence on this intensity in the macrocyclic NHC-based complexes, where the intensity increases as the *trans* C–Cu–C angles approach linearity.

Analysis of the valence MOs shows that the Cu contributions to the antibonding 3d_*x*_^_2_^_–*y*_^_2_^-L orbital in both Cu(II) (HOMO) and Cu(III) species (LUMO) is significantly less than the 50% threshold that is often used to describe the limit between regular and inverted ligand fields. Analysis of the HOMO in the ubiquitous [CuCl_4_]^2–^ standard shows that despite having ∼60% spin population on Cu atom, the calculated Cu 3d admixture in the highest occupied α spin MO is lower than all of the complexes investigated in the present study. Although MO population analysis is demonstrated to be able to explain the observable trends in the experimental XAS and VtC XES data, the values extracted from this mathematical procedure can vary dramatically based on the type of orbital localization method and should therefore be used qualitatively rather than quantitatively.^65,66^ The results of the XAS and VtC XES studies, in combination with (TD)DFT calculations, are in strong support of assignment of the formally Cu(III) systems as low-spin d^8^ ions, characterized by highly covalent bonding networks and high energy pre-edge and rising edge Cu K-edge XAS and VtC features.

In summary, we have shown that by combining Cu K-edge XAS, VtC XES and computational methods, a more complete description of the electronic structure of high-valent copper complexes is achieved. Herein, the controversial [Cu(CF_3_)_4_]^−^ ion is shown to be best described as a low-spin d^8^ system with a d_*x*_^_2_^_–*y*_^_2_^ LUMO that is engaged in highly covalent bonding interactions with the homoleptic (CF_3_^–^)_4_ ligand donor set. Finally, we emphasize the need to take a comprehensive approach when defining the electronic structure of highly covalent copper complexes. Careful evaluation of the spectroscopic transition energies and intensities, preferably utilizing more than one experimental method, is needed to arrive at a more comprehensive understanding

## Supporting Information Available

Materials and Methods; analytical characterization of [Cu^III^(NHC_4_)](PF_6_)_3_, additional XAS and XES, DFT computations, xyz coordinates (PDF)

## Accession Codes

CCDC 2104793 contains the supplementary crystallographic data for this paper. These data can be obtained free of charge via www.ccdc.cam.ac.uk/data_request/cif, or by emailing data_request@ccdc.cam.ac.uk, or by contacting The Cambridge Crystallographic Data Centre, 12 Union Road, Cambridge CB2 1EZ, UK; fax: +44 1223 336033.
