# Supplementary material for: Combining Valence-to-Core X-ray Emission and Cu K-edge X-ray Absorption Spectroscopies to Experimentally Assess Oxidation State in Organometallic Cu(I)/(II)/(III) Complexes
Source: J Am Chem Soc. 2022 Jan 20;144(6):2520–34. doi: 10.1021/jacs.1c09505 (PMC8855422; doi:10.1021/jacs.1c09505)
Supplement: Supplementary file 1 — ja1c09505_si_001.pdf [file ja1c09505_si_001.pdf]

# Supplementary Information

# Combining Valence-to-Core X-ray Emission and Cu K-edge X-ray Absorption Spectroscopies to Experimentally Assess Oxidation State in Organometallic Cu(I)/(II)/(III) Complexes

Blaise L. Geoghegan,[a,b] Yang Liu,[c] Sergey Peredkov,[a] Sebastian Dechert,[c] Franc Meyer,[c] Serena DeBeer,[a] George E. Cutsail III[a,b]*

[a] Max Planck Institute for Chemical Energy Conversion, Stiftstrasse 34–36, 45470 Mülheim an der Ruhr, Germany

[b] Institute of Inorganic Chemistry, University of Duisburg-Essen, Universitätsstrasse 5–7, 45117 Essen, Germany

[c] Institute of Inorganic Chemistry, University of Göttingen Tammannstrasse 4, 37077 Göttingen, Germany

email: george.cutsail@cec.mpg.de

# Contents


Contents ........ 2

Experimental ........ 3

- Sample Preparation and Synthesis ........ 3
- X-Ray Structure Determination ........ 4
- X-ray Absorption Data Collection ........ 4
- X-ray Emission Data Collection ........ 4
- Density Functional Theory ........ 5
- NMR Spectroscopy ........ 6
- UV-Vis and infrared spectroscopy ........ 13
- Mass Spectrometry ........ 14
- SQUID magnetometry ........ 14
- X-ray Crystallography ........ 16

X-ray Spectroscopy ........ 18

- X-ray Absorption ........ 18
- X-ray Emission ........ 20
  - Cu Kβ Mainlines ........ 20
  - Cu Kβ VtC XES ........ 21
- UV-Vis ........ 30
- Example Orca Input Files ........ 32
  - Geometry Optimization ........ 32
  - X-ray Absorption Spectroscopy ........ 32
  - X-ray Emission Spectroscopy ........ 32
  - Löwdin and Mulliken Population analysis of QROs ........ 33
  - Broken Symmetry ........ 33
  - UV-vis spectroscopy ........ 33
- XYZ Coordinates for Orca Calculations ........ 34

References ........ 40

# Experimental

## Sample Preparation and Synthesis

**General Considerations.** CuCl, $CuF_2$, $CuCl_2$ were purchased from Strem Chemicals and used as received. $CF_3SiMe_3$, and KF were purchased from Sigma-Aldrich (Germany) and used as received. $[Cu^{I}(NHC_2)](PF_6)$, $[Cu^{II}(NHC_2)](PF_6)_2\cdot CH_3CN$ and $[Cu^{III}(NHC_2)](PF_6)_2(SbF_6)\cdot CH_3CN$ were prepared as previously described.[1] $[NBu_4][Cu(CF_3)_4]$ was synthesized via the method described previously by A. M. Romine *et al.*[2] The tetraimidazolium salt calix[4](4,5-dimethylimidazolium) hexafluorophosphate ($[H_4L^{Me}](PF_6)_4$) was synthesized according to reported literature procedures.[3] $Me_3CN$-$d_3$ was dried with 3 Å molecular sieves and stored in the glovebox. All other chemicals and solvents were purchased from commercial suppliers, and used without further purification. All manipulations of air- and moisture-sensitive materials were performed under an atmosphere of dry dinitrogen with the rigorous exclusion of air and moisture using standard Schlenk techniques, or in a $N_2$-filled glovebox. $^{1}H$, $^{31}P$ and $^{19}F$ NMR spectra were recorded with Bruker 300 NMR spectrometer at 300 MHz, 122 MHz and 282 MHz, respectively. $^{13}C$ NMR spectra were recorded with Bruker 500 NMR spectrometer at 125 MHz. Variable-temperature NMR were recorded with Bruker 400 NMR spectrometer at 400 MHz. All NMR chemical shifts were reported in units of ppm with references to the residual protons of the deuterated solvents for proton chemical shifts (MeCN-$d_3$ $\delta$H = 1.94 ppm), the $^{13}C$ of deuterated solvents for carbon chemical shifts (MeCN-$d_3$ $\delta$C = 1.32, 118.36 ppm), the $^{19}F$ of $CFCl_3$ (external standard) for fluorine chemical shifts, and the $^{31}P$ of 85% phosphorous acid (external standard) for phosphine chemical shifts. ESI mass spectra were recorded on a Bruker HCT ultra spectrometer. Elemental analyses were performed by the analytical laboratory of the Institute of Inorganic Chemistry at the University of Göttingen using an Elementar Vario EL III instrument. UV-Vis spectra were recorded with an Agilent Cary 60. IR spectra were recorded inside a glovebox on a Cary 630 FTIR spectrometer equipped with a Diamond ATR module and analyzed by FTIR MicroLab software.

**Preparation of $[Cu(NHC_4)](PF_6)_3$.** This complex was synthesized via a procedure described in literature for the parent non-methylated complex,[4] with slight modifications. $[H_4L^{Me}](PF_6)_4$ (351 mg, 0.35 mmol) and copper(II) acetate monohydrate (138 mg, 0.69 mmol) were dissolved in 10 mL of reagent grade dimethyl sulfoxide in air. The blue reaction mixture was stirred at room temperature for 30 min and then stirred at 40 °C for 5 h. During this time the color of the solution changed from blue to clear green. The solution was cooled to room temperature and the crude product was precipitated with a large amount of dichloromethane (70 mL), forming a fine white precipitate. After filtration, the resulting white solid was consecutively washed with dichloromethane (3 mL × 3) and $Et_2O$ (5 mL × 3) and dried under vacuum to give **$[Cu(NHC_4)](PF_6)_3$** as a white powder (215 mg, 67% yield). Yellowish crystals suitable for X-ray diffraction studies were grown by diffusing $Et_2O$ into a saturated MeCN solution of the complex at room temperature. $^{1}H$ NMR (300 MHz, MeCN-$d_3$, 298 K): $\delta$ (ppm) 6.12 (s, 8H, C*H*$_2$-linkers), 2.49 (s, 24H, -C*H*$_3$). $^{13}C$ NMR (125 MHz, MeCN-$d_3$, 298 K): $\delta$ (ppm) 149.5 (N*C*N), 129.7 (*C*-imidazolyl backbone), 59.9 (*C*H$_2$-linkers), 9.32 (-*C*H$_3$). $^{19}F$ NMR (282 MHz, MeCN-$d_3$, 298 K): $\delta$ (ppm) -72.9 (*d*, $J_{P\text{-}F}$ = 707.2 Hz). $^{31}P$ NMR (122 MHz, MeCN-$d_3$, 298 K): $\delta$ (ppm) -144.7 (hept, $J_{F\text{-}P}$ = 707.2 Hz). Anal. Calcd. for $CuC_{24}H_{32}N_8P_3F_{18}$: C 30.96, H 3.46, N 12.04; Found: C 30.72, H 3.38, N 11.96. ESI–MS (MeCN) m/z (%): 785.1 (100) $[M\text{-}PF_6]^+$. Absorption spectrum (in MeCN): $\lambda_{max}$, nm ($\varepsilon$, $M^{-1}$ $cm^{-1}$) 230 (27200), 260 (34200), 347 (1740). ATR-IR (powder, $cm^{-1}$): $v$ = 1641 (w), 1496 (m), 1440 (w), 1405 (w), 1381 (m), 1266 (w), 1199 (w), 1044 (w), 944 (w), 921 (w), 822 (s), 739 (m), 711 (w), 649 (w), 555 (s), 531 (m), 519 (w).

**X-ray spectroscopy sample preparation.** For XAS experiments, samples were diluted with boron nitride to achieve a 2% (w/w) concentration of copper, then packed into 1 mm thick aluminum sample cells and sealed with 38 μm Kapton tape. For XES experiments, all samples were measured in the solid state at room temperature. The pure solids were ground to a fine powder and packed into 0.5 mm thick aluminum sample holders and sealed with 38 μm Kapton tape.

## X-Ray Structure Determination

Crystal data and details of the data collections for $[Cu(NHC_4)](PF_6)_3$ are given in Table S2, the molecular structure of the cation $[Cu^{III}(NHC_4)]^{3+}$ is shown in Figure S16. X-ray data were collected on a BRUKER D8-QUEST diffractometer (monochromated Mo-Kα radiation, λ = 0.71073 Å) by use of ω and ϕ scans at low temperature. The structure was solved with SHELXT and refined on $F^2$ using all reflections with SHELXL-2018.[5] Non-hydrogen atoms were refined anisotropically. Hydrogen atoms were placed in calculated positions and assigned to an isotropic displacement parameter of 1.5/1.2 $U_{eq}$(C). Four F-atoms of one $PF_6$– counterion were found to be disordered (occupancy factors 0.48(3)/0.52(3)). SADI ($d_{F\cdots F}$) restrain were applied to model the disordered parts. Absorption correction was performed by the multi-scan method with SADABS.[6] CCDC 2104793 contains the supplementary crystallographic data for this paper. These data can be obtained free of charge from the Cambridge Crystallographic Data Centre via www.ccdc.cam.ac.uk/structures.

## X-ray Absorption Data Collection

Data collection procedures for samples of $[Cu^{n+}(NHC_2)]^{n+}$ complexes were previously described[1] albeit, only the Kβ high-energy resolution fluorescence detected (HERFD) XAS was reported. Simultaneous transmission data was also collected at the Stanford Synchrotron Radiation Lightsource (SSRL), BL6-2, and is now reported here. Comparisons of the Kβ-HERFD and transmission XAS are shown in Figure S17. $[Cu^{III}(NHC_4)]^{3+}$ and $[Cu^{III}(CF_3)_4]^-$ were measured in transmission mode at room temperature and ambient conditions at the SAMBA beamline of the SOLEIL synchrotron under ring conditions of 3 GeV and 500 mA. Incident X-ray radiation was monochromatized by using a Si(220) sagittal focusing monochromator and calibrated by using a Cu foil calibration standard and assigning the energy of the first inflection point to 8980.3 eV. Five scans were measured and averaged using in-house developed MATLAB R2019b scripts and all further data processing was conducted using the Athena program for x-ray spectroscopic data processing.[7] The background was accounted for by fitting a polynomial to the pre-edge region and subtracting this polynomial from the entire spectrum. All spectra were normalized by fitting a flattened polynomial to the post-edge region of the spectra and normalizing the edge jump to 1.0. No spectral changes due to photodamage were observed after ten 65 second scans on a single sample spot (beam size = 2000 x 300 μm {width x height}).

## X-ray Emission Data Collection

XES data collection was done at the PINK tender X-ray beamline at BESSY II. The PINK beamline is currently being operated in commissioning mode. A considerable gain in fluorescence signal could be obtained by using a multilayer monochromator (~100 eV band pass), and the energy of the incident beam was 9.5 keV. The beam size on the sample was 30 μm × 500 μm (V×H). All spectra were collected using an in-house-designed energy-dispersive von Hamos spectrometer with a vacuum sample chamber environment (5 mbar working pressure).

The analyser was set up in a vertical dispersion direction, taking advantage of the small vertical beam size to improve the energy resolution. A Si(444) crystal with a bending radius of R=250mm dispersed incoming fluorescence radiation onto an Eiger detector with a 75 µm × 75 µm pixel size. The 80mm x 40mm detector chip accepted fluorescent radiation reflected from the crystal analyser under 68º-60.7º brag angles that corresponded to an energy window of 8530-9065 eV. Calibration of the energy scale was achieved by measuring reference X-ray emission lines of anhydrous CuCl in the same instrumental configuration and setting the energy of the $K\beta_{1,3}$ mainline feature to 8904.7 eV and VtC feature to 8977.4 eV (Figure S1). We have previously collected the Kβ emission of anhydrous CuCl at both ID26, ESRF, Grenoble, France, Figure 20. The spectrometer at ID26 was calibrated by scanning of elastic scattering lines through the available emission range of the spectrometer. The monochromator for each was calibrated by setting the first inflection point of a Cu foil to 8980.3 eV.

Cu Kβ XES damage scans and assessments were performed for all samples. In all cases, the data were collected in continuous motion. Samples were scanned at a rate of 100 µm/s, resulting in a total exposure time of 0.50 s/pass and the total exposure was between 6 and 10 s per sample. None of the samples exhibited damage or changes in the Kβ emission spectra within the measurement scan periods. XES data were processed and fit using in-house developed MATLAB scripts and the integrated intensity of the Kβ main line was set to 1.0.

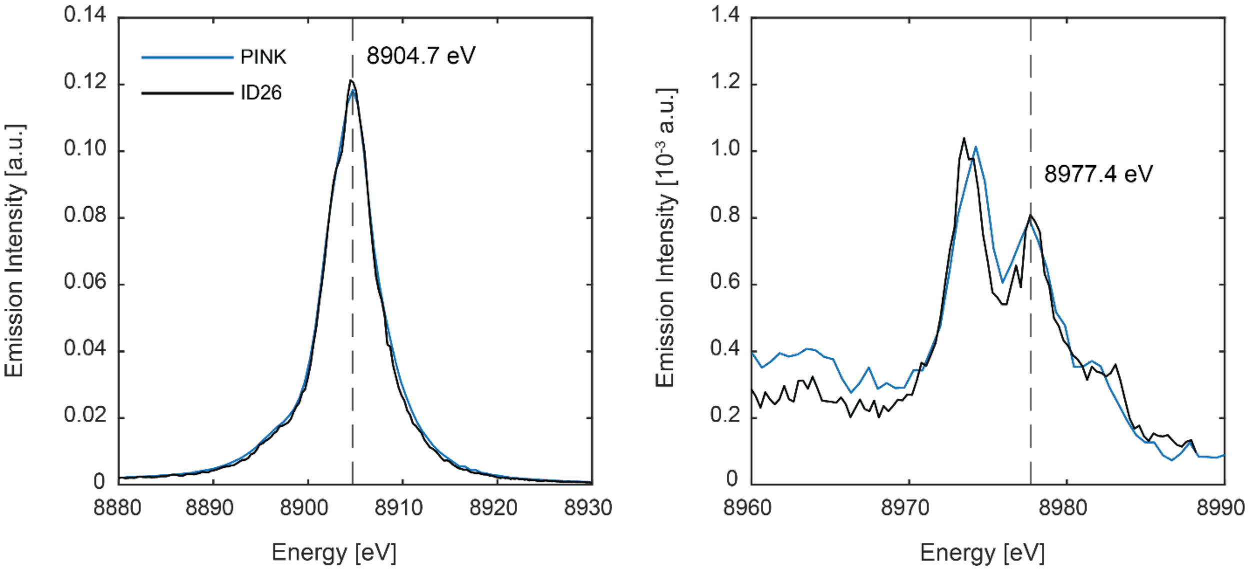


**Figure S1** Cu Kβ mainline (left) and VtC region (right) for solid CuCl energy calibrants as measured at the ID26 beamline at ESRF, Grenoble, and the PINK beamline at BESSY II, Berlin. The spectra were normalized by setting the area of the mainline region (8860-8930 eV) to 1.0 a.u.

## Density Functional Theory

All DFT and TDDFT were performed with the ORCA electronic structure package 4.2.[8,9] Geometry optimization calculations were carried out at the B3LYP[10,11] level of theory, using def2- variants of Ahlrichs' all-electron Gaussian triple-ζ valence polarized recontracted basis set (def2-TZVP)[12] on all atoms and the AutoAux basis option for ORCA.[13] The calculations employed the resolution of identity (RI-J) algorithm for the computation of the Coulomb terms and the 'chain of spheres exchange' (COSX) algorithm for the calculation of the exchange terms[14] and a tight self-consistent field (SCF) convergence threshold was chosen via the "TightSCF" keyword. An increased grid was used during the SCF iterations (Grid4), and for the final energy evaluation after SCF

convergence Grid7 was used. The conductor-like polarizable continuum model (CPCM) was used for charge compensation in all calculations of complexes carrying a net positive/negative charge.[15] Vibrational frequencies were calculated for all optimized structures and the absence of imaginary modes alongside six 0 $cm^{-1}$ modes confirmed that the true minima were obtained in all cases.

Cu Kβ VtC XES were calculated using the features described above, with the exception that a scalar relativistic basis set (ZORA-def2-TZVP)[12] was employed. In the XES block, "CoreOrb" dictates the orbitals that will be the electron acceptors. For transition metal XES spectra, the metal 1s orbital is usually orbital 0, and is thus selected. OrbOp defines the operators of the electrons (α = 0, β = 1) that will be calculated, both of which were selected. CoreOrbSOC defines which core orbitals (α = 0, β = 1) are treated with spin-orbit coupling (SOC), both of which were selected to accommodate the OrbOp selection. A 2.0 eV full-width half-max Gaussian broadening and -5.1 eV energy shift was applied when plotting all calculated XES spectra.

Time-dependent DFT (TD-DFT) calculations used the B3LYP functional, ZORA-def2-TZVP basis set, AutoAux and the zeroth-order regular approximation (ZORA) to account for scalar relativistic effects.[16,17] TDDFT calculated XAS were shifted by -3.1 eV and a 2.5 eV full-width half-max Gaussian broadening was applied when plotting all calculated XES spectra. UV-vis spectra were broadened by 2,500 $cm^{-1}$.

## NMR Spectroscopy

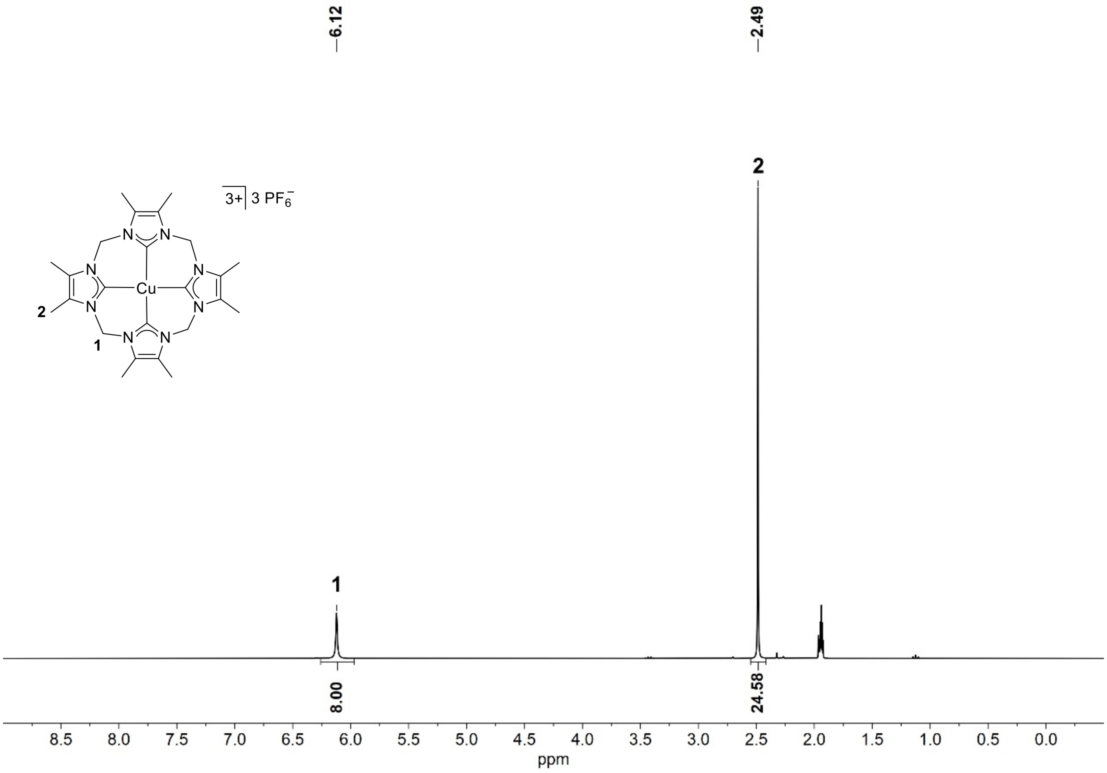


**Figure S2** $^{1}$H NMR spectrum of **$[Cu(NHC_4)](PF_6)_3$** in MeCN-$d_3$ (300 MHz, 298 K).

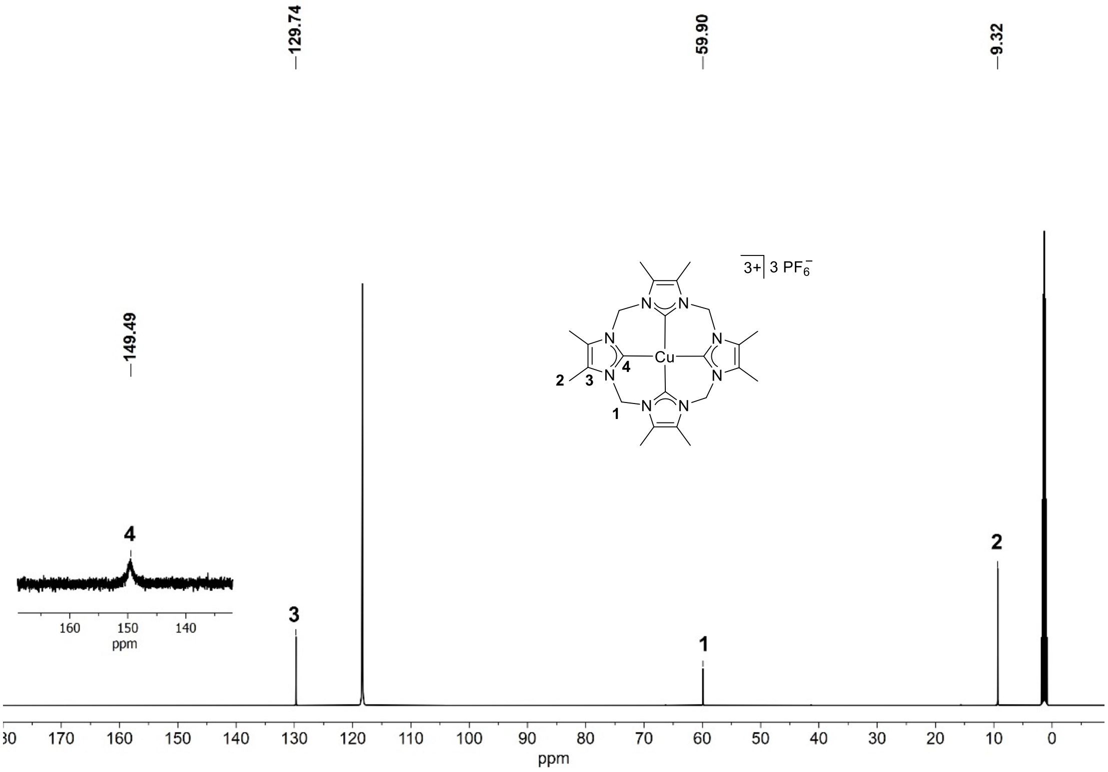


**Figure S3** $^{13}$C NMR spectrum of **[Cu(NHC$_4$)](PF$_6$)$_3$** in MeCN-$d_3$ (125 MHz, 298 K).

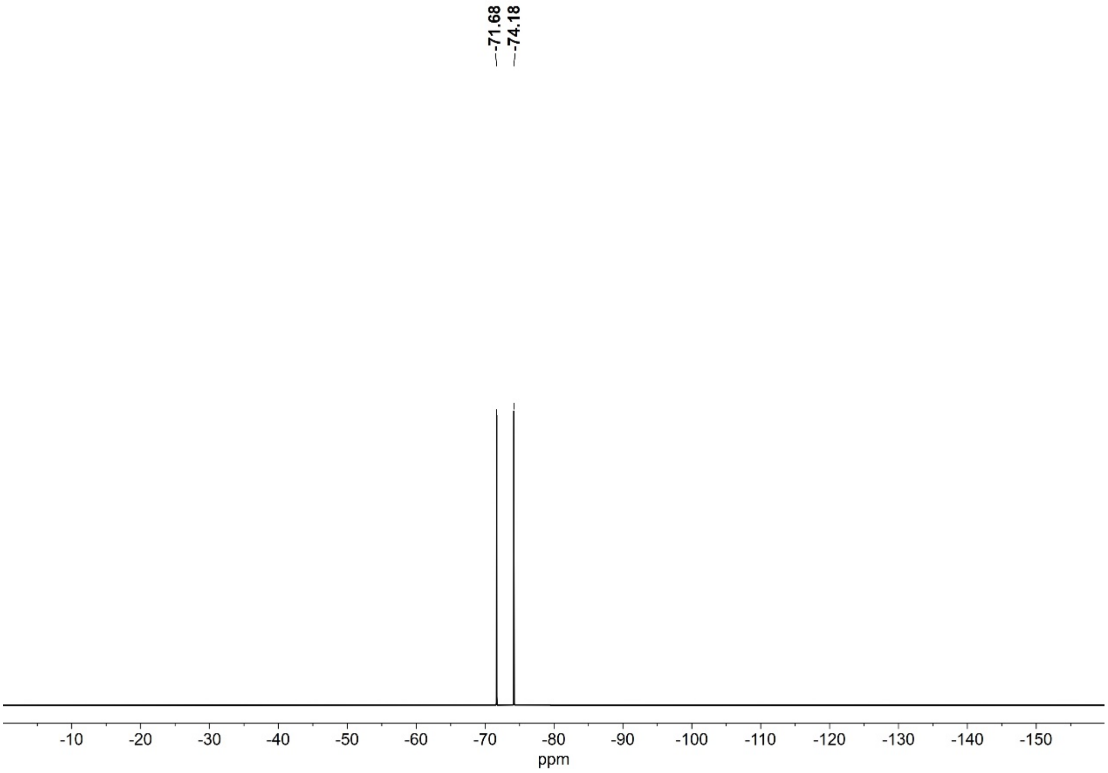


**Figure S4** $^{19}$F NMR spectrum of **[Cu(NHC$_4$)](PF$_6$)$_3$** in MeCN-$d_3$ (282 MHz, 298 K).

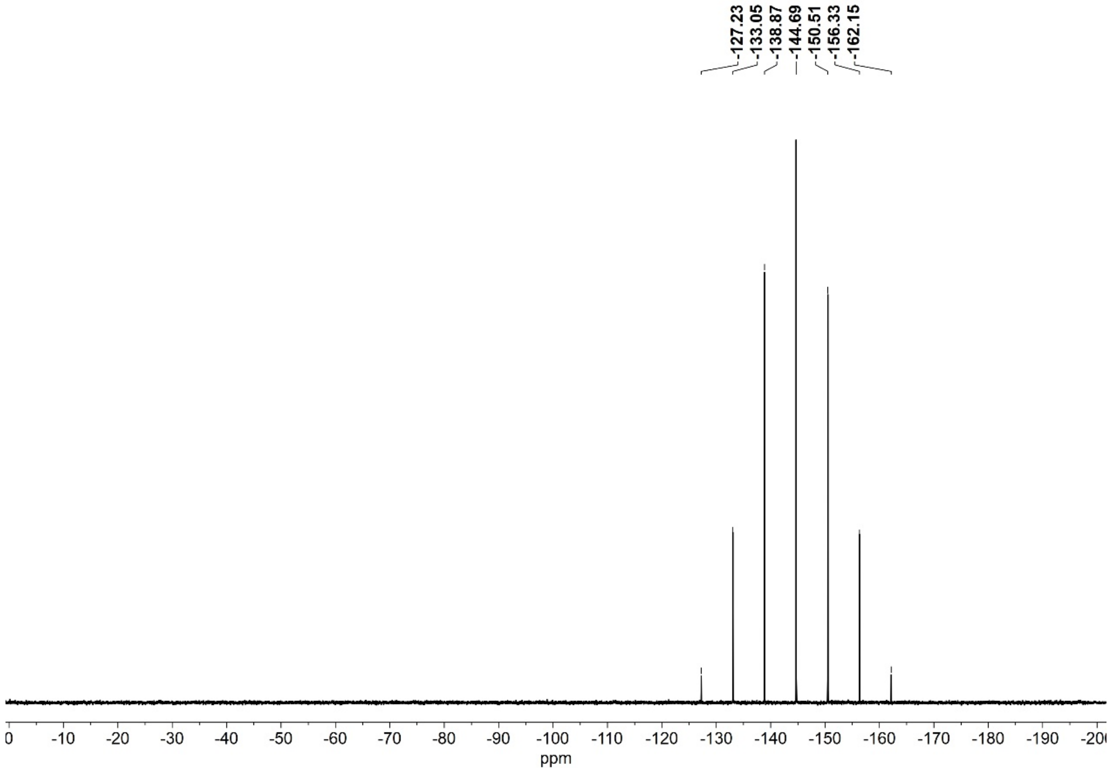


**Figure S5** $^{31}$P NMR spectrum of **[Cu(NHC$_4$)](PF$_6$)$_3$** in MeCN-$d_3$ (122 MHz, 298 K).

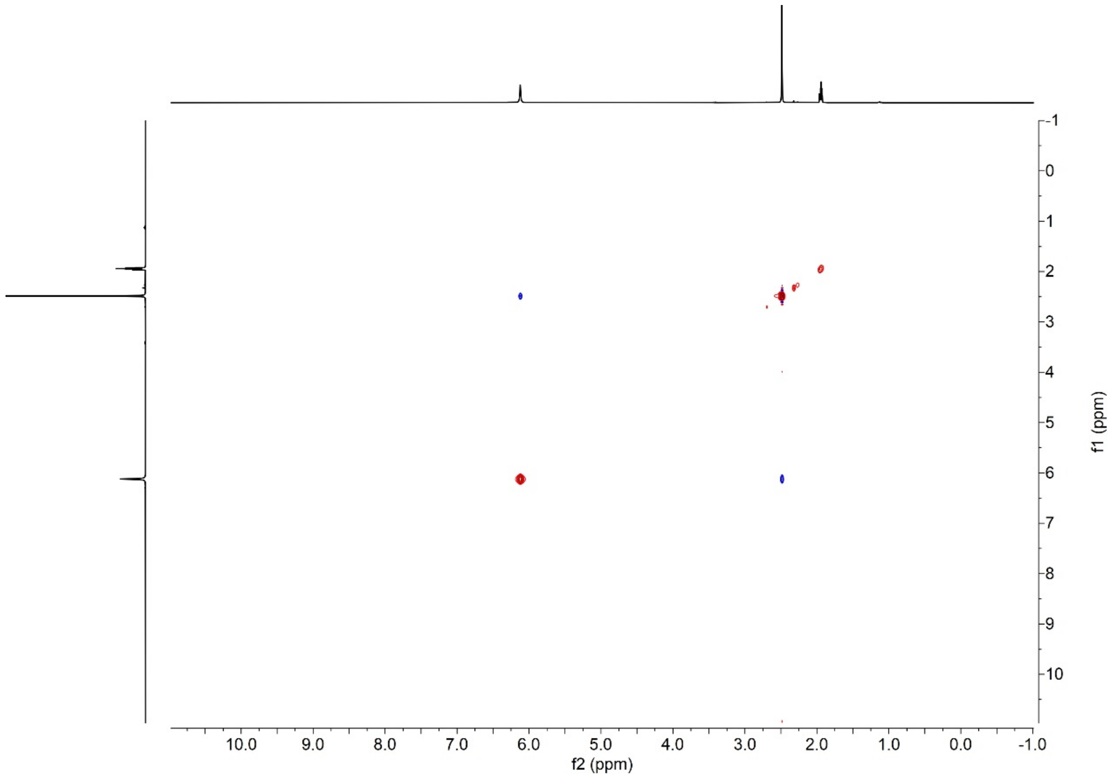


**Figure S6** $^1$H-$^1$H NOESY spectrum of **[Cu(NHC$_4$)](PF$_6$)$_3$** in MeCN-$d_3$ (300, 300 MHz, 298 K).

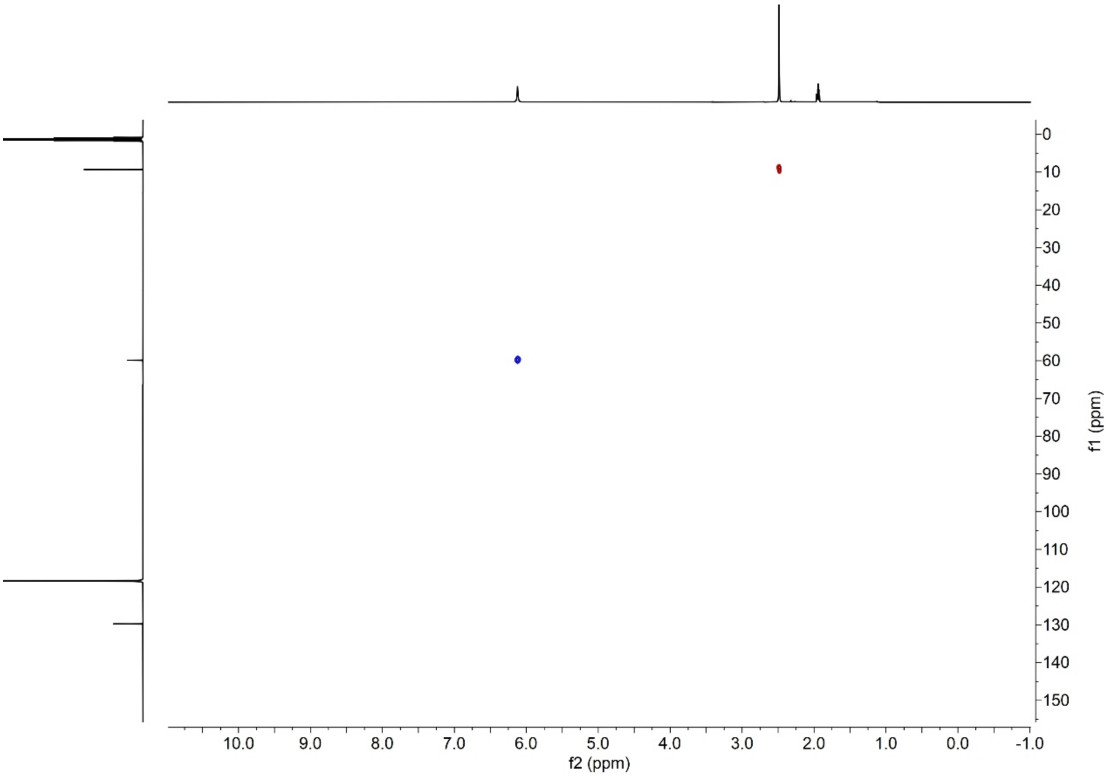


**Figure S7** $^{1}H$-$^{13}C$ HSQC spectrum of **[Cu(NHC$_4$)](PF$_6$)$_3$** in MeCN-$d_3$ (300, 75 MHz, 298 K).

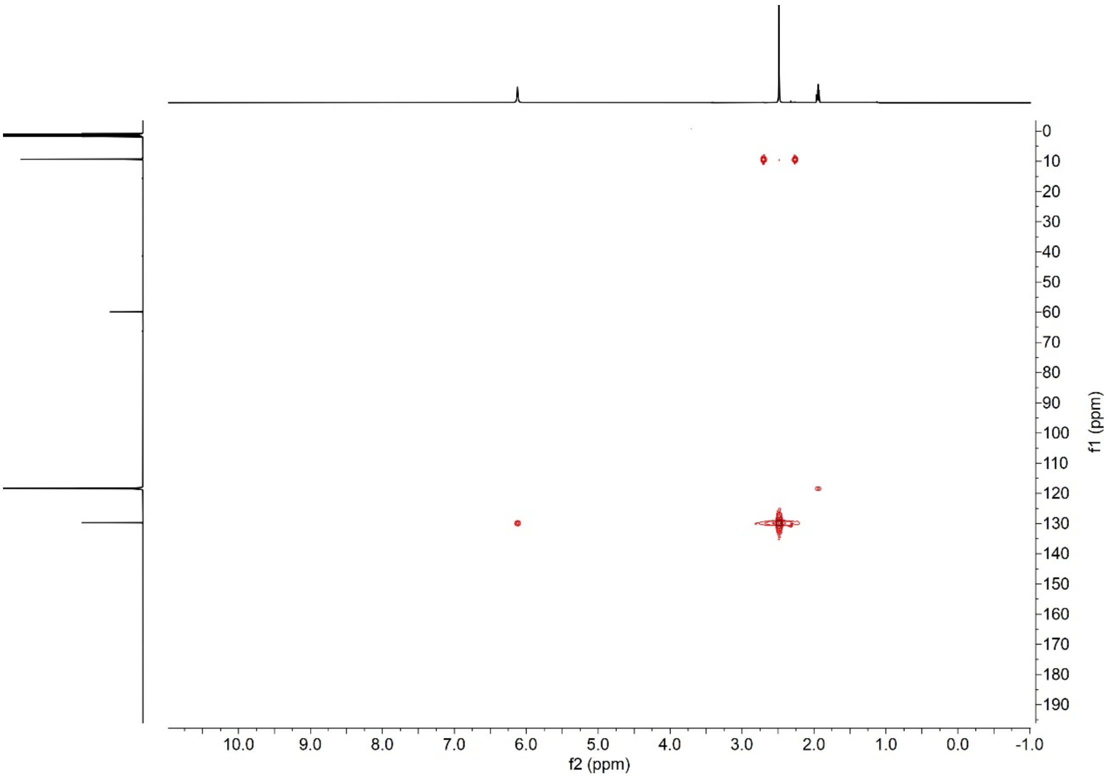


**Figure S8** $^{1}H$-$^{13}C$ HMBC spectrum of **[Cu(NHC$_4$)](PF$_6$)$_3$** in MeCN-$d_3$ (300, 75 MHz, 298 K).

### VT-NMR and Dynamic Behavior

At room temperature, the $^{1}$H NMR spectrum of **[Cu(NHC$_4$)](PF$_6$)$_3$** in MeCN-$d_3$ shows two sharp singlets at 6.12 ppm ($CH_2$ linkers) and 2.49 ppm ($CH_3$) (Figure S9). Upon decreasing the temperature, signal broadening followed by splitting of the peak at 6.12 ppm ($CH_2$ linkers) into a pair of apparent doublets (AB system, $J_{ab}$ = 13.6 Hz) is observed (Figures S8). This suggests that the dynamic behavior (conformational inversion of the macrocyclic ligand) becomes slow on the NMR time scale at low temperatures (coalescence temperature $T_c$ = 270 K, $\Delta G^{\ddagger}$ = 12.7 kcal·mol$^{-1}$). Further analyses of the VT-NMR data yielded the activation energy ($E_a$ = 18.3 kcal·mol$^{-1}$) as well as activation parameters ($\Delta H^{\ddagger}$ = 17.8 kcal·mol$^{-1}$ and $\Delta S^{\ddagger}$ = 19.0 cal·mol$^{-1}$·K$^{-1}$) from Arrhenius and Eyring plots, respectively (Table S1, Figures S9 and S10).

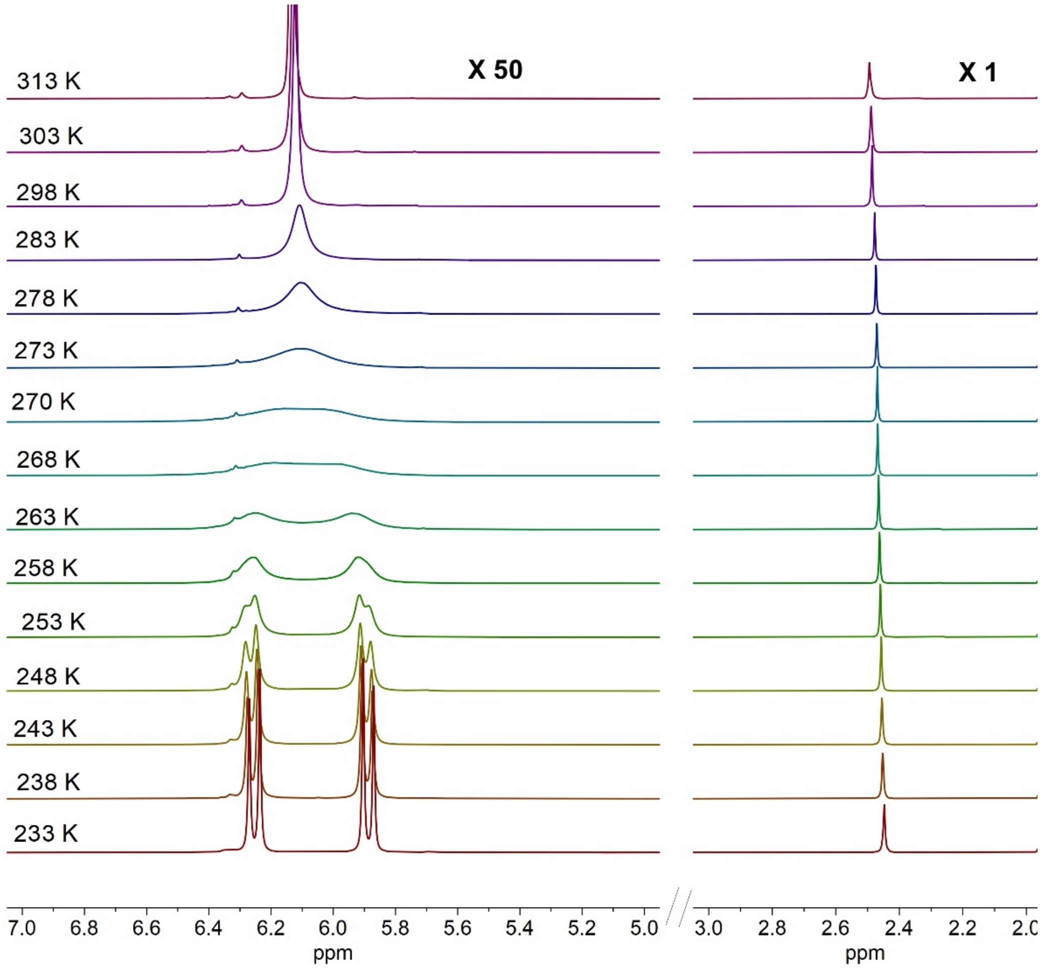


**Figure S9** Variable-temperature $^{1}$H NMR spectra of **[Cu(NHC$_4$)](PF$_6$)$_3$** in MeCN-$d_3$ (400 MHz, temperature range from 233 K to 313 K). Only regions 5.0–7.0 ppm (the intensity was scaled up by a factor of 50) and 2.0–3.0 ppm are shown for clarity.

To determine these activation parameters for the ring inversion of **[Cu(NHC$_4$)](PF$_6$)$_3$**, the rate constants were calculated according to the formulas in literature.[18] The line width at half height was determined by using the line fitting function of MestReNova program. Ring inversion was assumed to be frozen at 233 K in this case. The Gibbs free energy of activation $\Delta^{\ddagger}$G at coalescence temperature $T_c$ can be calculated from equation (1). The calculation of thermodynamic parameters (activation energy, enthalpy and entropy) was done according to the Arrhenius equation (2) and Eyring equation (3).

$$\Delta G^{\ddagger} = RT_c\left(In\frac{T_c}{k_c} + In\frac{k_B}{h}\right) \qquad (1)$$

$$In\, k = -\frac{E_a}{R}\frac{1}{T} + In(A) \qquad (2)$$

$$In\frac{k}{T} = -\frac{\Delta H^{\ddagger}}{R}\frac{1}{T} + \frac{\Delta S^{\ddagger}}{R} + In\frac{k_B}{h} \qquad (3)$$

**Table S1** Calculated values for *k* at different temperatures *T*.

| *T* (K) | *k* ($s^{-1}$) | ln*k* ($s^{-1}$) | ln(*k*/T) ($s^{-1}$ $K^{-1}$) |
|---|---|---|---|
| 238 | 3.2 | 1.155 | -4.318 |
| 243 | 7.8 | 2.057 | -3.436 |
| 248 | 16.1 | 2.780 | -2.734 |
| 253 | 32.2 | 3.472 | -2.061 |
| 258 | 78.2 | 4.360 | -1.193 |
| 263 | 162.8 | 5.092 | -0.480 |
| 268 | 272.4 | 5.607 | 0.016 |
| 270 | 323.8 | 5.780 | 0.182 |
| 273 | 424.4 | 6.051 | 0.441 |
| 278 | 806.2 | 6.692 | 1.065 |
| 283 | 1733.0 | 7.458 | 1.812 |
| 298 | 8948.4 | 9.099 | 3.402 |
| 303 | 12937.1 | 9.468 | 3.754 |

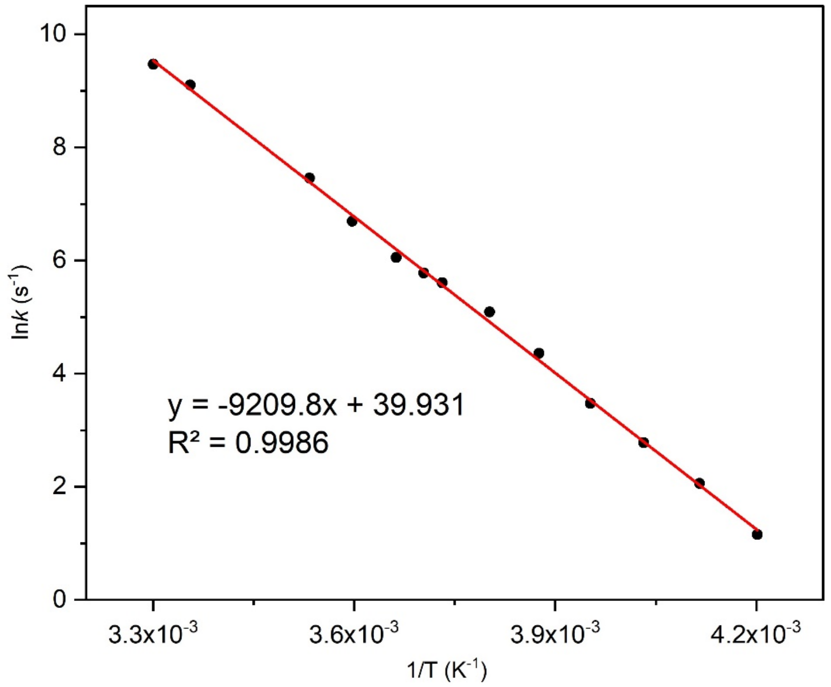


**Figure S10** Arrhenius plot for the determination of $E_a$ for the ring inversion in **[Cu(NHC$_4$)](PF$_6$)$_3$**. The following value was determined: $E_a$ = 18.3 ± 0.2 kcal·mol$^{-1}$.

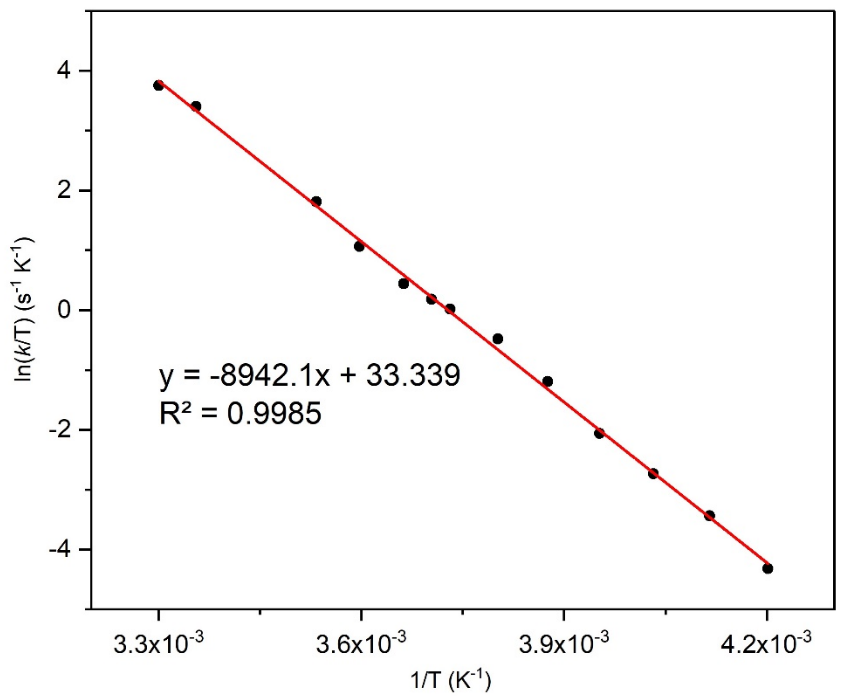


**Figure S11** Eyring-plot for the determination of ΔH‡ and ΔS‡ for the ring inversion in **[Cu(NHC$_4$)](PF$_6$)$_3$**. The following values were determined: ΔH‡ = 17.8 ± 0.2 kcal·mol$^{-1}$, ΔS‡ = 19.0 ± 0.8 cal·mol$^{-1}$ K$^{-1}$.

## UV-Vis and infrared spectroscopy

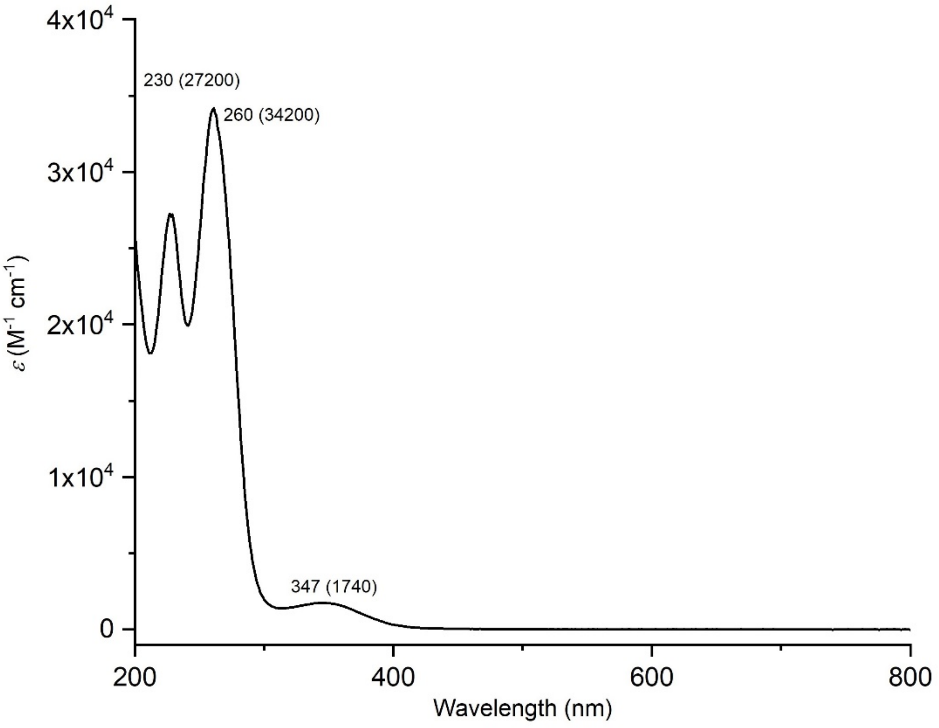


**Figure S12** UV-Vis spectra of **[Cu(NHC$_4$)](PF$_6$)$_3$** (in MeCN) recorded at room temperature.

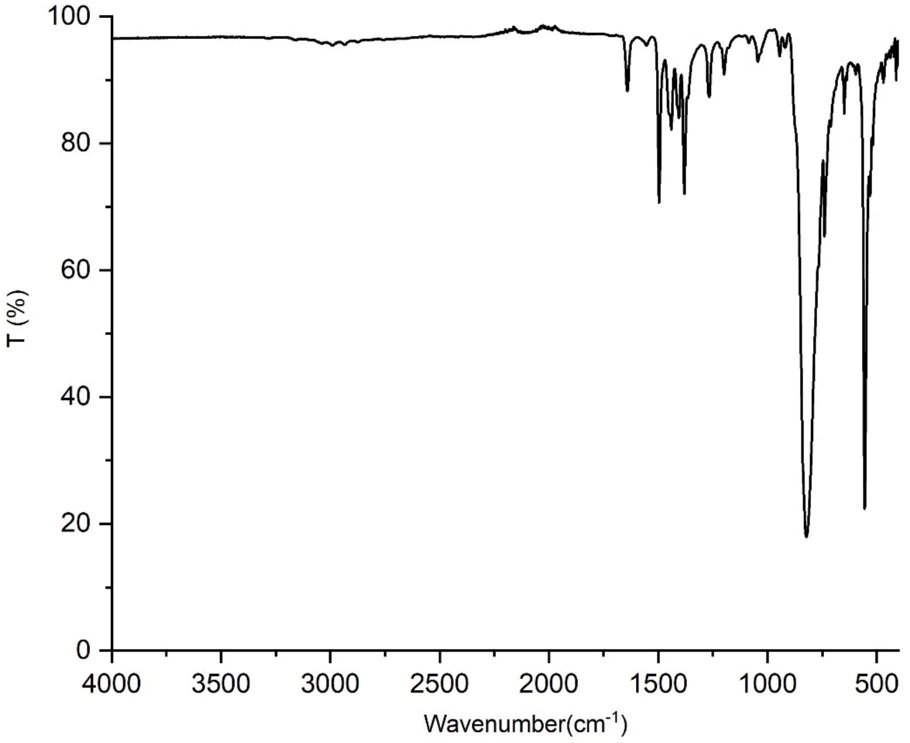


**Figure S13** ATR-IR spectrum of solid **[Cu(NHC$_4$)](PF$_6$)$_3$**.

## Mass Spectrometry

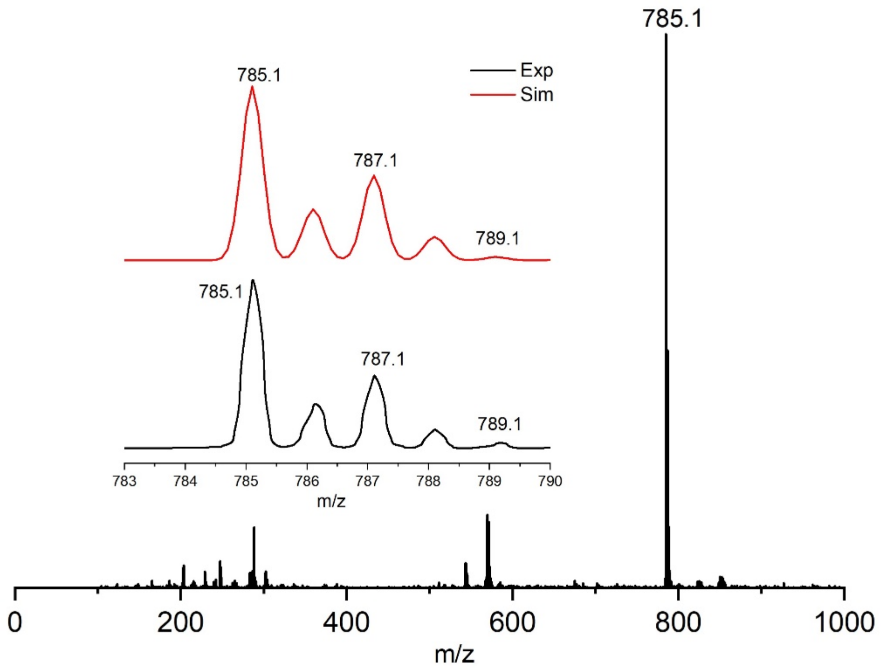


**Figure S14** ESI–MS of complex **[Cu(NHC$_4$)](PF$_6$)$_3$** in MeCN. The inset shows the experimental (bottom) and simulated (top) isotope pattern for the peak around m/z = 785.1 (100) [M-PF$_6$]$^+$.

## SQUID magnetometry

Temperature-dependent magnetic susceptibility measurements for **[Cu$^{III}$(NHC$_4$)](PF$_6$)$_3$** were carried out with a Quantum-Design MPMS-3 SQUID magnetometer equipped with a 7.0 T magnet in the range from 2-350 K on a polycrystalline powdered sample under an applied magnetic field of 0.5 T. The crystalline solid sample was contained in a polycarbonate capsule and fixed in a nonmagnetic sample holder. The raw data file for the measured magnetic moment was corrected for the diamagnetic contribution of the capsule according to $M^{dia} = \chi_g \times m \times H$, with experimentally obtained gram susceptibilities of the capsule. The molar susceptibility data of the compound were corrected for the diamagnetic contribution. Experimental data for **[Cu(NHC$_4$)](PF$_6$)$_3$** were modelled with the *julX* program.[19]

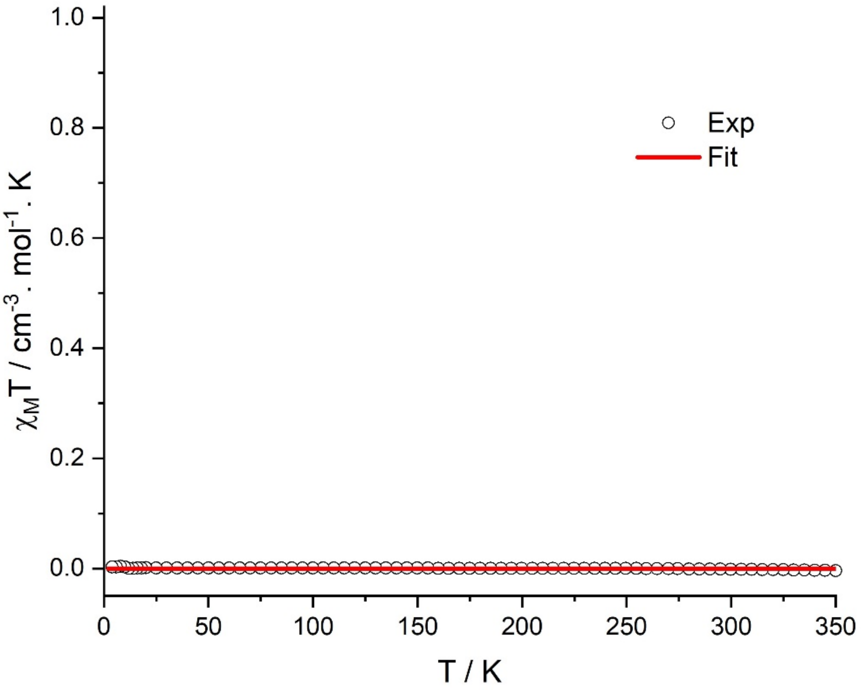


**Figure S15** $\chi_M T$ vs. $T$ measurement for a solid sample of **[Cu$^{III}$(NHC$_4$)](PF$_6$)$_3$** in the temperature range of 2-350 K at 0.5 T. The data shows that **[Cu$^{III}$(NHC$_4$)](PF$_6$)$_3$** is diamagnetic over the entire temperature range studied.

## X-ray Crystallography

**Table S2** Crystal data and structure refinement of $[Cu(NHC_4)](PF_6)_3 \cdot 2CH_3CN$

| **compound** | **1 (yl35)** |
|---|---|
| empirical formula | $C_{28}H_{38}CuF_{18}N_{10}P_3$ |
| moiety formula | $C_{24}H_{32}CuN_8^{3+}$, $3(F_6P^-)$, $2(C_2H_3N)$ |
| formula weight | 1013.13 |
| $T$ [K] | 100(2) |
| crystal size [mm³] | 0.380 x 0.126 x 0.071 |
| crystal system | orthorhombic |
| space group | $Pca2_1$ (No. 29) |
| $a$ [Å] | 18.4087(9) |
| $b$ [Å] | 12.9782(6) |
| $c$ [Å] | 16.4884(8) |
| $V$ [Å³] | 3939.3(3) |
| $Z$ | 4 |
| ρ [g·cm⁻³] | 1.708 |
| $F$(000) | 2048 |
| μ [mm⁻¹] | 0.800 |
| $T_{min}$ / $T_{max}$ | 0.85 / 0.94 |
| θ–range [°] | 2.213 – 27.895 |
| $hkl$–range | –23 to 24, ±17, ±21 |
| measured refl. | 37226 |
| unique refl. [$R_{int}$] | 9387 [0.0376] |
| observed refl. ($I > 2\sigma(I)$) | 8167 |
| data / restr. / param. | 9387 / 29 / 587 |
| goodness–of–fit ($F^2$) | 1.023 |
| $R$1, $wR$2 ($I > 2\sigma(I)$) | 0.0311 / 0.0750 |
| $R$1, $wR$2 (all data) | 0.0390 / 0.0804 |
| res. el. dens. [e·Å⁻³] | –0.381 / 0.574 |

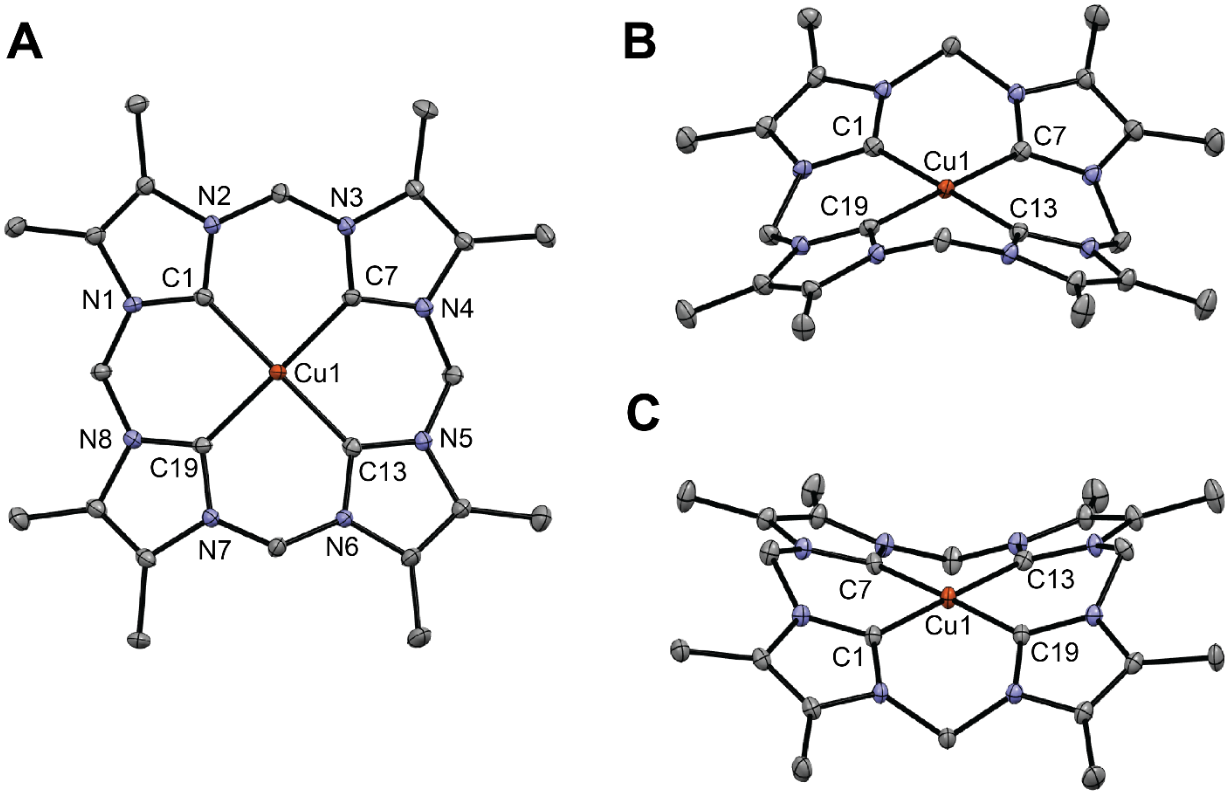


**Figure S16** A) Crystal structure of the complex cation $[Cu^{III}(NHC_4)]^{3+}$ as viewed down the molecular z-axis. B) View of the complex cation down one of its dihedral mirror planes ($\sigma_d^1$). C) View of the complex cation down the second of its dihedral mirror planes ($\sigma_d^2$). Acetonitrile solvates and $PF_6^-$ anions are omitted for clarity. Ellipsoids are shown at the 50% probability level. Legend: copper, orange; carbon, grey; nitrogen, blue.

**Table S3** Selected bond length and angles for the complex cation $[Cu^{III}(NHC_4)]^{3+}$.

| **Bonds** | **$[Cu^{III}(NHC_4)]^{3+}$** |
|---|---|
| Bond lengths [Å] | |
| Cu1-C1 | 1.877(3) |
| Cu1-C7 | 1.881(3) |
| Cu1-C13 | 1.889(3) |
| Cu1-C19 | 1.881(3) |
| Av. Cu-C | 1.882(3) |
| | |
| Bond angles [°] | |
| C1-Cu1-C13 | 178.90(14) |
| C7-Cu1-C19 | 178.11(14) |
| C1-Cu1-C7 | 89.60(14) |
| C1-Cu1-C19 | 89.57(13) |
| C7-Cu1-C13 | 90.56(13) |
| C13-Cu1-C19 | 90.25(14) |
| Av. *trans* C-Cu-C | 178.51(14) |
| Av. *cis* C-Cu-C | 90.00(14) |

# X-ray Spectroscopy

## X-ray Absorption

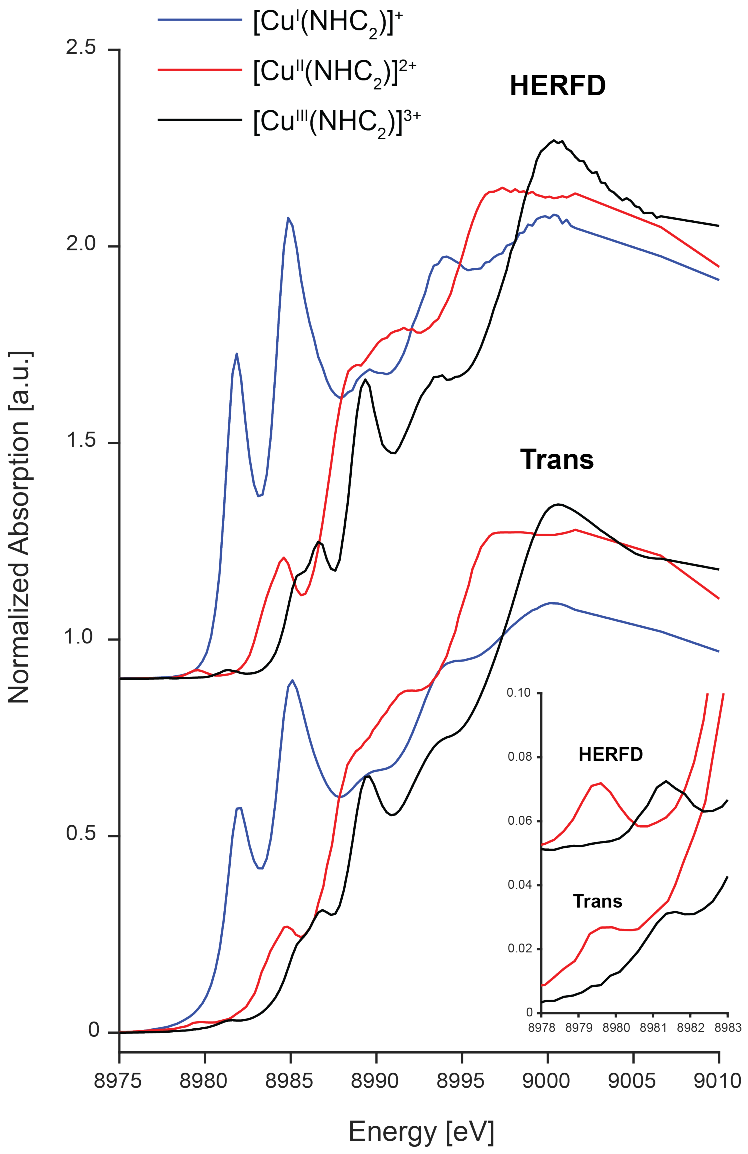


**Figure S17** Comparison of the Kβ high energy resolution fluorescence detected (HERFD)[1] and Transmission mode Cu K-edge XAS for the $[Cu^{n+}(NHC_2)]^{n+}$ series. The inset shows the magnification of the pre-edge region for the Cu(II) and Cu(III) species.

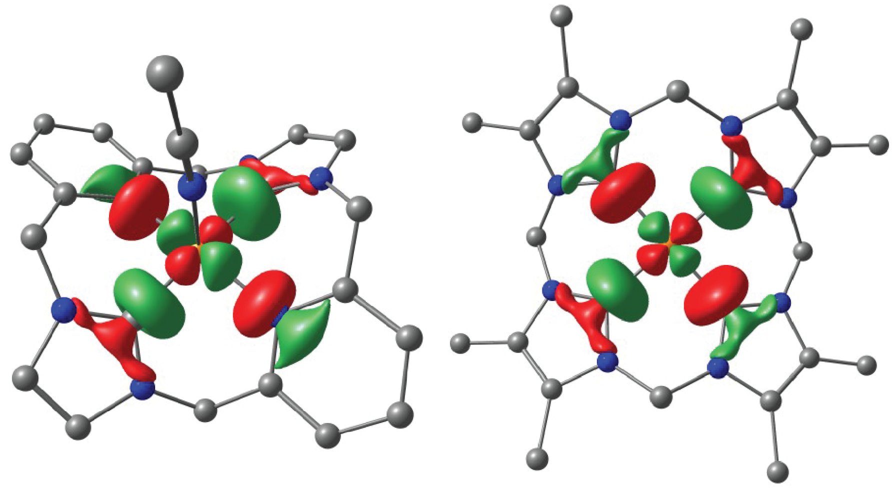

$[Cu^{III}(NHC_2)]^{3+}$ $[Cu^{III}(NHC_4)]^{3+}$

**Figure S18** B3LYP/def2-TZVP calculated LUMOs for Cu(III) complexes of the $NHC_2$ (left) and $NHC_4$ (right) macrocyclic ligands. Isosurfaces are plotted at 0.05 a.u.

**Table S4** Selected orbital energies and corresponding Cu d/p orbital character for $[Cu^{III}(NHC_4)]^{3+}$ and $[Cu^{III}(CF_3)_4]^{3+}$. Orbital compositions were determined from reduced Löwdin orbital population analysis.

| | $[Cu^{III}(NHC_4)]^{3+}$ | | | $[Cu^{III}(CF_3)_4]^{-}$ | | |
|---|---|---|---|---|---|---|
| Orbital | Energy [eV] | Cu *n*d orbital [%] | Cu *n*p orbital [%] | Energy [eV] | Cu *n*d orbital [%] | Cu *n*p orbital [%] |
| LUMO +2 | -0.86 | 0.7 xz, 7.6 yz | 0 | 0.97 | 11.4 $z^2$ | 0 |
| LUMO +1 | -2.25 | 0 | 15.1 $p_z$ | 0.23 | 3.2 $x^2$-$y^2$ | 32.2 $p_z$, 0.9 $p_y$ |
| LUMO | -3.29 | 43.4 $x^2$-$y^2$ | 0 | -2.64 | 35.5 $x^2$-$y^2$ | 3.5 $p_z$ |
| HOMO | -6.97 | 0 | 0 | -7.51 | 1.2 xy, 0.8 yz | 5.6 $p_x$, 6.6 $p_y$ |
| HOMO-1 | -7.02 | 2.9 xy, 0.9 yz | 0 | -7.53 | 1.0 xy, 1.1 yz | 6.7 $p_x$, 5.3 $p_y$ |
| HOMO-2 | -7.02 | 0 | 2.7 $p_z$ | -8.83 | 34.8 $z^2$ | 0 |
| HOMO-3 | -7.44 | 0 | 0 | -10.03 | 80.4 yz | 0 |
| HOMO-8 | -9.67 | 0.1 xz, 0.1 yz | 5.7 $p_x$, 6.7 $p_y$ | -10.75 | 0 | 0 |
| HOMO-9 | -9.68 | 0.1 xz, 0.1 yz | 6.7 $p_x$, 5.7 $p_y$ | -10.87 | 0 | 0 |

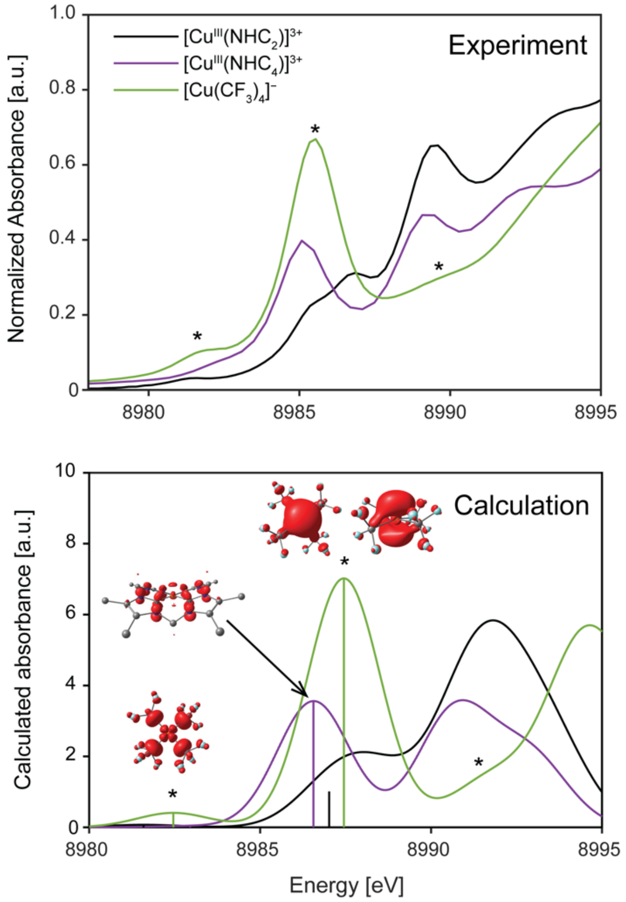


**Figure S19** Experimental (top) and TDDFT calculated (bottom) XAS for the three Cu(**III**) complexes investigated along with transition difference densities for selected pre-edge and rising edge transitions. transition difference densities are plotted with an isosurface value of 0.005 a.u. Calculated spectra are shifted -3.1 eV.

## X-ray Emission

### Cu Kβ Mainlines

**Table S5** Energies of the Kβ' and Kβ$_{1,3}$ features, and their separation, for all complexes considered in the present study.

| | **Kβ' (eV)** | **Kβ$_{1,3}$ (eV)** | **Δ*E* (eV)** |
|---|---|---|---|
| CuCl | 8895.2 | 8904.6 | 9.4 |
| $CuCl_2$ | 8894.7 | 8904.7 | 9.9 |
| $CuF_2$ | 8896.5 | 8904.7 | 8.2 |
| [Cu$^{I}$(NHC$_2$)]$^{+}$ | 8895.3 | 8904.8 | 9.5 |
| [Cu$^{II}$(NHC$_2$)]$^{2+}$ | 8895.1 | 8904.9 | 9.8 |
| [Cu$^{III}$(NHC$_2$)]$^{3+}$ | 8895.7 | 8904.9 | 9.2 |
| [Cu$^{III}$(NHC$_4$)]$^{3+}$ | 8895.1 | 8904.9 | 9.8 |
| [Cu$^{III}$(CF$_3$)$_4$]$^{-}$ | 8895.7 | 8904.9 | 9.2 |

## Cu Kβ VtC XES

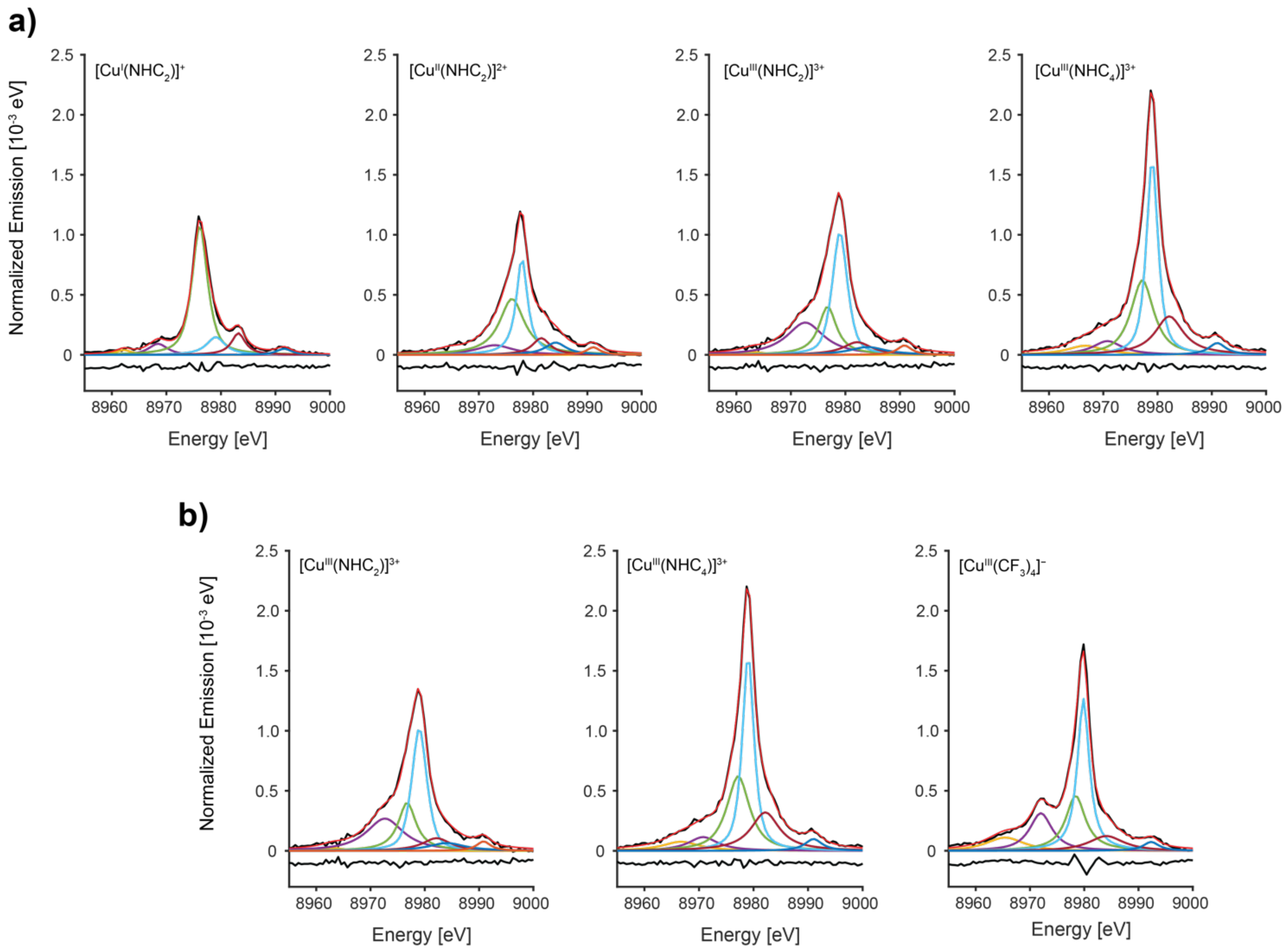


**Figure S20** a) Background subtracted VtC spectra for the [Cu$^{n+}$(NHC$_2$)]$^{n+}$ series and [Cu$^{III}$(NHC$_4$)]$^{3+}$ and b) Background subtracted VtC spectra for the Cu(III) complexes [Cu$^{III}$(NHC$_2$)]$^{3+}$, [Cu$^{III}$(NHC$_4$)]$^{3+}$ and [Cu$^{III}$(CF$_3$)$_4$]$^{-}$ with individual fitted Gaussian bands (colored) and their sum (red). Residual intensity (experiment – fit) are shown in black below the individual VtC spectra.

**Table S6** Peak analysis for the single dominant VtC emission transition in all complexes.

| Compound | Energy [eV] | FWHM [eV] | Integrated Intensity[a] |
|---|---|---|---|
| [Cu$^{I}$(NHC$_2$)]$^{+}$ | 8975.9 | 1.59 | 5.0 |
| [Cu$^{II}$(NHC$_2$)]$^{2+}$ | 8977.9 | 1.23 | 3.1 |
| [Cu$^{III}$(NHC$_2$)]$^{3+}$ | 8979.0 | 1.51 | 4.4 |
| [Cu$^{III}$(NHC$_4$)]$^{3+}$ | 8979.0 | 1.21 | 5.9 |
| [Cu$^{III}$(CF$_3$)$_4$)]$^{-}$ | 8979.9 | 1.30 | 5.0 |

[a] Integrated intensities were multiplied by 1000.

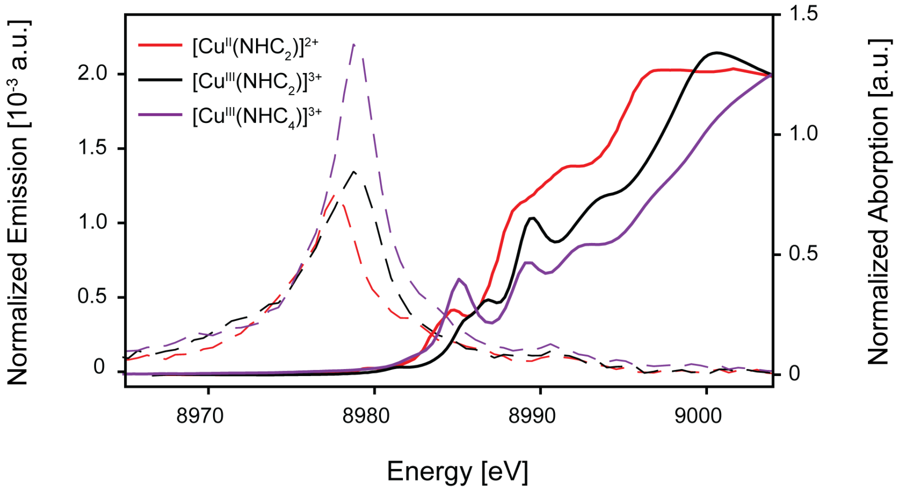


**Figure S21** Overlay of the high energy Cu Kβ VtC XES region (dashed) and the edge region of the Cu K-edge XAS (solid) for $[Cu^{II}(NHC_2)]^{2+}$, $[Cu^{III}(NHC_2)]^{3+}$ and $[Cu^{III}(NHC_4)]^{3+}$.

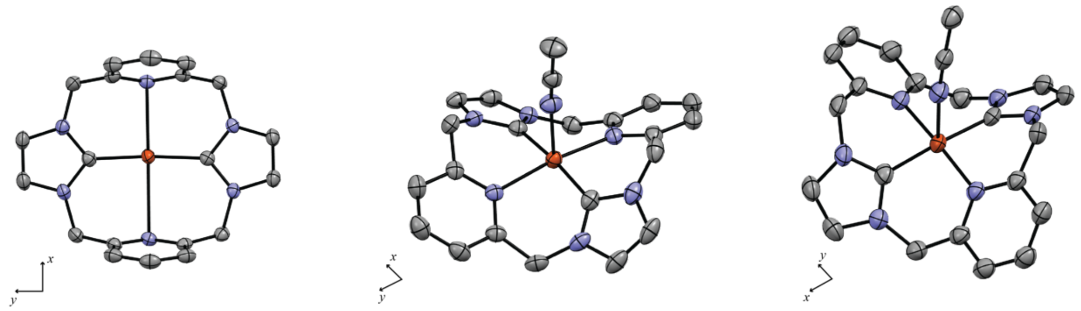


**Figure S22** Crystal structures of the cationic units of the $[Cu^{n+}(NHC_2)]^{n+}$ oxidation series. Legend: carbon, gray; copper, orange; nitrogen, blue. Non-coordinated solvates, anion and hydrogen atoms are omitted for clarity. Thermal ellipsoids are shown at the 50% probability level.

**Table S7** Single point energies for the singlet and triplet state in Cu(III) complexes relative to their minimum energy solution.

| | $[Cu^{III}(NHC_2)]^{3+}$ | $[Cu^{III}(NHC_4)]^{3+}$ | $[Cu(CF_3)_4]^-$ |
|---|---|---|---|
| **Singlet energy [eV]** | | | |
| UKS | 0 | 0 | 0 |
| RKS | 0 | 0 | 0 |
| BS ($M_S$ = 0)[a] | N/A | N/A | N/A |
| **Triplet energy [eV]** | | | |
| UKS[b] | +1.5 | +3.0 | +1.4 |

[a] Broken symmetry DFT single point calculation was conducted but no solution was found. [b] Triplet state energy calculated via a single point calculation using the xyz file obtained from the geometry optimization of the Cu(III) complex in the triplet state.

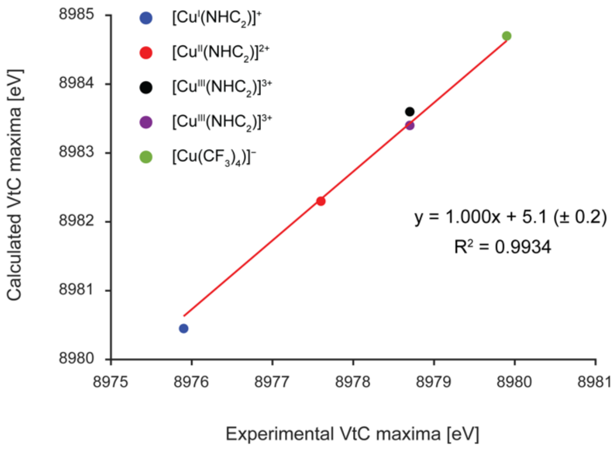


**Figure S23** Energies of the calculated vs. experimental emission maxima in the VtC region of the Cu Kβ XES for all complexes. The red line is a linear fit of the data with the slope fixed to a value of 1.000 to extract a constant energy shift from the y intercept.

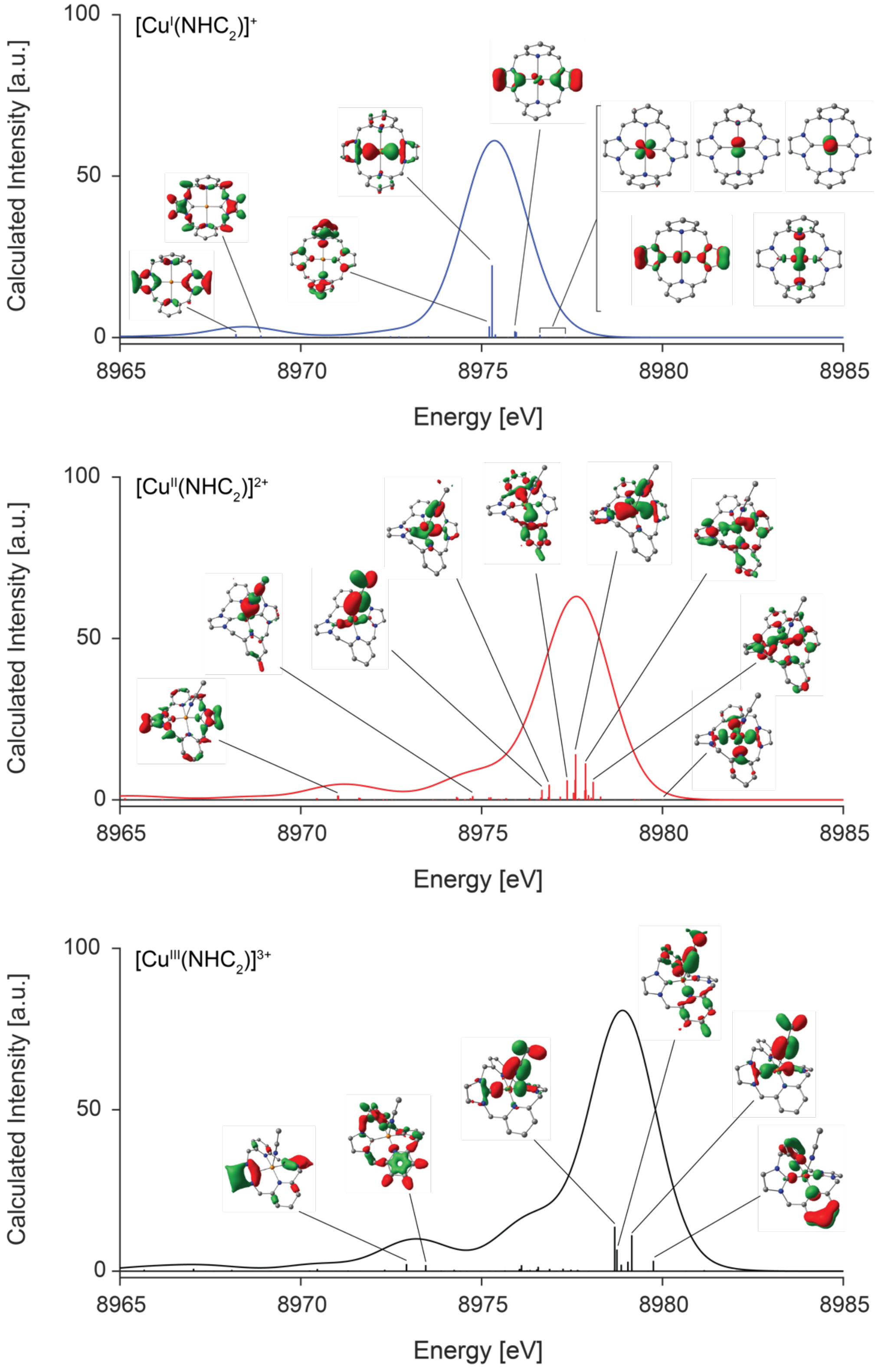


**Figure S24** Calculated VtC spectra for $[Cu^{I}(NHC_2)]^{+}$, $[Cu^{II}(NHC_2)]^{2+}$ and $[Cu^{III}(NHC_2)]^{3+}$ with donor MOs corresponding to the most intense one-electron transitions. Isosurfaces are plotted at a value of 0.05 a.u. Spectra are shifted by -5.1 eV and a linewidth broadening of 2.0 eV was applied.

**Table S8** Calculated orbital contributions to the donor MOs responsible for the main K$\beta_{2,5}$ feature in the VtC spectra. Values are obtained from both α and β orbital sets and the VtC region was determined by summing the intensities of transitions encompassed by a gaussian line function fit to the selected energy region of the VtC spectrum.

| | % Cu *n*p | % Cu *n*d | % ligand *n*p | % ligand *n*s |
|---|---|---|---|---|
| $[Cu^{I}(NHC_2)]^{+}$ | 7.3 | 8.6 | 64.8 | 11.1 |
| $[Cu^{II}(NHC_2)]^{2+}$ | 4.4 (2.7)[a] | 14.4 (15.6)[a] | 63.5 (64.4)[a] | 9.7 (8.3)[a] |
| $[Cu^{III}(NHC_2)]^{3+}$ | 3.5 | 6.6 | 70.0 | 12.2 |
| $[Cu^{III}(NHC_4)]^{3+}$ | 9.2 | 1.8 | 65.8 | 15.5 |
| $[Cu^{III}(CF_3)_4]^{-}$ | 10.1 | 5.3 | 71.2 | 8.8 |

[a] In open shell complexes, values outside of brackets are given for the α spin manifold, values given inside brackets are for the β spin manifold. MO range for the accumulative Löwdin population analysis were: $[Cu^{I}(NHC_2)]^{+}$ = MO52a-HOMO; $[Cu^{II}(NHC_2)]^{2+}$ = MO60a-(SOMO); $[Cu^{III}(NHC_2)]^{3+}$ = MO60a-(HOMO); $[Cu^{III}(NHC_4)]^{3+}$ = 59a-HOMO; $[Cu^{3+}(CF_3)_4]^{-}$ = 42a-HOMO.

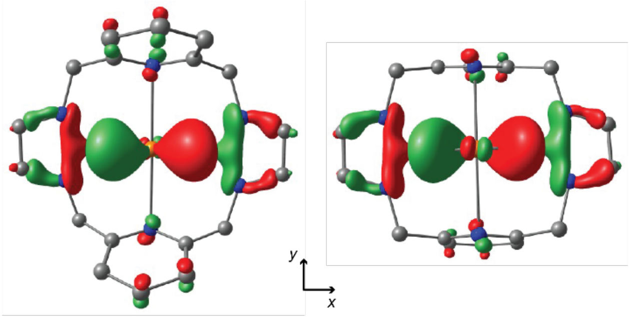


$[Cu^{I}(NHC_2)]^{+}$ HOMO−14

**Figure S25** (Cu) $3d_{xz}$–$2p_x$ (C) $b_2$ σ-type MO in $[Cu^{I}(NHC_2)]^{+}$ as viewed along the principle $C_2$ axis (z). Isosurfaces are plotted at a value of 0.05 a.u.

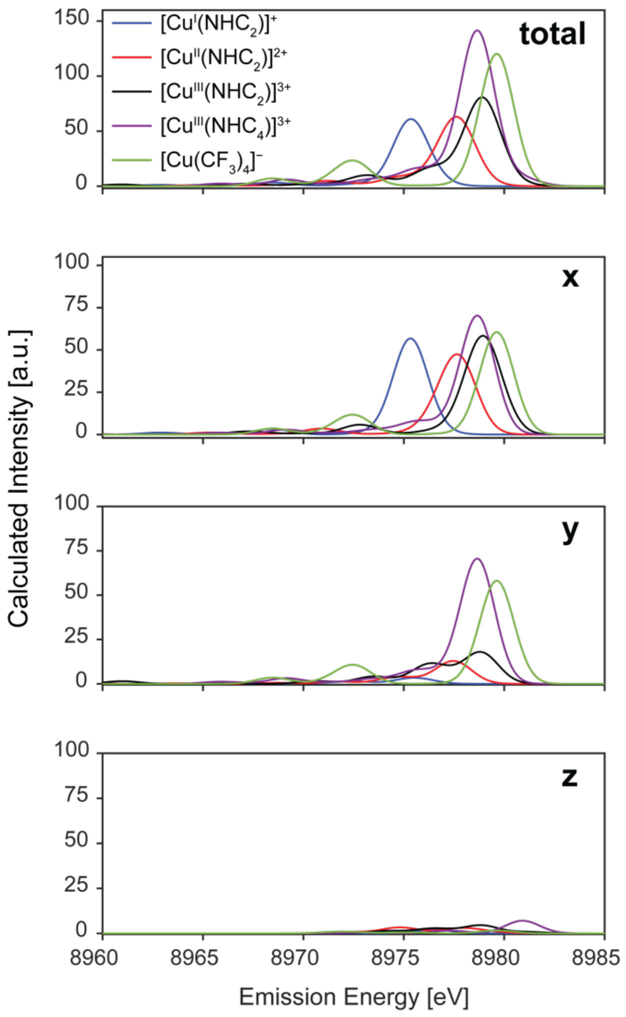


**Figure S26** Calculated total and *x*,*y*,*z* polarized VtC spectra for the complexes investigated in the present work. The *x*,*y* and *z* axes are as depicted in Scheme 1.

Due to the relatively high symmetry and approximate planar structure of the $[Cu^{n+}(NHC_{2/4})]^{n+}$ complexes, the calculated polarized VtC XES plots are highly informative to understand various intensity contributions. Figure S26 shows that the majority, if not all the intensity for the $[Cu^{n+}(NHC_2)]^{n+}$ series is x-polarized, corresponding to the Cu-C$^{NHC}$ vector. Each of the $[Cu^{n+}(NHC_2)]^{n+}$ complexes exhibit a dominant peak along the x-direction of comparable intensity, although $[Cu^{II}(NHC_2)]^{2+}$ is noticeably the least intense as well as being broader due to the distribution of transitions (Figures 5 and S24). Despite the decreasing average C$^{NHC}$-Cu bond lengths from the Cu(I) species (1.94 Å) to the Cu(II) (1.91 Å) and Cu(III) (1.88 Å), there is not a clear trend of integrated intensity. However, inspection of the C-Cu-C angles in these complexes shows that the angle in the Cu(I) species (173.9°) and the Cu(III) species (172.7°) are closer to linear than that in the Cu(II) species (169.0°). The more linear C-Cu-C σ bonds in the Cu(I) and Cu(III) species will have better overlap with the Cu $np_x$ orbitals, thus resulting in larger intensity being imparted into the transition. This shows that the decreasing Cu-C bond lengths in the Cu(II) species relative to the Cu(I) species are counteracted by the geometry changes resulting from the oxidation of the Cu center. Such an angular dependence on the VtC spectrum of hydroxo and oxo-bridged Fe dimers, as well as Ni $NO_x$ complexes has been demonstrated previously.[20,21]

In the orthogonal y-direction, along the Cu-N$^{pyridine}$ vector, the calculated VtC XES exhibits minimal intensity for the $[Cu^{I}(NHC_2)]^{+}$ complex, consistent with the pyridines being assigned as non-coordinating. However, for $[Cu^{II}(NHC_2)]^{2+}$ and $[Cu^{III}(NHC_2)]^{3+}$, an increasing amount of intensity is observed in the y-polarized direction due to the coordinating pyridines (Figure S26). Inspection of the MOs corresponding to the most dominant transitions qualitatively reveals localized pyridine character. Furthermore, inspection of the Löwdin population analysis of the MOs corresponding to the transitions in this shoulder region reveals increased pyridine p-orbital character relative to the Cu(I) species (Table S9). Lastly, both $[Cu^{II}(NHC_2)]^{2+}$ and $[Cu^{III}(NHC_2)]^{3+}$ exhibit a small amount of z-polarized intensity that may also be attributed to either the weakly coordinated acetonitrile ligand or a slight geometric distortion (Figures S22 and S26).

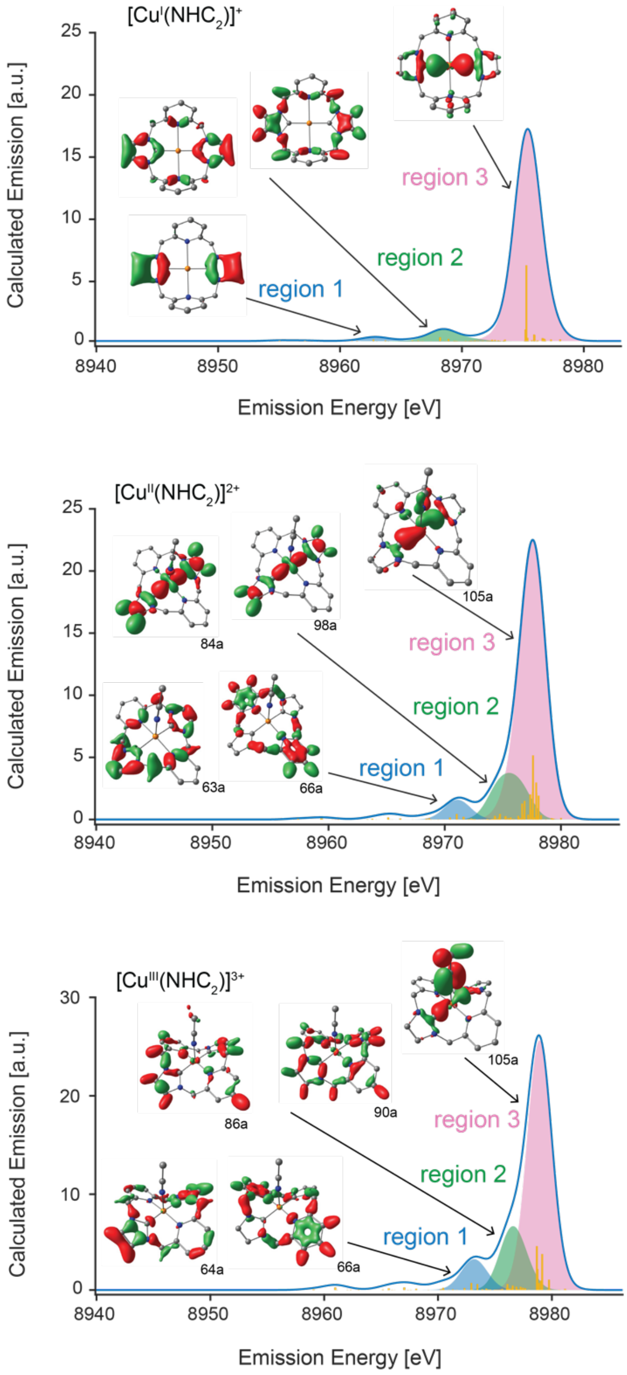


**Figure S27** Summation of the emission intensity in three main regions of interest in the calculated VtC spectra for $[Cu^{I}(NHC_2)]^{+}$ (top), $[Cu^{II}(NHC_2)]^{2+}$ (middle) and $[Cu^{III}(NHC_2)]^{3+}$ (bottom). MO isosurfaces are plotted at a value of 0.05 a.u.

**Table S9** Löwdin populations of canonical orbitals contributing to the calculated emission intensity of the $[Cu^{n+}(NHC_2)]^{n+}$ series in three different regions of interest as shown in Figure S27.

| | **Cu d,p [%]** | **Total ligand s,p [%]** | **NHC s,p [%]** | **Pyridine s,p [%]** | **Acetonitrile s,p [%]** |
|---|---|---|---|---|---|
| **$[Cu^{I}(NHC_2)]^{+}$** | | | | | |
| Region 1 | 0, 0 | 53.0, 39.1 | 34.0, 27.5 | 14.2, 8.9 | - |
| Region 2 | 0, 0.6 | 28.1, 66.6 | 14.4, 40.1 | 5.1, 16.3 | - |
| Region 3 | 9.3, 7.9 | 9.8, 64.6 | 8.0, 40.9 | 0.8, 21.9 | - |
| **[a]$[Cu^{II}(NHC_2)]^{2+}$** | | | | | |
| Region 1 | 0.1, 1.0 | 22.9, 73.3 | 11.9, 33.9 | 5.2, 26.7 | 1.0, 7.6 |
| Region 2 | 17.7, 0.8 | 13.7, 60.6 | 1.9, 15.6 | 4.6, 20.4 | 2.2, 12.5 |
| Region 3 | 16.7, 4.1 | 7.1, 63.1 | 2.8, 21.9 | 1.7, 27.5 | 1.4, 9.3 |
| **$[Cu^{III}(NHC_2)]^{3+}$** | | | | | |
| Region 1 | 0.4, 1.4 | 20.4, 75.1 | 9.2, 31.5 | 6.2, 29.7 | 0.2, 0.4 |
| Region 2 | 18.3, 0.6 | 17.4, 57.6 | 3.3, 18.6 | 4.9, 19.7 | 2.1, 7.2 |
| Region 3 | 4.9, 4.5 | 9.4, 72.0 | 2.8, 18.7 | 1.9, 20.7 | 4.2, 30.8 |

[a] Values for the $[Cu^{II}(NHC_2)]^{2+}$ species are the average for the α and β spin manifolds.

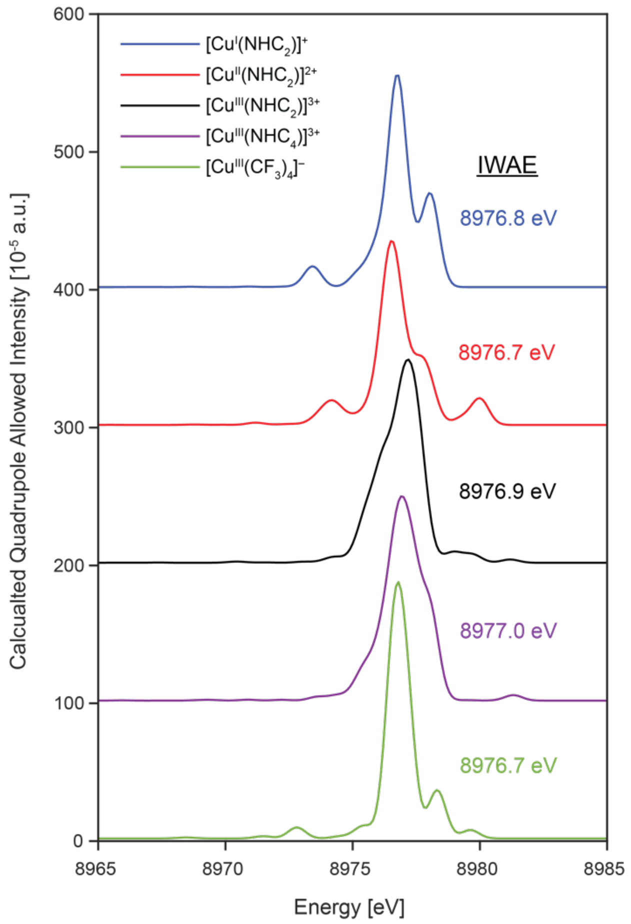


**Figure S28** DFT calculated quadrupole allowed only VtC emission spectra and respective IWAEs for all complexes in the present analysis. A Gaussian line broadening function was used and a FWHM value of 0.8 eV was selected.

**Table S10** B3LYP/def2-TZVP calculated total Cu *n*d orbital admixture into valence MOs (and total Cu spin population) given by Löwdin and Mulliken population analysis of either the canonical orbitals, unrestricted natural orbitals (UNOs) or quasi restricted orbitals (QROs).

| | **% Cu *n*d admixture** | | **Cu spin population [%]** |
|---|---|---|---|
| | Löwdin canonical (UNO) [QRO] | Mulliken canonical (UNO) [QRO] | Löwdin (Mulliken) canonical |
| [Cu$^{I}$(NHC$_2$)]$^+$ HOMO | 58.3 (1.1) [58.3] | 57.4 (0.4) [57.5] | - |
| [Cu$^{II}$(NHC$_2$)]$^{2+}$ HOMO | 29.5 (61.0) [61.0] | 27.3 (59.8) [60.0] | 61.5 (61.7) |
| [Cu$^{III}$(NHC$_2$)]$^{3+}$ LUMO | 42.0 (2.4) [42.0] | 37.3 (1.6) [37.3] | - |
| [Cu$^{III}$(NHC$_4$)]$^{3+}$ LUMO | 45.2 (0.6) [47.4] | 33.8 (0.6) [35.2] | - |
| [Cu$^{III}$(CF$_3$)$_4$]$^-$ LUMO | 38.8 (7.3) [38.0] | 24.1 (7.6) [24.4] | - |
| D$_{4h}$ [CuCl$_4$]$^{2-}$ HOMO | 20.2 (58.9) [59.0] | 15.5 (56.6) [56.6] | 55.6 (51.5) |

Values are given for the α orbitals.

## UV-Vis

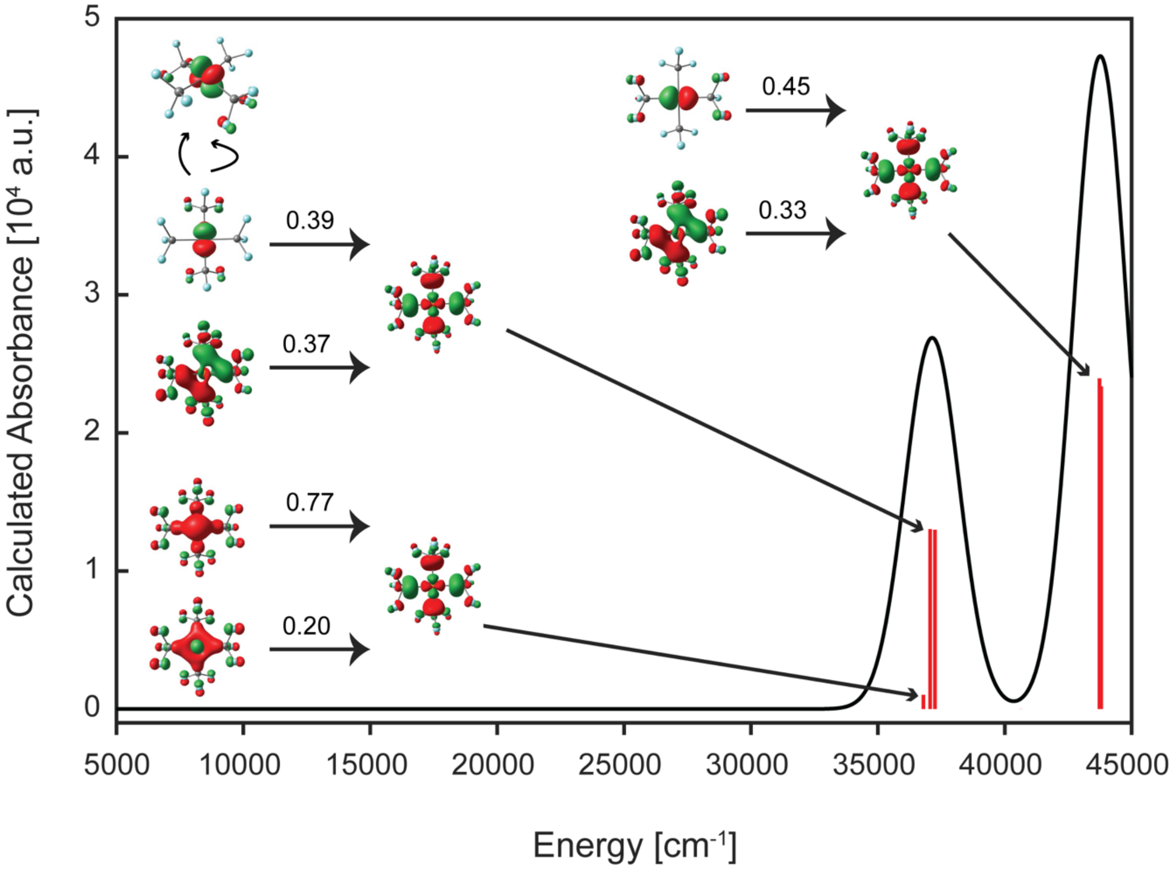


**Figure S29** TDDFT calculated (B3LYP/ZORA-def2-TZVP/ZORA) UV-Vis spectrum for $[Cu(CF_3)_4]^-$ using the conductor-like polarizable continuum model (ε = inf.) to account for the negative charge of the complex anion. Donor and acceptor MOs are plotted for selected transitions along with their weighted contributions. Isosurfaces are plotted at a value of 0.05 a.u.

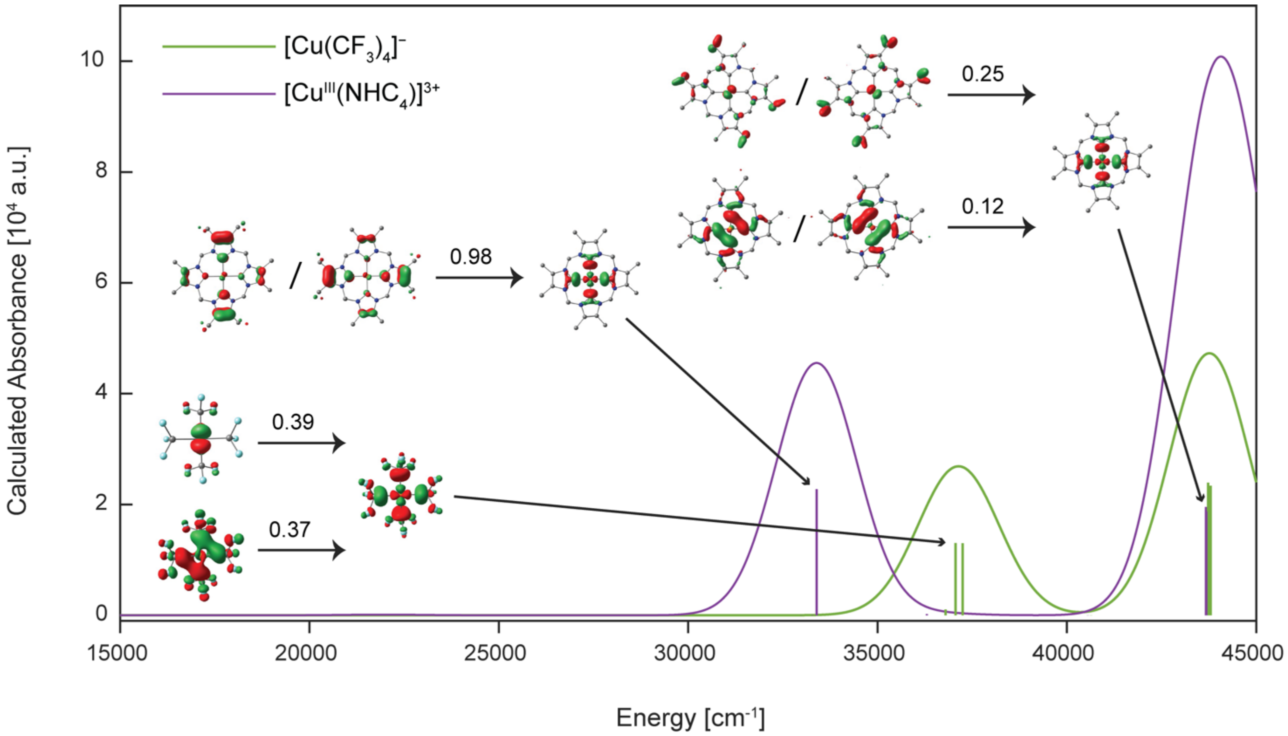


**Figure S30** TDDFT calculated (B3LYP/ZORA-def2-TZVP/ZORA) UV-Vis spectrum for $[Cu(CF_3)_4]^-$ and $[Cu^{III}(NHC_4)]^{3+}$ using the conductor-like polarizable continuum model (ε = inf.) to account for the overall charge of the complex ions. Donor and acceptor MOs are plotted for selected transitions along with their weighted contributions. Isosurfaces are plotted at a value of 0.05 a.u.

## Example Orca Input Files

### Geometry Optimization

```
! UKS RIJCOSX B3LYP def2-TZVP AutoAux opt TightSCF Grid4 FinalGrid7 SlowConv NormalPrint
! UNO CPCM(if complex carries non-zero charge)

%pal
nprocs 12
end

%maxcore 3000

%scf
MaxIter 700
End

*xyzfile charge spin-multiplicity xyz_filename.xyz
```

### X-ray Absorption Spectroscopy

```
! UKS RIJCOSX B3LYP ZORA ZORA-def2-TZVP AutoAux TightSCF SlowConv Grid4 FinalGrid7 Pal8 LargePrint
! CPCM UNO

%scf
Maxiter 700
end

%maxcore 3000

%tddft
NRoots 200
MaxDim 900
OrbWin[0] = 0, 0, -1, -1
OrbWin[1] = 0, 0, -1, -1
Doquad true
end

*xyzfile charge spin_multiplicity xyz_filename.xyz
```

### X-ray Emission Spectroscopy

```
! UKS RIJCOSX B3LYP ZORA ZORA-def2-TZVP AutoAux Grid4 FinalGrid7 SlowConv TightSCF pal8
! LargePrint UNO CPCM(if complex carries non-zero charge)

%scf
MaxIter 700
end

%maxcore 3000

%xes
```

```
CoreOrb 0,0
OrbOp 0,1
CoreOrbSOC 0,1
DoSOC true
end

*xyzfile charge spin_multiplicity xyz_filename.xyz
```

**Löwdin and Mulliken Population analysis of QROs**

```
! LargePrint NoIter MOREAD def2-TZVP

%moinp "tddft_calculation_filename.qro"

*xyzfile charge spin_multiplicity xyz_filename.xyz
```

**Broken Symmetry**

```
! UKS RIJCOSX B3LYP def2-TZVP AutoAux Grid4 FinalGrid7 SlowConv TightSCF pal8
! LargePrint UNO CPCM

%scf
BrokenSym 1,1
end

%maxcore 3000

*xyzfile charge triplet_spin_multiplicity xyz_filename.xyz
```

**UV-vis spectroscopy**

```
! UKS RIJCOSX B3LYP ZORA ZORA-def2-TZVP AutoAux Grid4 FinalGrid7 SlowConv
! TightSCF pal8 NormalPrint UNO CPCM

%scf
MaxIter 700
end

%maxcore 3000

%tddft
NRoots 60
MaxDim 5
end

*xyzfile charge triplet_spin_multiplicity xyz_filename.xyz
```

## XYZ Coordinates for Orca Calculations

**[Cu$^{I}$(NHC$_2$)]$^+$**

Charge = 1+, Spin Multiplicity = 1

| | | | |
|---|---|---|---|
| Cu | -0.00740413299260 | -0.04079336237917 | 0.13207803040467 |
| N | -0.02306510512784 | -2.62451096464465 | 0.39810987921352 |
| N | 0.00932859614757 | 2.56090157725132 | 0.28689515106662 |
| C | 1.94728414746307 | -0.07464419591113 | 0.22304221810076 |
| C | -1.96070678328296 | 0.00365151709803 | 0.24885425026110 |
| C | 1.08157935414333 | -2.80336943362877 | 1.12738717334633 |
| C | -1.21411673613655 | -2.79297614879768 | 0.97584022771510 |
| C | -1.08332126019588 | 2.76462195233140 | 1.02745388054978 |
| C | 1.20918221142673 | 2.74988236796822 | 0.83932751055978 |
| N | 2.77476254700957 | -1.13712084620435 | 0.39209544958973 |
| N | 2.79304105107554 | 0.98787826466697 | 0.18784269586343 |
| N | -2.78672815754387 | 1.07280132298858 | 0.37910296228816 |
| N | -2.80684976197257 | -1.05915199921355 | 0.26438925445634 |
| C | 2.37810980747925 | -2.55535313027989 | 0.40701562104720 |
| C | 1.03649430977973 | -3.16881219046406 | 2.46758777732075 |
| C | -1.35064324389426 | -3.17142403630490 | 2.30676528152688 |
| C | -2.40085380900937 | -2.46631470888475 | 0.11221353571653 |
| C | -2.39162657503318 | 2.49119399335755 | 0.33812444381695 |
| C | -1.01655894519240 | 3.17624761781704 | 2.35313538783983 |
| C | 1.36735837672303 | 3.17618543289052 | 2.15318018745124 |
| C | 2.38373841133867 | 2.38632893324904 | -0.02548123351970 |
| C | 4.10057715692895 | -0.74861859514705 | 0.44610284290877 |
| C | 4.11180015935928 | 0.59674217931508 | 0.31921745065780 |
| C | -4.11228945727196 | 0.68712224807323 | 0.45745623321823 |
| C | -4.12467488342361 | -0.66230320389551 | 0.38682817435480 |
| C | -0.20365214973805 | -3.35912182510847 | 3.06403240449292 |
| C | 0.23298074223064 | 3.38932364419960 | 2.92192097796087 |
| H | 3.18870313031190 | -3.10199987409391 | 0.88468436480272 |
| H | 2.28322449815311 | -2.90045117229094 | -0.62234805289014 |
| H | 1.94963117983410 | -3.31241259409035 | 3.02998583768353 |
| H | -2.33170808456379 | -3.31416484129875 | 2.74082452252246 |
| H | -2.14975535790919 | -2.65555525230473 | -0.93040014891398 |
| H | -3.26187618230452 | -3.07406076961440 | 0.38354869009515 |
| H | -3.19455076072294 | 3.05305206474972 | 0.81098475814832 |
| H | -2.31558271709328 | 2.80123440237509 | -0.70399280345024 |
| H | -1.92058719760087 | 3.33742714306229 | 2.92528875481498 |
| H | 2.35541366793588 | 3.33417698636880 | 2.56562933115066 |
| H | 2.11753966469871 | 2.52565130381315 | -1.07220997868685 |
| H | 3.24760556566286 | 3.00677083226666 | 0.20491660408108 |

| | | | |
|---|---|---|---|
| H | 4.90492510117534 | -1.45075645416561 | 0.57301593914522 |
| H | 4.92890452820429 | 1.29540084567437 | 0.31498690058940 |
| H | -4.91574059417615 | 1.39410577352380 | 0.56084134748227 |
| H | -4.94171318776417 | -1.36039101945015 | 0.41814394547521 |
| H | -0.27366218218347 | -3.65684137259083 | 4.10188738919502 |
| H | 0.31966790105189 | 3.72258782672313 | 3.94755548354672 |

**$[Cu^{II}(NHC_2)]^{2+}$**

Charge = 2+, Spin Multiplicity = 2

| | | | |
|---|---|---|---|
| Cu | 0.10082553881777 | -0.01162902283546 | 0.01361636182430 |
| N | 0.09177864474464 | -2.19517970873212 | 0.32308094264054 |
| N | 0.09137194740309 | 2.19001728077502 | 0.10570309438298 |
| N | 0.10827421165350 | -0.07099431073676 | -2.35899804452316 |
| C | 2.01124054044758 | -0.01428398467647 | 0.28199763732638 |
| C | -1.82323092576793 | 0.01339166647361 | 0.11270973440238 |
| C | 1.18034605791675 | -2.91675405129246 | -0.00751777317502 |
| C | -0.99026703699657 | -2.83143876581840 | 0.80973520770289 |
| C | -0.93174111333120 | 2.86976818341817 | -0.44209041501289 |
| C | 1.14371215887214 | 2.87334360283847 | 0.59769502669000 |
| C | -0.11718869170953 | 0.02223724123049 | -3.48195327172126 |
| N | 2.84037624779476 | -1.02856194168101 | 0.00799105287700 |
| N | 2.77700097053876 | 0.95431556545254 | 0.80012869398815 |
| N | -2.61612552028967 | 0.99388483820824 | -0.34147234682676 |
| N | -2.64373123466321 | -0.91174734175171 | 0.62498812702716 |
| C | 2.38269983086823 | -2.24831526300419 | -0.64617296499871 |
| C | 1.21761556807379 | -4.29737019646537 | 0.14632964023301 |
| C | -1.02253367749143 | -4.21586666791314 | 0.94268663194138 |
| C | -2.17909959104891 | -2.07492271707419 | 1.36978218695061 |
| C | -2.10415391943216 | 2.14384708407699 | -1.07304378293417 |
| C | -0.92461996202856 | 4.25766057168222 | -0.52933508712561 |
| C | 1.21860276702133 | 4.25803486249495 | 0.50658027466162 |
| C | 2.22929371133160 | 2.17144489220797 | 1.38788909987281 |
| C | -0.41604792704665 | 0.15140308518597 | -4.89588906257067 |
| C | 4.14517063482166 | -0.70555602282581 | 0.35413558928361 |
| C | 4.10388707112358 | 0.54935114347680 | 0.86280404025582 |
| C | -3.95189824633246 | 0.68780182495195 | -0.12024371896655 |
| C | -3.96936544630035 | -0.51522370940242 | 0.50154746212983 |
| C | 0.09689507503996 | -4.96063801549148 | 0.61736188638752 |
| C | 0.17302909058302 | 4.96230233947447 | -0.06662095740645 |
| H | 3.21066160351901 | -2.95194856739489 | -0.64678144087105 |
| H | 2.13895157561739 | -2.00919453198113 | -1.68446866059659 |
| H | 2.11412694080692 | -4.84477824989436 | -0.11005708341445 |
| H | -1.91335528157941 | -4.69984695798052 | 1.31905774454038 |

| | | | |
|---|---|---|---|
| H | -3.01551588041271 | -2.76487493900858 | 1.44658839809787 |
| H | -1.92902229617680 | -1.75297190112432 | 2.38566071904375 |
| H | -2.92268887729186 | 2.84834785074471 | -1.19330006104934 |
| H | -1.80378410783041 | 1.80682969024233 | -2.06775743916666 |
| H | -1.76878088519060 | 4.77563038127525 | -0.96352176834137 |
| H | 2.08386636337821 | 4.77757918354921 | 0.89433559223726 |
| H | 3.05339582206838 | 2.86444222148538 | 1.53481952833578 |
| H | 1.83103037585415 | 1.92599991430796 | 2.37701918926787 |
| H | -0.78462089833580 | -0.79806730425287 | -5.28657247832320 |
| H | 0.48137764796439 | 0.44315832432087 | -5.44263359472435 |
| H | -1.18177964697345 | 0.91317245703750 | -5.04664450917573 |
| H | 4.97275893138423 | -1.37640146657256 | 0.20585017751783 |
| H | 4.88667044492290 | 1.17136807304030 | 1.25885312452422 |
| H | -4.75715137086282 | 1.33490899090250 | -0.41987965305984 |
| H | -4.79145713149439 | -1.10545798812091 | 0.86591334386195 |
| H | 0.09857593095695 | -6.03605126121241 | 0.73575290932416 |
| H | 0.21004011506214 | 6.04089753838973 | -0.14160812734517 |

**$[Cu^{III}(NHC_2)]^{3+}$**

Charge = 3+, Spin Multiplicity = 1

| | | | |
|---|---|---|---|
| Cu | -0.07557324534937 | 0.08473907599887 | 0.01749033748120 |
| N | -0.11710427142914 | -1.92599544467153 | 0.18499604266301 |
| N | -0.03408855310710 | 2.09834319628169 | 0.15192748564677 |
| N | -0.06374217098734 | 0.01037443838644 | -2.35450306979143 |
| C | -1.96331455466345 | 0.09258358873240 | 0.11688310543760 |
| C | 1.81096597131832 | 0.07711566686513 | 0.16051460223402 |
| N | -2.77232298670605 | -0.82451915396435 | -0.40362265527385 |
| N | -2.72203492841614 | 0.98952662489733 | 0.73832089974582 |
| N | 2.63204382830595 | 0.97882855247198 | -0.36652835933578 |
| N | 2.55228490983997 | -0.79376132700535 | 0.83765055387342 |
| C | 0.87877312657885 | -2.59424048389109 | 0.82659624639028 |
| C | -1.09389580910824 | -2.63335378563464 | -0.43741378792053 |
| C | -4.09293111422718 | -0.50350057690443 | -0.11784575943584 |
| C | -2.25768058060771 | -1.96631986720113 | -1.13886379999260 |
| C | -4.06096674973948 | 0.64521354291859 | 0.60729739372831 |
| C | -2.13349050179208 | 2.06810777191251 | 1.52145382510592 |
| C | -1.02672185965424 | 2.78103419215742 | 0.78058873325227 |
| C | 0.97591682431046 | 2.79223106044453 | -0.43609813244297 |
| C | 2.11844492599363 | 2.09608390253589 | -1.14279545936202 |
| C | 3.94375593143155 | 0.67702411647537 | -0.02512379657418 |
| C | 3.89331171515817 | -0.44723814095791 | 0.73785917581180 |
| C | 1.93263293339283 | -1.85756569651206 | 1.61918072477104 |
| C | -0.09402854849986 | -0.10324945645329 | -3.49935864804762 |

| | | | |
|---|---|---|---|
| C | 0.93675857382873 | -3.97870713513175 | 0.81292299897897 |
| C | -1.05987802315533 | -4.02119018052970 | -0.47321905287160 |
| C | -1.03533297770752 | 4.16774288692284 | 0.81203754717493 |
| C | 0.99323556893107 | 4.17966249677476 | -0.42356411948387 |
| C | -0.14275362167436 | -0.25197924607384 | -4.93928720316056 |
| C | -0.02976206203020 | -4.70955078177724 | 0.14198565773654 |
| C | -0.02058348487005 | 4.88439121350562 | 0.20157150153427 |
| H | -4.92310351946544 | -1.10006077819544 | -0.45629818867217 |
| H | -1.95560604924565 | -1.63971717101380 | -2.13446042588483 |
| H | -3.06023219546215 | -2.69003245669721 | -1.24701954315383 |
| H | -4.85678960071782 | 1.22669557552605 | 1.04110113890439 |
| H | -1.75359426036551 | 1.65753061436998 | 2.46168888551861 |
| H | -2.91483345337440 | 2.78007962827257 | 1.76997881816347 |
| H | 1.79037051401078 | 1.72945201992668 | -2.11654112956889 |
| H | 2.92484818011919 | 2.80617806828481 | -1.29933571930475 |
| H | 4.78209301368428 | 1.26733943187705 | -0.35442103291303 |
| H | 4.67697464507442 | -1.00891539796587 | 1.21761859523896 |
| H | 1.49438532830543 | -1.42573816220245 | 2.52335930794152 |
| H | 2.70413584773577 | -2.55494548654925 | 1.93135083606764 |
| H | 1.74464354535760 | -4.47883796986312 | 1.32916578102452 |
| H | -1.85262374946789 | -4.55439654421655 | -0.98058415894477 |
| H | -1.84120594589389 | 4.67892136199888 | 1.32084502853338 |
| H | 1.81370984554407 | 4.70073799760100 | -0.89806255753123 |
| H | -0.88708120023241 | -1.00215103290511 | -5.21138561163383 |
| H | 0.83174306785771 | -0.57077220076510 | -5.31418354922233 |
| H | -0.41730287074995 | 0.69834839761058 | -5.40116043977222 |
| H | 0.01265717110420 | -5.79086753088648 | 0.11307512896297 |
| H | -0.01545065818304 | 5.96677143621972 | 0.22140529737308 |

**[Cu$^{III}$(NHC$_4$)]$^{3+}$**

Charge = 3+, Spin Multiplicity = 1

| | | | |
|---|---|---|---|
| Cu | 0.03229808776766 | -0.00802812810694 | 0.11864237027951 |
| N | -2.72236441013310 | -1.01822590913991 | -0.14960253623219 |
| N | -2.70775966645164 | 1.05165990371497 | 0.36015140049886 |
| N | -0.99182677179212 | 2.73663661758113 | 0.42799817821532 |
| N | 1.10074448186391 | 2.73600010808165 | 0.01540624132707 |
| N | 2.78810731813870 | 1.01965621725733 | -0.04001317343073 |
| N | 2.76758880744168 | -1.08680411723597 | 0.29827934168672 |
| N | 1.05070082187890 | -2.77168505299039 | 0.22578239216403 |
| N | -1.03708489976242 | -2.73351942313217 | -0.20703421110259 |
| C | -1.89907920868679 | 0.01048829559839 | 0.10758493656803 |
| C | -4.05775312012779 | -0.63485251059755 | -0.05570934873197 |
| C | -4.04869988195395 | 0.68771926887409 | 0.26135656080057 |
| C | -5.19138504167512 | -1.57367244889198 | -0.25897594410857 |
| H | -6.12600910882298 | -1.06898772618353 | -0.02356893674340 |
| H | -5.10536620157276 | -2.43969702346000 | 0.40068144816933 |
| H | -5.24528614831799 | -1.93546636579990 | -1.28849847318689 |
| C | -5.17009958081677 | 1.64258438237474 | 0.45740994034379 |
| H | -6.11029521854440 | 1.15027837403222 | 0.21767312335027 |
| H | -5.06873379092374 | 2.50662916473243 | -0.20273830230252 |
| H | -5.22470287823333 | 2.00610692132287 | 1.48623488531919 |
| C | -2.31219154250611 | 2.34561998129228 | 0.87111040795094 |
| H | -2.33709253692771 | 2.32833531268671 | 1.96202245862370 |
| H | -3.02204397616674 | 3.08265205266051 | 0.51020289364037 |
| C | 0.04929811497074 | 1.92107752399187 | 0.19712738025223 |
| C | -0.60614421139231 | 4.07400698785271 | 0.38639837453204 |
| C | 0.72966576443080 | 4.07370170489001 | 0.13124650642465 |
| C | -1.55511458342522 | 5.20210040963273 | 0.57063851288606 |
| H | -1.04222487275064 | 6.14195952138863 | 0.37751493107682 |
| H | -1.96282940532215 | 5.23351241760329 | 1.58376877477134 |
| H | -2.39064239485964 | 5.12937486658476 | -0.12870112140424 |
| C | 1.68813126636855 | 5.20216727639866 | 0.00619637728472 |
| H | 1.18240771468055 | 6.13518675668267 | 0.24578176616021 |
| H | 2.09832373591545 | 5.28245937720486 | -1.00309526977729 |
| H | 2.52151920070277 | 5.08699086027251 | 0.70269328150244 |
| C | 2.41612631650466 | 2.35588877618411 | -0.45226475926732 |
| H | 2.44440679944015 | 2.41932726919842 | -1.54145435455628 |
| H | 3.13762346138938 | 3.05191888289775 | -0.03670258513487 |
| C | 1.96242168578846 | -0.02585449938165 | 0.12790937212463 |
| C | 4.12236796040711 | 0.62549381989688 | 0.02620426476268 |
| C | 4.10933926116591 | -0.71846057305062 | 0.23343204507798 |
| C | 5.26123619714263 | 1.57192141960304 | -0.09399483575030 |

| | | | |
|---|---|---|---|
| H | 6.19205370099001 | 1.04611167414192 | 0.10763270649858 |
| H | 5.17415179500235 | 2.38419019165571 | 0.63054019690890 |
| H | 5.32398484517666 | 2.01323409360016 | -1.09136045474970 |
| C | 5.22958594875976 | -1.68700759949008 | 0.35361922666727 |
| H | 6.17097695510800 | -1.17776251116382 | 0.15862651447087 |
| H | 5.13053328307037 | -2.49341065413460 | -0.37621741315490 |
| H | 5.27957754154770 | -2.13449804127892 | 1.34893919740984 |
| C | 2.36828377879116 | -2.41610887998863 | 0.70653077817220 |
| H | 2.38602835536991 | -2.48132685080366 | 1.79578885642928 |
| H | 3.08034367616995 | -3.12447702349841 | 0.29596120001931 |
| C | 0.01598945350023 | -1.93740482798923 | 0.03696398141756 |
| C | 0.65782363088973 | -4.10203986401947 | 0.09946190899722 |
| C | -0.67541172759528 | -4.07776919045526 | -0.16798302768235 |
| C | 1.59657171863375 | -5.24699527430679 | 0.22480226222433 |
| H | 1.07710729473792 | -6.17042463433118 | -0.02226232696780 |
| H | 1.99983599748448 | -5.33883901388458 | 1.23589825924429 |
| H | 2.43561699786388 | -5.14185993409134 | -0.46630372824764 |
| C | -1.64163317753147 | -5.18853478484637 | -0.36743030588269 |
| H | -1.14780280834696 | -6.13731469890547 | -0.16818145322813 |
| H | -2.03474670552326 | -5.21195553448894 | -1.38662598982001 |
| H | -2.48586625839380 | -5.10223468886956 | 0.31952199784533 |
| C | -2.34451243479010 | -2.31620510404550 | -0.66324650498473 |
| H | -3.07156508050730 | -3.04201412473838 | -0.31438641450005 |
| H | -2.35692955924031 | -2.29174160558918 | -1.75433040715131 |

**$[Cu^{III}(CF_3)_4]^-$**

Charge = 1-, Spin Multiplicity = 1

| | | | |
|---|---|---|---|
| C | -0.57888321333209 | -0.87192810770068 | -4.61669655249915 |
| C | 1.24870669476421 | -3.05020976763769 | -4.40963955185390 |
| C | 1.47833807231003 | 0.87018718488180 | -3.72877744788674 |
| C | 3.37418382159552 | -1.11507810433457 | -4.53219278742627 |
| Cu | 1.38496409074348 | -1.05037661636376 | -4.31751573386117 |
| F | 3.87088529843570 | 0.02400621230627 | -5.10562741861118 |
| F | 3.79458304414789 | -2.10584524235780 | -5.37497292534176 |
| F | 4.08208039740395 | -1.28693565968239 | -3.38227260075224 |
| F | 2.51557920676023 | 1.11013329617410 | -2.87255884171744 |
| F | 1.61325680072138 | 1.77897519107215 | -4.73225108258272 |
| F | 0.37859040641269 | 1.26585090019746 | -3.01888290997497 |
| F | -1.08831195537215 | -1.80859502505455 | -5.47048544593969 |
| F | -0.89399280688326 | 0.31876306969050 | -5.21409482456034 |
| F | -1.34336207842653 | -0.94156858200232 | -3.49436571889668 |
| F | 0.13488502680767 | -3.54372229366747 | -3.78682883279539 |
| F | 1.22150934303065 | -3.56228088779489 | -5.66753459725558 |

F 2.27957453288055 -3.68768122272611 -3.77795042304449

# References


(1) Liu, Y.; Resch, S. G.; Klawitter, I.; Cutsail III, G. E.; Demeshko, S.; Dechert, S.; Kühn, F. E.; DeBeer, S.; Meyer, F. An Adaptable N-Heterocyclic Carbene Macrocycle Hosting Copper in Three Oxidation States. *Angew. Chemie Int. Ed.* **2020**, *59* (14), 5696–5705. https://doi.org/10.1002/anie.201912745.

(2) Romine, A. M.; Nebra, N.; Konovalov, A. I.; Martin, E.; Benet-Buchholz, J.; Grushin, V. V. Easy Access to the Copper(III) Anion [Cu(CF )4]−. *Angew. Chemie Int. Ed.* **2015**, *54* (9), 2745–2749. https://doi.org/10.1002/anie.201411348.

(3) Bernd, M. A.; Dyckhoff, F.; Hofmann, B. J.; Böth, A. D.; Schlagintweit, J. F.; Oberkofler, J.; Reich, R. M.; Kühn, F. E. Tuning the Electronic Properties of Tetradentate Iron-NHC Complexes: Towards Stable and Selective Epoxidation Catalysts. *J. Catal.* **2020**, *391*, 548–561. https://doi.org/10.1016/j.jcat.2020.08.037.

(4) Ghavami, Z. S.; Anneser, M. R.; Kaiser, F.; Altmann, P. J.; Hofmann, B. J.; Schlagintweit, J. F.; Grivani, G.; Kühn, F. E. A Bench Stable Formal Cu(III): N-Heterocyclic Carbene Accessible from Simple Copper(II) Acetate. *Chem. Sci.* **2018**, *9* (43), 8307–8314. https://doi.org/10.1039/c8sc01834k.

(5) Sheldrick, G. M. SHELXT - Integrated Space-Group and Crystal-Structure Determination. *Acta Crystallogr. Sect. A Found. Crystallogr.* **2015**, *71* (1), 3–8. https://doi.org/10.1107/S2053273314026370.

(6) SADABS; Bruker AXS GmbH. Karlsruhe, Germany 2016.

(7) Ravel, B.; Newville, M. ATHENA, ARTEMIS, HEPHAESTUS: Data Analysis for X-Ray Absorption Spectroscopy Using IFEFFIT. *J. Synchrotron Radiat.* **2005**, *12* (4), 537–541. https://doi.org/10.1107/S0909049505012719.

(8) Neese, F. The ORCA Program System. *Wiley Interdiscip. Rev. Comput. Mol. Sci.* **2012**, *2* (1), 73–78. https://doi.org/10.1002/wcms.81.

(9) Neese, F. Software Update: The ORCA Program System, Version 4.0. *Wiley Interdiscip. Rev. Comput. Mol. Sci.* **2018**, *8* (1), 4–9. https://doi.org/10.1002/wcms.1327.

(10) Lee, C.; Yang, W.; Parr, R. G. Development of the Colic-Salvetti Correlation-Energy Formula into a Functional of the Electron Density. *Phys. Rev. B* **1988**, *37* (2), 785–788.

(11) Becke, A. D. Density-Functional Thermochemistry. III. The Role of Exact Exchange. *J. Chem. Phys.* **1993**, *98* (7), 5648–5652. https://doi.org/10.1063/1.464913.

(12) Weigend, F.; Ahlrichs, R. Balanced Basis Sets of Split Valence, Triple Zeta Valence and Quadruple Zeta Valence Quality for H to Rn: Design and Assessment of Accuracy. *Phys. Chem. Chem. Phys.* **2005**, *7* (18), 3297–3305. https://doi.org/10.1039/b508541a.

(13) Stoychev, G. L.; Auer, A. A.; Neese, F. Automatic Generation of Auxiliary Basis Sets. *J. Chem. Theory Comput.* **2017**, *13* (2), 554–562. https://doi.org/10.1021/acs.jctc.6b01041.

(14) Neese, F.; Wennmohs, F.; Hansen, A.; Becker, U. Efficient, Approximate and Parallel Hartree-Fock and Hybrid DFT Calculations. A “chain-of-Spheres” Algorithm for the Hartree-Fock Exchange. *Chem. Phys.*

**2009**, *356* (1–3), 98–109. https://doi.org/10.1016/j.chemphys.2008.10.036.

(15) Klamt, A.; Schüürmann, G. COSMO: A New Approach to Dielectric Screening in Solvents with Explicit Expressions for the Screening Energy and Its Gradient. *J. Chem. Soc. Perkin Trans. 2* **1993**, No. 5, 799–805. https://doi.org/10.1039/P29930000799.

(16) Van Lenthe, E.; Wormer, P. E. S.; Van Der Avoird, A. Density Functional Calculations of Molecular G-Tensors in the Zero-Order Regular Approximation for Relativistic Effects. *J. Chem. Phys.* **1997**, *107* (7), 2488–2498. https://doi.org/10.1063/1.474590.

(17) Pantazis, D. A.; Chen, X. Y.; Landis, C. R.; Neese, F. All-Electron Scalar Relativistic Basis Sets for Third-Row Transition Metal Atoms. *J. Chem. Theory Comput.* **2008**, *4* (6), 908–919. https://doi.org/10.1021/ct800047t.

(18) Gasparro, F. P.; Kolodny, N. H. NMR Determination of the Rotational Barrier in N,N-Dimethylacetamide. A Physical Chemistry Experiment. *J. Chem. Educ.* **1977**, *54* (4), 258. https://doi.org/10.1021/ed054p258.

(19) Bill, E. JulX, Program for Simulation of Molecular Magnetic Data. Max-Planck Institute for Chemical Energy Conversion: Mülheim/Ruhr 2008.

(20) Pollock, C. J.; Lancaster, K. M.; Finkelstein, K. D.; DeBeer, S. Study of Iron Dimers Reveals Angular Dependence of Valence-to-Core X-Ray Emission Spectra. *Inorg. Chem.* **2014**, *53* (19), 10378–10385. https://doi.org/10.1021/ic501462y.

(21) Phu, P. N.; Gutierrez, C. E.; Kundu, S.; Sokaras, D.; Kroll, T.; Warren, T. H.; Stieber, S. C. E. Quantification of Ni-N-O Bond Angles and NO Activation by X-Ray Emission Spectroscopy. *Inorg. Chem.* **2021**, *60* (2), 736–744. https://doi.org/10.1021/acs.inorgchem.0c02724.
